# Formation and metabolism of oxysterols and cholestenoic acids found in the mouse circulation: Lessons learnt from deuterium-enrichment experiments and the *CYP46A1* transgenic mouse

**DOI:** 10.1016/j.jsbmb.2019.105475

**Published:** 2019-12

**Authors:** Peter J. Crick, Eylan Yutuc, Jonas Abdel-Khalik, Ahmed Saeed, Christer Betsholtz, Guillem Genove, Ingemar Björkhem, Yuqin Wang, William J. Griffiths

**Affiliations:** a Swansea University Medical School, ILS1 Building, Singleton Park, Swansea SA2 8PP, Wales, UK; b Department of Laboratory Medicine, Division of Clinical Chemistry, Karolinska University Hospital, Karolinska Institutet, 141 86 Huddinge, Sweden; c ICMC Karolinska Institutet, Novum, 141 57 Huddinge, Sweden

**Keywords:** 24S-hydroxycholesterol, 24S,25-epoxycholesterol, CYP46A1, Bile acid biosynthesis, Deuterium-enrichment, Deuterium-isotope effect, Liquid chromatography–mass spectrometry, Derivatisation

## Abstract

While the presence and abundance of the major oxysterols and cholestenoic acids in the circulation is well established, minor cholesterol metabolites may also have biological importance and be of value to investigate. In this study by observing the metabolism of deuterium-labelled cholesterol in the pdgfb^ret/ret^ mouse, a mouse model with increased vascular permeability in brain, and by studying the sterol content of plasma from the *CYP46A1* transgenic mouse overexpressing the human cholesterol 24S-hydroxylase enzyme we have been able to identify a number of minor cholesterol metabolites found in the circulation, make approximate-quantitative measurements and postulate pathways for their formation. These “proof of principle” data may have relevance when using mouse models to mimic human disease and in respect of the increasing possibility of treating human neurodegenerative diseases with pharmaceuticals designed to enhance the activity of CYP46A1 or by adeno-associated virus delivery of *CYP46A1*.

## Highlights

• Pathway from 24S-HC towards bile acids.
• Non-cerebral CYP46A1-idipendent biosynthesis of 24R-HC.
• Different pathways used in the formation of 3β-HCA and 7αH,3O-CA.

## 1 Introduction

Oxysterols are oxidised forms of cholesterol, or of its precursors [1,2]. There is a growing interest in these molecules based on their biological activities as ligands to, or modulators of, nuclear receptors [[3], [4], [5], [6], [7]], G protein-coupled receptors (GPCRs) [[8], [9], [10], [11], [12], [13]], of *N-*methyl-D-aspartate receptors (NMDARs) [14] and of cholesterol biosynthesis, through binding to INSIG (insulin induced gene) [15]. Cholestenoic acids, i.e. acidic oxysterols, have been ascribed neuroprotective or neurotoxic properties depending on their structures [16,17], are also ligands to nuclear receptors [16,18,19], and the ultimate metabolites of oxysterols, i.e. bile acids, are also biologically active as ligands to nuclear receptors [[20], [21], [22], [23]] and GPCRs [24]. Both oxysterols and cholestenoic acids represent transport forms of cholesterol, transporting sterol to the liver for further metabolism or excretion via the bile. By administration of deuterated cholesterol to a healthy human volunteer metabolic relationships between different oxysterols and cholestenoic acids have been established [25,26], but extensive studies with deuterated cholesterol have yet to be made on laboratory animals [27,28].

In human and mouse circulation the dominating oxysterols include 4β-hydroxycholesterol (4β-HC), 7α-hydroxycholesterol (7α-HC), 24S-hydroxycholesterol (24S-HC), (25R)26-hydroxycholesterol (26-HC, also known as 27-HC, see Supplemental Table S1 for common and systematic names of sterols) [[29], [30], [31]], formed from cholesterol by the cytochrome P450 (CYP) enzymes CYP3A4, 7A1, 46A1 and 27A1, respectively [[32], [33], [34], [35]]. Minor oxysterols formed from cholesterol precursors include 24S,25-epoxycholesterol (24S,25-EC) generated from CYP46A1 oxidation of desmosterol, or via a shunt pathway in parallel to cholesterol biosynthesis [36,37], cholesterol-7,8-epoxide (7,8-EC) and 7-oxocholesterol (7-OC, also known as 7-ketocholesterol) both formed by CYP7A1 oxidation of 7-dehydrocholesterol (7-DHC) [38,39]. 7α-HC and 7-OC can also be formed via non-enzymatic reactions from cholesterol, as may 7β-hydroxycholesterol (7β-HC), while 5,6-epoxycholesterol (5,6-EC) has to-date only been reported to be formed via non-enzymatic reactions [40].

In human and mouse circulation, and in cerebrospinal fluid (CSF), the cholestenoic acids 3β-hydroxycholest-5-en-(25R)26-oic (3β-HCA) and 7α-hydroxy-3-oxocholest-4-en-(25R)26-oic acid (7αH,3O-CA, 25R stereochemistry is assumed unless indicated otherwise) are abundant, so is 3β,7α-dihydroxycholest-5-en-(25R)26-oic acid (3β,7α-diHCA) in human but not in mouse [16,19,26,31,[41], [42], [43], [44]].

Interestingly, increasing the cerebral activity of CYP46A1 has been suggested to have therapeutic potential towards neurodegenerative disease. Mast at al. have proposed CYP46A1 as a pharmacological target for Alzheimer’s disease (AD) [45], while Burlot et al. have shown that adeno-associated virus (AAV) delivery of *CYP46A1* to an AD mouse model rescued cognitive defects associated with the model [46]. Boussicault et al. found a similar AAV-*CYP46A1* delivery to reduce neuronal atrophy and motor defects in a mouse model of Huntington’s disease [47]. On the other hand, inhibition of CYP27A1, the enzyme required to introduce the (25R)26-carboxylate group into the cholesterol skeleton, has been suggested as a potential therapeutic towards age-related neurodegenerative disease and also breast cancer [48]. Importantly, Mast et al have shown that a number of existing pharmaceutical drugs can inhibit CYP27A1 [49].

One way to learn more about the metabolic origin of different oxysterols and cholestenoic acids is to monitor their formation *in vivo* from deuterated cholesterol. This approach has previously been adopted to investigate the formation of a number of cholestenoic acids in man and of oxysterols in man and rodents [[25], [26], [27]]. In this way Meaney at al. defined different metabolic pathways from cholesterol to 3β,7α-diHCA and 7αH,3O-CA in man, where the initial oxidation of cholesterol to 3β-HCA on its way to 3β,7α-diHCA is likely to be pulmonary, while hepatic oxidation of cholesterol to 7α-HC is likely to be the first step in the pathway towards 7αH,3O-CA [26]. Whether, or not, a similar disparity of pathways exists in mouse has yet to be established. To investigate metabolic pathways further, knockout mice can be studied and it was in this way the enzymes required to biosynthesise numerous different oxysterols were established [50,51]. An additional genetic tool is to overexpress sterol metabolising enzymes in a transgenic (tg) mouse. This strategy has been adopted in mice overexpressing human CYP27A1 or CYP46A1 [52,53].

Here, to investigate whether the metabolic relationships established in man are similar to those found in mouse we have performed an extended analysis of the oxysterol and cholestenoic acid content of plasma, utilising more specific and sensitive methods than previously available, to extract additional metabolic information about oxysterols and cholestenoic acids from a unique isotope experiment on a mouse with a leaking blood-brain barrier [28] and a mouse model with overexpressed *CYP46A1* and high levels of 24S-hydroxycholesterol in the circulation [53]. Admittedly, the low number of animals used in this study is a limitation, however, our intention is to identify existing minor metabolic pathways without evaluating their importance. We are aware that more animals would be required in order to evaluate the importance of the new pathways. We use a derivatisation strategy called enzyme-assisted derivatisation for sterol analysis (EADSA), where sterols with a 3β-hydroxy-5-ene structure are oxidised with cholesterol oxidase to 3-oxo-4-ene sterols and then derivatised with the Girard P (GP) hydrazine reagent [[54], [55], [56]] for liquid chromatography (LC) - mass spectrometry (MS) with multistage fragmentation (MS^n^) analysis. This greatly increases the sensitivity for sterol analysis and aids in structure identification through LC—MS and MS^n^. Sterols with a native oxo group are derivatised with GP reagent in the absence of cholesterol oxidase. To allow analysis via a single LC–MS(MS^n^) injection we use different isotopic forms of GP reagent. [^2^H_5_]GP was used with cholesterol oxidase treated sterols and [^2^H_0_]GP for derivatisation in the absence of cholesterol oxidase (see Supplemental Fig. S1).

## 2 Materials and methods

### 2.1 Animals

Male tg mice overexpressing human *CYP46A1* (*CYP46A1*tg) were as described in Shafaati et al [53] and were from the study previously reported by Saeed et al [28]. In addition, a single male *pdgfb*^ret/ret^ mouse bred on a C56BL/6 background was fed for 40-days on a chow diet containing 0.3% [26,26,26,27,27,27-^2^H_6_]cholesterol. This animal was used in the previous study of Saeed et al as a pericyte-deficient mouse model to investigate the effect of a leaking blood brain barrier (BBB) on cholesterol metabolism in brain [28]. Male, wild type (WT) mice were 12 weeks old C57BL/6 J from the Jackson Laboratory, US. All experimental procedures in this study were in compliance with National Institutes of Health Guide for Care and Use of Laboratory Animals, the European Communities Council Directive of 24 November 1986 (86/609/EEC) and approved by The Northern Stockholm Research Animal Ethics Committee.

### 2.2 Materials

Stable-isotope labelled oxysterols [25,26,26,26,27,27,27-^2^H_7_]7α-HC ([^2^H_7_]7α-HC), [25,26,26,26,27,27,27-^2^H_7_]22R-hydroxycholesterol ([^2^H_7_]22R-HC), [25,26,26,26,27,27,27-^2^H_7_]24R/S-HC ([^2^H_7_]24R/S-HC), [26,26,26,27,27,27-^2^H_6_]7α,25-dihydroxycholesterol ([^2^H_6_]7α,25-diHC) and [25,26,26,26,27,27,27-^2^H_7_]cholesterol were from Avanti Polar Lipids (Al, USA). [25,26,26,26,27,27,27^2^H_7_]22R-Hydroxycholest-4-en-3-one ([^2^H_7_]22R-HCO) was prepared by cholesterol oxidase treatment of [^2^H_7_]22R-HC. 3β,7α,24S-Trihydroxycholest-5-en-(25R)26-oic acid (3β,7α,24S-triHCA) was also from Avanti Polar Lipids. Other oxysterols and cholestenoic acids were from previous studies or as indicated in Supplemental Table S1 [[54], [55], [56]]. [^2^H_0_]GP was from TCI Europe (Belgium), [^2^H_5_]GP was synthesised as described in Crick et al [55]. Cholesterol oxidase enzyme from *Streptomyces sp* was from Sigma – Aldrich (now Merck) UK.

### 2.3 Extraction of oxysterols and cholestenoic acids from plasma

Plasma (20 – 100 μL) was added dropwise to ethanol (1.05 mL) containing internal standards [^2^H_7_]24R/S-HC, [^2^H_7_]22R-HCO, [^2^H_7_]7α-HC (20 ng each), [^2^H_6_]7α,25-diHC (2 ng) and [^2^H_7_]cholesterol (20 μg), with sonication. For studies performed on mice fed [^2^H_6_]cholesterol in the diet, [^2^H_7_]24R/S-HC and [^2^H_7_]cholesterol were omitted from the internal standard mixture to avoid possible isotope overlap with [^2^H_6_]cholesterol and its metabolic products. Water was added to make-up the solution to 1.5 mL of 70% ethanol. After further sonication (5 min) the solution was centrifuged (17,000 g for 30 min) to remove any precipitated matter.

To separate cholesterol from more polar oxysterols and cholestenoic acids, to avoid the potential generation of cholesterol autoxidation artefacts during sample handling, the supernatant from above was loaded onto a washed (4 mL ethanol) and conditioned (6 mL 70% ethanol) 200 mg Certified Sep-Pak C_18_ solid phase extraction (SPE) column (Waters UK). The flow-through (1.5 mL) was combined with a column wash (5.5 mL 70% ethanol) to give SPE1-Fr-1 (7 mL 70% ethanol) rich in oxysterols and cholestenoic acids. The column was washed with a further 4 mL of 70% ethanol to give SPE1-Fr-2, before cholesterol, and sterols of similar polarity, were eluted from the column with 2 mL of ethanol to give SPE1-Fr-3. Each fraction was then divided into two equal proportions (a) and (b) and dried under vacuum.

### 2.4 Enzyme-assisted derivatisation for sterol analysis (EADSA)

Oxysterols and cholestenoic acids were derivatised using EADSA technology as described previously (Supplemental Fig. S1) [[54], [55], [56]]. In brief, fraction SPE1-Fr-1(a) was re-dissolved in propan-2-ol (100 μL). Phosphate buffer (50 mM, pH 7, 1 mL) containing cholesterol oxidase enzyme (3 μL, 2 mg/mL in water, 44 u/mg protein) from *Streptomyces sp* was added and the mixture left for 1 h at 37 °C. The reaction was quenched with 2 mL of methanol. Glacial acetic acid (150 μL) was added followed by [^2^H_5_]GP reagent (190 mg as the bromide salt). After vortexing the derivatisation reaction was allowed to proceed overnight in the dark. Next morning, excess derivatisation reagent was removed by SPE on a 60 mg Oasis HLB column (Waters UK), previously washed with methanol (6 mL), 10% methanol (6 mL) and conditioned with 70% methanol (4 mL). The derivatisation mixture (3.25 mL, 70% organic) was loaded on the column followed by a 1 mL wash (70% methanol) of the reaction vessel. The column was rinsed and conditioned with 35% methanol (1 mL) and the combined effluent (5.25 mL) diluted with water (4 mL) to give a 35% methanol solution. This solution was re-cycled through the column and the procedure repeated to give a 17.5% methanol solution which was once more re-cycled through the column. At this point all GP-derivatised oxysterols and cholestenoic acids are extracted by the column and unreacted GP-reagent eluted to waste. The column was finally washed with 10% methanol (6 mL) and derivatised oxysterols, cholestenoic acids and other sterols eluted in 3 × 1 mL of 100% methanol and 1 mL of ethanol. Oxysterols and cholestenoic acids elute in the first two 1 mL fractions of methanol which were combined to give SPE2-Fr-1 + 2(a). The entire procedure was repeated for SPE1-Fr-1(b), but in the absence of cholesterol oxidase and using [^2^H_0_]GP (150 mg, chloride salt) rather than [^2^H_5_]GP, to give SPE2-Fr-1 + 2(b). SPE2-Fr-1 + 2(a) was then combined with SPE2-Fr-1 + 2(b) diluted to 60% methanol and immediately analysed by LC–MS(MS)^n^.

### 2.5 LC–MS(MS^n^)

LC–MS(MS^n^) was performed using an Ultimate 3000 LC system (Dionex, now Thermo Fisher Scientific, UK) linked to an Orbitrap Velos (Thermo Scientific) linear ion trap (LIT) – Orbitrap hybrid mass spectrometer via an electrospray probe. The chromatographic separation was on a reversed phase Hypersil Gold C_18_ column (1.9 μm, 50 x 2.1 mm, Thermo Fisher Scientific) utilising a methanol/acetonitrile/formic acid gradient at flow-rate 200 μL/min. Mobile phase A was 33.3% methanol, 16.7% acetonitrile containing 0.1% formic acid. Mobile phase B was 63.3% methanol, 31.7% acetonitrile containing 0.1% formic acid. The gradient employed is indicated in Supplemental Table S2.

For each injection up-to 5 scan events were performed. One high-resolution scan was performed in the Orbitrap (60,000 FWHM definition at *m/z* 400) in parallel to 2–4 MS^n^ scans in the LIT. For MS^2^ normalised collision energy was 30% and for MS^3^ 35%. Sterols were identified based on measurement of accurate mass (±5 ppm), MS^3^ spectra and retention time, with comparison to authentic standards (see Supplemental Tables 1 and 3). In the absence of authentic standards presumptive identifications were based on these three parameters and are described in detail in Supplemental Text. Quantification was via high-resolution MS based on known amounts of added internal standard. The internal standard utilised for each analyte is indicated in Supplemental Tables S1 and S3. Previous studies have shown that side-chain oxysterols once derivatised with GP-hydrazine give an equivalent response factor [57]. Calibration curves showing a linear relationship between peak area and concentration have been reported earlier [55]. Strictly speaking, sterols, other than side-chain oxysterols and other oxysterols for which an authentic isotope-labelled standard was used, are approximately quantified in this study.

### 2.6 Calculation of enrichment in deuterium

Enrichment in deuterium of sterol S is calculated as the percent of the total species S present i.e.

(1) % Enrichment in deuterium = ([^2^H_n_]S / ([^2^H_n_]S + [^1^H]S))

The “corrected value” for enrichment in deuterium is calculated using eq. 2.

(2) % Corrected Enrichment in Deuterium = % Enrichment in deuterium for sterol S / % Enrichment in deuterium in cholesterol

## 3 Results and discussion

### 3.1 Oxysterols and cholestenoic acids derived from [^2^H_6_]cholesterol

Our first experiment was to investigate the different pools of cholesterol from which different oxysterols and cholestenoic acids are derived. This was achieved by feeding a single male *pdgfb*^ret/ret^ mouse with a diet rich in [^2^H_6_]cholesterol for 40 days [28]. The *pdgfb*^ret/ret^ mouse is a pericyte-deficient model with enhanced BBB permeability allowing an increased efflux of 24S-HC from brain. This facilitates the measurement of brain derived cholesterol metabolites in plasma, although the import of some cholesterol from the circulation to brain will result in an increased deuterium content of brain derived metabolites [28]. Our earlier study [28] showed the incorporation of deuterium in brain cholesterol after 40 days of feeding of the mouse was about 7%, corresponding to replacement of about 10% of brain cholesterol with plasma cholesterol under the conditions used. Some results from this earlier study have been reported previously, specifically for 24S-HC and 24R-HC following alkaline hydrolysis [28]. We now report new results for non-esterified (no alkaline hydrolysis) oxysterols and cholestenoic acids.

#### 3.1.1 Oxysterols

**Fig. 1:**
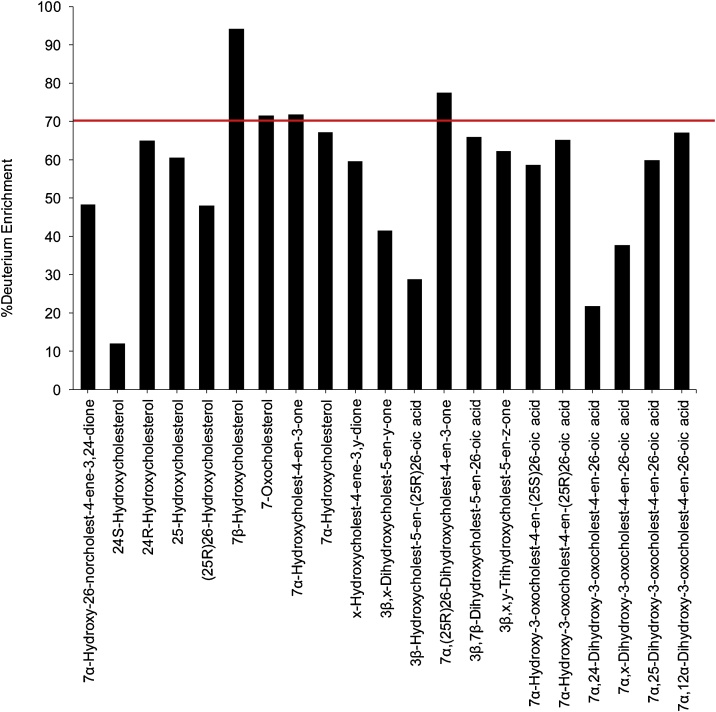
Deuterium enrichment in different oxysterols. The enrichment in cholesterol, reported in [28], is indicated by the dotted line.

Of the oxysterols known to be formed via enzymatic mechanisms from cholesterol, 7α-HC, 7α-hydroxycholest-4-en-3-one (7α-HCO) and 7α,(25R)26-dihydroxycholest-4-en-3-one (7α,26-diHCO) were found to be enriched with deuterium at a level of about 70%, equivalent to that of cholesterol reported earlier (Fig. 1 and Supplemental Table S1) [28]. This indicates that after the 40-day period these three oxysterols have been turned-over entirely i.e. deuterium enrichment is equivalent to that of cholesterol. 7-OC was also enriched with deuterium to about 70%, but an enzyme converting cholesterol to 7-OC has not been identified. It can, however, be formed via non-enzymatic mechanisms, which probably account for its formation in e.g. Niemann Pick disease type C [[58], [59], [60], [61]], and perhaps here also. Alternatively, 7-OC can be formed via oxidation of 7β-HC in a reaction catalysed by hydroxysteroid dehydrogenase 11B2 (HSD11B2) [12]. 7β-HC is not known to be formed via an enzymatic reaction from cholesterol, but like 7-OC is formed endogenously in lysosomal storage diseases, presumably via non-enzymatic reactions [60,61]. However, when analysing 7β-HC and 7-OC there is always a risk that they may be formed, at least in part, by *ex vivo* autoxidation of cholesterol [1]. This is likely to be true in this study as the degree of deuterium enrichment of 7β-HC (95%) was greater than that of cholesterol (70%).

**Fig. 2:**
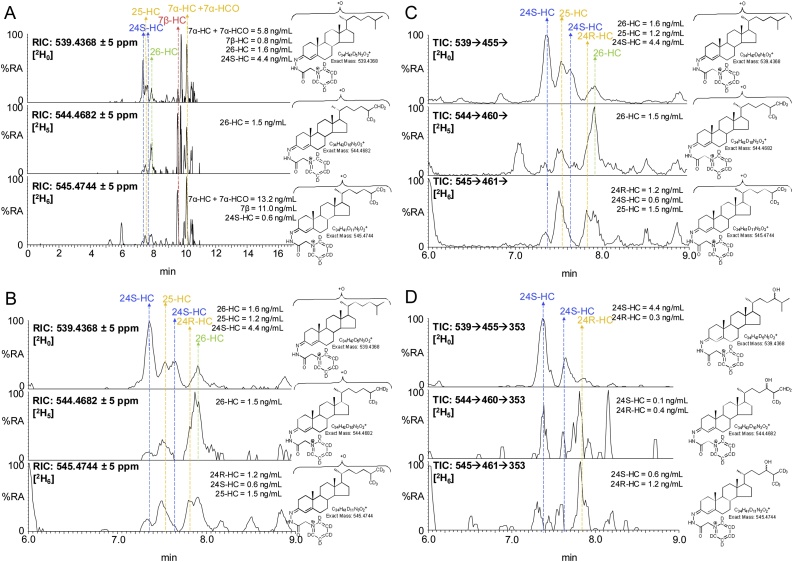
24S-HC and 26-HC show low levels of enrichment of deuterium in comparison to 24R-HC, 7α-HC and 7α-HCO. (A) Reconstructed ion chromatograms (RICs) for the [M]^+^ ions of [^2^H_0_] (*m/z* 539.4368, upper panel), [^2^H_5_] (*m/z* 544.4682, central panel), [^2^H_6_] (*m/z* 545.4744, lower panel) monohydroxysterols. (B) RICs as in (A) but over the retention time range for “side-chain” monohydroxycholesterols. (C) TICs for the [M]^+^→[M-Py]^+^→ transitions for [^2^H_0_] (539.4→455.4→, upper panel), [^2^H_5_] (544.5→460.4→, central panel), [^2^H_6_] (545.5→461.4→) over the retention time range for “side-chain” monohydroxycholesterols. (D) Multiple reaction monitoring (MRM) transitions [M]^+^→[M-Py]^+^→353.3 for [^2^H_0_] (539.4→455.4→353.3, upper panel), [^2^H_5_] (544.5→460.4→353.3, central panel) and [^2^H_6_] (545.5→461.4 →353.3, lower panel) isotopologues targeting 24-HC epimers. Labels are colour coded according to deuterium enrichment on a rainbow scale. Note [^2^H_6_] and [^2^H_5_] isotopologues elute slightly before their [^2^H_0_] analogues. See Supplemental Fig. S2 for MS^3^ spectra and Supplemental Fig. S3 for fragment-ion structures. The potential for autoxidation of cholesterol during sample preparation is discussed in reference [85].

**Fig. 3:**
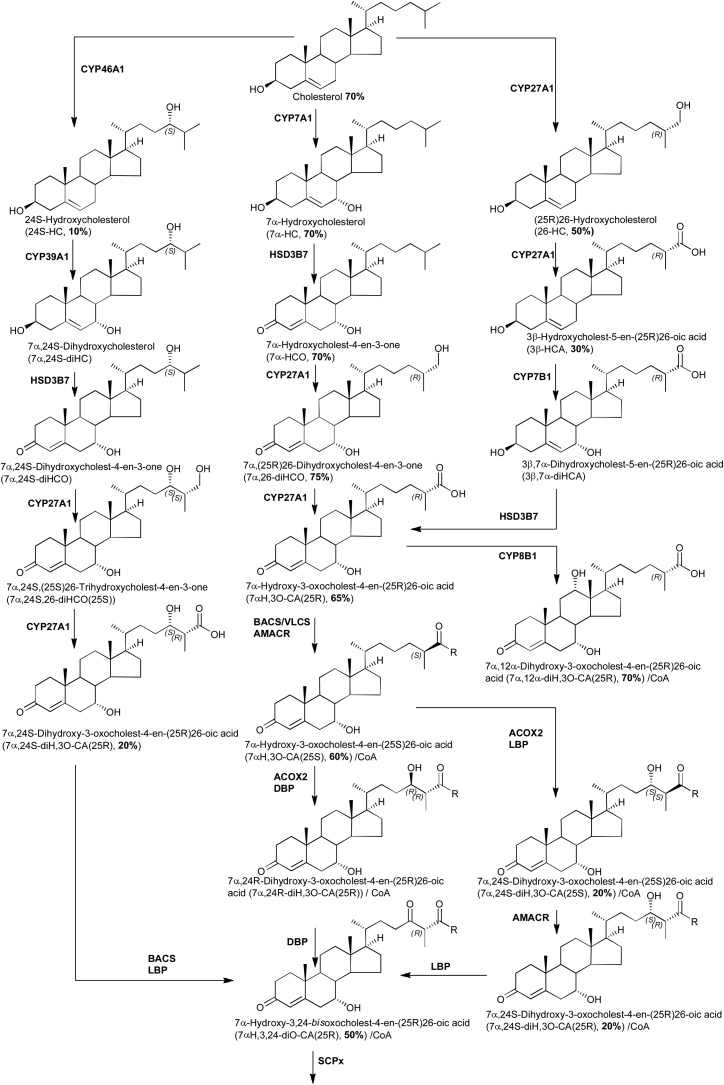
Biosynthetic route from cholesterol towards bile acids.

The degree of deuterium enrichment in 24S-HC, 26-HC and 25-hydroxycholesterol (25-HC) was about 10%, 50% and 60%, respectively (Fig. 1). The reduced degree of deuterium enrichment compared to cholesterol and its 7-oxygenated products (Fig. 2) suggests different origins in terms of pools of cholesterol for these side-chain hydroxylated sterols. When evaluating deuterium enrichment of oxysterols it is important to consider the deuterium isotope effect, where the breaking of a carbon-deuterium bond in the rate-determining step of a reaction mechanism will slow down the reaction. As a consequence of the deuterium isotope effect, hydroxylation at C-26 is reduced in [26,26,26,27,27,27-^2^H_6_]cholesterol compared to [^2^H_0_]cholesterol [28,62] and this can account for some of the reduced deuterium enrichment in 26-HC, but not in 25-HC or 24S-HC. It is interesting to note that a deuterium isotope effect does not appear to be evident in the formation of 7α,26-diHCO. This indicates that CYP27A1, the sterol (25R)26-hydroxylase, does not participate in the rate-determining step in the biosynthesis of 7α,26-diHCO, which is most likely formed via 7α-HC or 7α-HCO (Fig. 3). We have shown previously that 24-HC in mouse consists of two epimers, 24S-HC and 24R-HC, and that 80% of 24S-HC (measured after saponification of oxysterol esters) in the circulation is derived from brain while the remaining 20% and the entire 24R-epimer are derived from extracerebral sources [28]. This is consistent with the data from the current study for non-esterified 24S-HC where only 10% is found to be deuterated, compared to 70% for cholesterol. Taking this into account we calculate that about 85% of 24S-HC found in the circulation is derived from the non-exchangeable pool of cholesterol localised behind the BBB in brain. Here we can also confirm that the 24R-HC epimer is deuterated to a similar level as cholesterol, indicating its extracerebral formation.

**Fig. 4:**
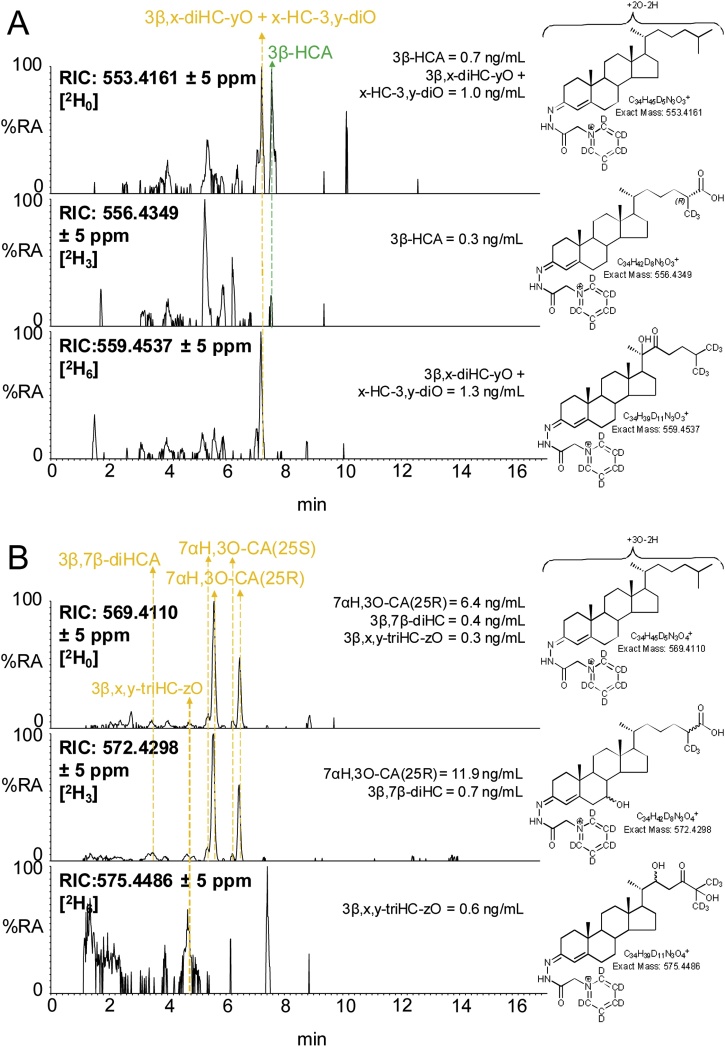
Enrichment of deuterium in cholestenoic acids and some minor oxysterols. (A) RICs for the [M]^+^ ions of [^2^H_0_] (*m/z* 553.4161, upper panel), [^2^H_3_] (*m/z* 556.4349, central panel), [^2^H_6_] (*m/z* 559.4537, lower panel) corresponding to 3β-HCA and to 3β,x-diHC-yO + x-HC-3,y-diO. (B) RICs for the [M]^+^ ions of [^2^H_0_] (*m/z* 569.4110, upper panel), [^2^H_3_] (*m/z* 572.4298, central panel), [^2^H_6_] (*m/z* 575.4486, lower panel) corresponding to 7αH,3O-CA, 3β,7β-diHCA and to 3β,x,y-triHC-zO. See Supplemental Fig. S4 for MS^3^ spectra.

Minor peaks observed in the LC–MS(MS)^n^ analysis of mouse plasma correspond to a 3β,x-dihydroxycholest-5-en-y-one (3β,x-diHC-yO) and x-hydroxycholest-4-ene-3,y-dione (x-HC-3,y-diO), possibly 3β,20-dihydroxycholest-5-en-22-one (3β,20-diHC-22O) and 20-hydroxycholest-4-ene-3,22-dione (20-HC-3,22-diO) or the 22-hydroxy-24-oxo isomers i.e. 3β,22-diHC-24O and 22-HC-3,24-diO (Fig. 4A and Supplemental Fig. S4A). These oxysterols are found to be hexadeuterated indicating an absence of oxidation on the terminal carbon atom of the side-chain, while the MS^3^ spectra suggest hydroxylation and carbonylation of the side-chain. See Supplemental Text for a detailed description of identifications from MS^3^ spectra. A third oxysterol also found to be hexadeuterated gave an MS^3^ spectrum of a 3β,x,y-trihydroxycholest-5-en-z-one (triHCO), possibly 3β,22,25-trihydroxycholest-5-en-24-one (3β,22,25-triHC-24O, Fig. 4B, Supplemental Fig. S4B). These three oxysterols were deuterated to about 40–60% indicating that they are not formed behind the BBB and with intact hexadeuteration, no deuterium isotope effect is possible (Supplemental Table S1).

#### 3.1.2 Cholestenoic acids

3β-HCA is biosynthesised from cholesterol via CYP27A1 catalysed oxidation, this requires the removal of three hydrogen atoms from C-26 (Fig. 3). Thus, when [26,26,26,27,27,27-^2^H_6_]cholesterol is the substrate, synthesis of 3β-HCA is attenuated as a consequence of the deuterium isotope effect. The result is reduced incorporation of deuterium into 3β-HCA i.e. 30% cf. 50% in 26-HC and 70% in cholesterol (Fig. 1, Supplemental Table S1, Fig. 4A).

Unlike 3β-HCA, the deuterium incorporation into 7αH,3O-CA and 3β,7β-dihydroxycholest-5-en(25R)26-oic acid (3β,7β-diHCA) is at about 65%, quite similar to that of cholesterol at 70%. This suggests that these two acids are derived in mouse from cholesterol via a different pathway to 3β-HCA and/or from a different pool of cholesterol (Fig. 3). While only CYP27A1 is involved in the biosynthesis 3β-HCA; CYP27A1, CYP7A1 or CYP7B1 and HSD3B7 are utilised to synthesise 7αH,3O-CA (Fig. 3). The similarity of deuterium incorporation in 7αH,3O-CA, 7α,26-diHCO, 7α-HCO and 7α-HC, and an absence of 3β,7α-diHCA in mouse plasma, suggests initial hydroxylation is at position 7α by CYP7A1, followed by oxidation at C-3 by HSD3B7, followed by oxidation at C-26 by CYP27A1 (Fig. 3). Meaney et al, also studying metabolism of deuterium-labelled cholesterol have suggested a similar pathway to be operative in man [26]. The origin of 3β,7β-diHCA is uncertain. The 7β-hydroxy group may be introduced by reduction of a 7-oxo group by HSD11B1 [[63], [64], [65]], which may be derived by non-enzymatic oxidation of cholesterol at C-7 or enzymatic oxidation of 7-DHC by CYP7A1, with CYP27A1 using either 7-oxo or 7β-hydroxycholesterol as a substrate to generate 3β,7β-diHCA (Supplemental Fig. S5). Alternatively, 7β-HC may be introduced by non-enzymatic oxidation of cholesterol. Again, the high degree of enrichment of deuterium in 3β,7β-diHCA suggests CYP27A1 is not involved in a rate-determining step. In human, Shoda et al have suggested that 7α-hydroxy-5-ene sterols can be converted to their 7β-hydroxy equivalents via a mitochondrial enzyme in liver [66], while the intestinal flora may prove an alternative route to 7α/7β-epimerisation [67].

7αH,3O-CA is found as both 25R and 25S epimers in human and mouse [[68], [69], [70]], the 25S-epimer is formed by action of the α-methyl acyl-CoA racemase (AMACR) enzyme on the 25R-CoA thioester in the peroxisome (Fig. 3) [71], in plasma this is usually the minor epimer. There is only a minor (about 5%) difference in the degree of enrichment of deuterium in the two epimers.

#### 3.1.3 Dihydroxyoxocholestenoic acids

**Fig. 5:**
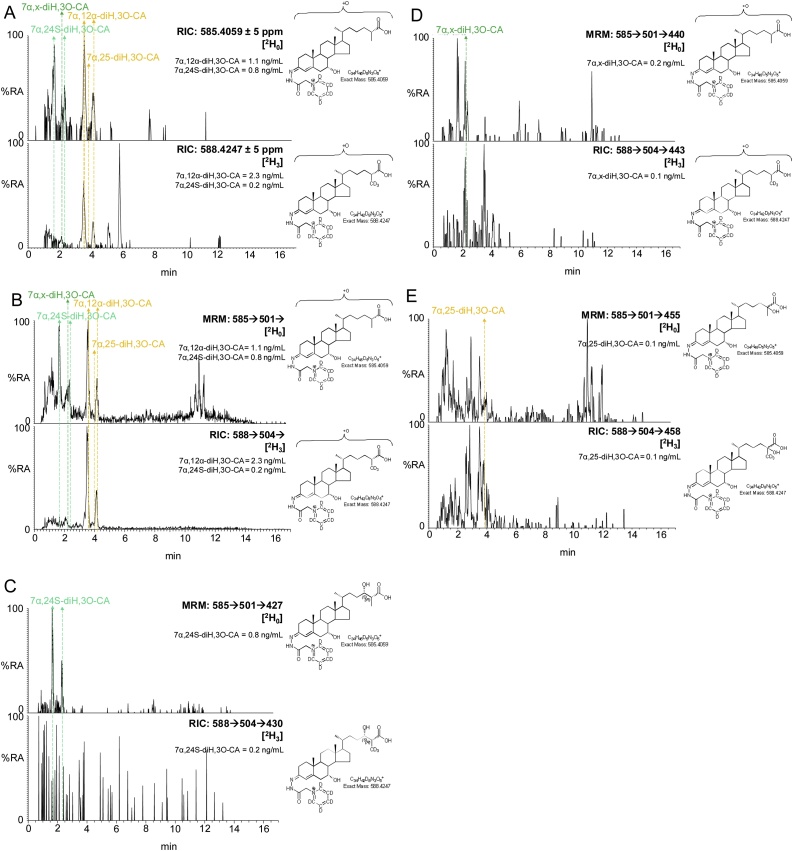
The degree of enrichment of deuterium in 7α,24S-diH,3O-CA is less than in 7α,12α-diH,3O-CA. (A) RICs for the [M]^+^ ions of [^2^H_0_] (*m/z* 585.4059, upper panel) and [^2^H_3_] (*m/z* 588.4247, lower panel) dihydroxy-3-oxocholest-4-en-26-oic acids (diH,3O-CA). (B) TICs for the [M]^+^→ [M-Py]^+^→ transitions for [^2^H_0_] (585.4→501.3→, upper panel) and [^2^H_3_] (588.4→504.4→) appropriate to diH,3O-CA. (C) MRM transitions [M]^+^→ [M-Py]^+^→[M-Py-CO-CO_2_H_2_] for [^2^H_0_] (585.4→501.3→427.3, upper panel) and [^2^H_3_] (588.4→504.4→430.4, lower panel) isotopologues targeting diastereoisomers of 7αH,24-diH,3O-CA [72]. (D) MRM transitions [M]^+^→[M-Py]^+^→[M-Py-NHCO-H_2_O]^+^ for [^2^H_0_] (585.4→501.3→440.3, upper panel) and [^2^H_3_] (588.4→504.4→443.3, lower panel) isotopologues targeting diastereoisomers of 7α,x-diH,3O-CA. (E) MRM transitions [M]^+^→[M-Py]^+^→[M-Py-H_2_CO_2_]^+^ for [^2^H_0_] (585.4→501.3→455.3, upper panel) and [^2^H_3_] (588.4→504.4→458.3, lower panel) isotopologues targeting diastereoisomers of 7αH,25-diH,3O-CA. Note the targeting transitions are not unique to the targeted species. MS^3^ spectra are shown in Supplemental Fig. S6.

In the analysis of mouse plasma two distinct dihydroxyoxocholestenoic acids are evident (Fig. 1, Fig. 5), but with quite different degrees of enrichment of deuterium. The identity of these two acids was not immediately obvious. The latter eluting acid was more abundant and deuterium enrichment was about 70%. This acid was previously observed to be abundant in plasma of the *Amacr* knock-out (-/-) mouse [69], suggesting that it is the 25R epimer and that the two hydroxy groups are at 7α and 12α on a 3-oxocholest-4-en-(25R)26-oic acid skeleton i.e. an intermediate in the biosynthesis of cholic acid (Fig. 3). The MS^3^ spectra recorded of the non-deuterated and deuterated acids were entirely compatible with this assignment (Supplemental Fig. S6C, see also Supplemental Text) [72]. The degree of enrichment of deuterium of 7α,12α-dihydroxy-3-oxocholest-4-en-26-oic acid (7α,12α-diH,3O-CA) was similar to that of 7αH,3O-CA, but different to that of 3β-HCA, indicating that 7α,12α-diH,3O-CA is formed in an extension to the pathway to 7αH,3O-CA, where CYP27A1 does not participate in the rate-determining step. CYP8B1 is the sterol 12α-hydroxylase which will hydroxylate 7α-hydroxysterols and is involved in the biosynthesis of cholic acid [50].

The earlier eluting dihydroxyoxocholestenoic acid was enriched with deuterium to a much lesser extent than 7α,12α-diH,3O-CA at only 20% cf. 70% (Fig. 1, Fig. 5). This suggests that it is formed from a different pool of cholesterol than the other 7α-hydroxy acids, or alternatively, CYP27A1 participates in the rate-determining step and the deuterium isotope effect accounts for the low-degree of enrichment of deuterium. Side-chain shortening of cholestenoic and cholestanoic acids proceeds via thioesterification with CoA, then 24-hydroxylation, further 24-oxidation and ultimately beta-oxidation of the acyl-CoA thioester (Fig. 3). Thioesterification by bile acid CoA synthetase (BACS, *Slc27a5*) or by very long chain fatty-acyl-CoA ligase (or synthetase, VLCS, *Slc27a2*) proceeds in the peroxisome or mitochondria, then in the peroxisome the (25R)acyl-CoA thioester is first converted to its 25S epimer by AMARC, then oxidised by acyl-CoA oxidase 2 (ACOX2) to give a 24-unsaturated thioester which is then 24-hydroxylated followed by 24-dehydrogenation via the enzymes of D-bifunctional protein (DBP), or in its absence, via L-bifunctional protein (LBP) [69,73]. With EADSA methodology we tend to observe the free acid rather than the acyl-CoA thioester. Alternatively, the oxysterol 24S-HC may provide the source of the 24-hydroxy group for side-chain shortening (Fig. 3) [69]. In which case, if the early eluting dihydroxyoxocholestenoic acid is 7α,24S-dihydroxy-3-oxocholest-4-en-(25R)26-oic acid (7α,24S-diH,3O-CA(25R)) the degree of enrichment of deuterium is likely to be similar to that of 24S-HC, where enrichment of deuterium is much less than for 7α-hydroxysterols on account of 24S-HC being derived from non-exchangeable pool of cholesterol isolated behind the BBB.

**Fig. 6:**
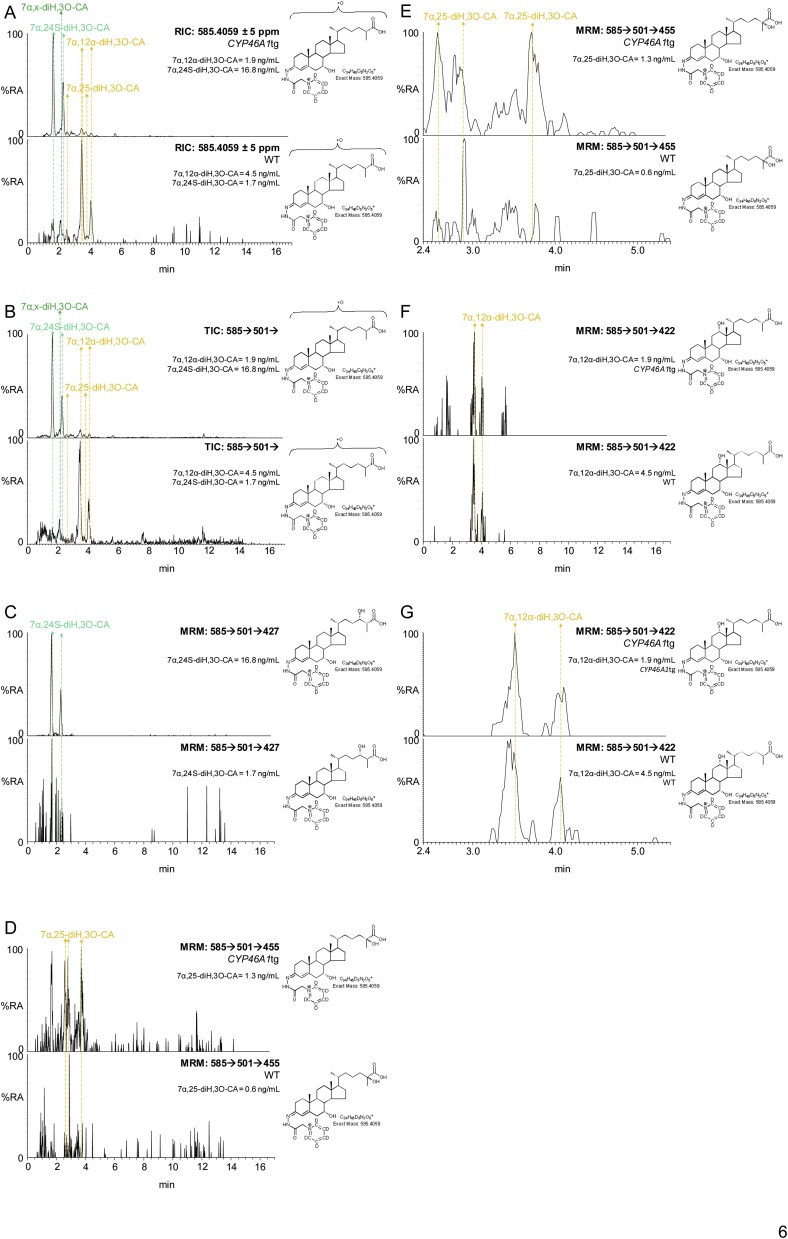
Dihydroxyoxocholestenoic acids in plasma of the *CYP46A1*tg mouse. (A) RICs for the [M]^+^ ions, *m/z* 585.4059, of dihydroxy-3-oxocholest-4-en-26-oic acids (diH,3O-CA) from a *CYP46A1*tg (upper panel) and WT (lower panel) mouse. (B) TICs for the [M]^+^→[M-Py]^+^→ transitions, 585.4→501.3→, appropriate to diH,3O-CA from a *CYP46A1*tg (upper panel) and WT (lower panel) mouse. (C) MRM transitions [M]^+^→[M-Py]^+^→[M-Py-CO-CO_2_H_2_], 585.4→501.3→427.3, targeting diastereoisomers of 7α,24-diH,3O-CA, from a *CYP46A1*tg (upper panel) and WT (lower panel) mouse. (D) MRM transitions [M]^+^→[M-Py]^+^→[M-Py-H_2_CO_2_]^+^, 585.4→501.3→455.3, targeting diastereoisomers of 7α,25-diH,3O-CA, from a *CYP46A1*tg (upper panel) and WT (lower panel) mouse. (E) As in (D) with a retention-time window 2.4–5.4 min. (F) MRM transitions [M]^+^→[M-Py]^+^ →[M-Py-CONH-(H_2_O)_2_]^+^, 585.4→501.3→422.3, targeting 7α,12α-diH,3O-CA, from a *CYP46A1*tg (upper panel) and WT (lower panel) mouse. (G) As in (F) with a retention-time window 2.4–5.4 min. Note the targeting transitions are not unique to the targeted species. The chromatograms shown on the expanded scale have been smoothed. See Supplemental Fig. S7 for fragmentation patterns of dihydroxy-3-oxocholest-4-en-26-oic acids. MS^3^ spectra are shown in Supplemental Fig. S8.

To confirm or not the identity of the lightly deuterium enriched early eluting dihydroxyoxocholestenoic acid as 7α,24S-diH,3O-CA(25R) we analysed plasma from the *CYP46A1*tg mouse over expressing human CYP46A1 (see section 3.2 below). As is evident from Fig. 6, a chromatographic peak is very prominent in plasma from the *CYP46A1*tg mouse with a similar retention time to the early eluting dihydroxyoxocholestenoic acid in Fig. 5. Identification of this acid as 7α,24-diH,3O-CA was made via comparison of retention time and MS^3^ spectrum with the authentic standard prepared by cholesterol oxidase treatment of 3β,7α,24S-trihydroxycholest-5-en-(25R)26-oic acid, recently custom synthesised by Avanti Polar Lipids. However, as is evident from Fig. 3, 7α,24-diH,3O-CA can exist as 24R,25R, 24S,25S and 24S,25R diastereoisomers and in the absence of authentic standards for each compound we cannot be sure which isomer is present in plasma. Our data, however, showing a degree of enrichment of deuterium of only 20% does suggest that the majority of the acid is derived from brain and is thus the 7α,24S-diH,3O-CA(25R) diastereomer. This is supported by the abundant co-eluting peak in the chromatogram from plasma of the *CYP46A1*tg mouse where the CYP46A1 protein is abundant in brain [53].

There are two other minor dihydroxyoxocholestenoic acids evident in plasma, one of which eluting at 2.13 min (Fig. 5) is the most abundant dihydroxyoxocholestenoic acid found in CSF [72] and annotated, based on its MS^3^ spectrum, and in the absence of authentic standards, as 7α,x-dihydroxy-3-oxocholest-4-en-26-oic acid (7α,x-diH,3O-CA), where x is probably 22, 23 or perhaps 27. Triple deuteration rules out the possibility of the additional hydroxyl group on C-27. The second minor dihydroxyoxocholestenoic elutes at 3.8 min and corresponds to one of the diasterioisomers of 7α,25-dihydroxy-3-oxocholest-4-en-26-oic acid [72] (7α,25-diH,3O-CA). The degree of enrichment of deuterium for these two minor components was found to be 40%–60% indicating that they are not formed behind the BBB.

As illustrated in Fig. 3, the pathways starting with 7α-, (25R)26- and 24S-hydroxylation of cholesterol converge at 7α-hydroxy-3,24-*bis*oxocholest-4-en-(25R)26-oic acid (7αH,3,24-diO-CA) [71,73]. 7αH,3,24-diO-CA is unstable and will decarboxylate to 7α-hydroxy-26-*nor*cholest-4-ene-3,24-dione (7αH,26-nor-C-3,24-diO) [19,74]. The degree of enrichment of deuterium of 7αH,26-nor-C-3,24-diO was found to be 50%, a value intermediate between that for 7α,24S-diH,3O-CA(25R), i.e. 20%, and the 25R and 25S epimers of 7αH,3O-CA, i.e. 60–65%. This is entirely consistent with the notion that the cholestenoic acid content of plasma reflects the different pathways of bile acid biosynthesis, both hepatic and non-hepatic [41].

### 3.2 Oxysterols and cholestenoic acids in the CYP46A1tg mouse

In the WT mouse, both 24S-HC and 24R-HC epimers are present in plasma, the 24S-epimer being about five times more abundant than the R-epimer, when measured after saponification of oxysterol esters [28]. As discussed above, in mouse 24S-HC observed in the circulation is formed predominantly in brain but also to a minor extent extra-cerebrally, while 24R-HC is formed extra-cerebrally but is also present at low levels in brain, perhaps originating from the circulation [[75], [76], [77]]. In human, 24R-HC is barely detectable in the circulation and has not yet been reported to be present in brain where 24S-HC is the dominant oxysterol [78].

In the *CYP46A1*tg mouse, human *CYP46A1* is expressed under control of the β-actin promoter [53]. The protein is found mostly in brain, but also to a minor extent (10% cf. brain 100%) in eye and testis, two other organs separated from the circulation by barriers akin to the BBB [53]. In the WT mouse CYP46A1 protein is found in testis but not eye [53]. The *CYP46A1*tg mouse provides an excellent model to define cholesterol metabolites synthesised behind these barriers, predominantly from brain, adding further insight to the mechanistic proposals made above.

We have previously analysed the 24R/S-HC content of plasma from the *CYP46A1*tg mouse after base hydrolysis of oxysterol esters [28]. We found the concentration of 24S-HC to be almost doubled compared to the WT mouse but that of 24R-HC to be increase by only one third. This indicates a higher flux of 24S-HC into the circulation from the mutant mouse, although part of the increase in 24S-HC and 24R-HC may be from extracerebral tissue [28]. We have now extended this earlier study to investigate non-esterified oxysterols (measured in the absence of base hydrolysis).

#### 3.2.1 Oxysterols

**Fig. 7:**
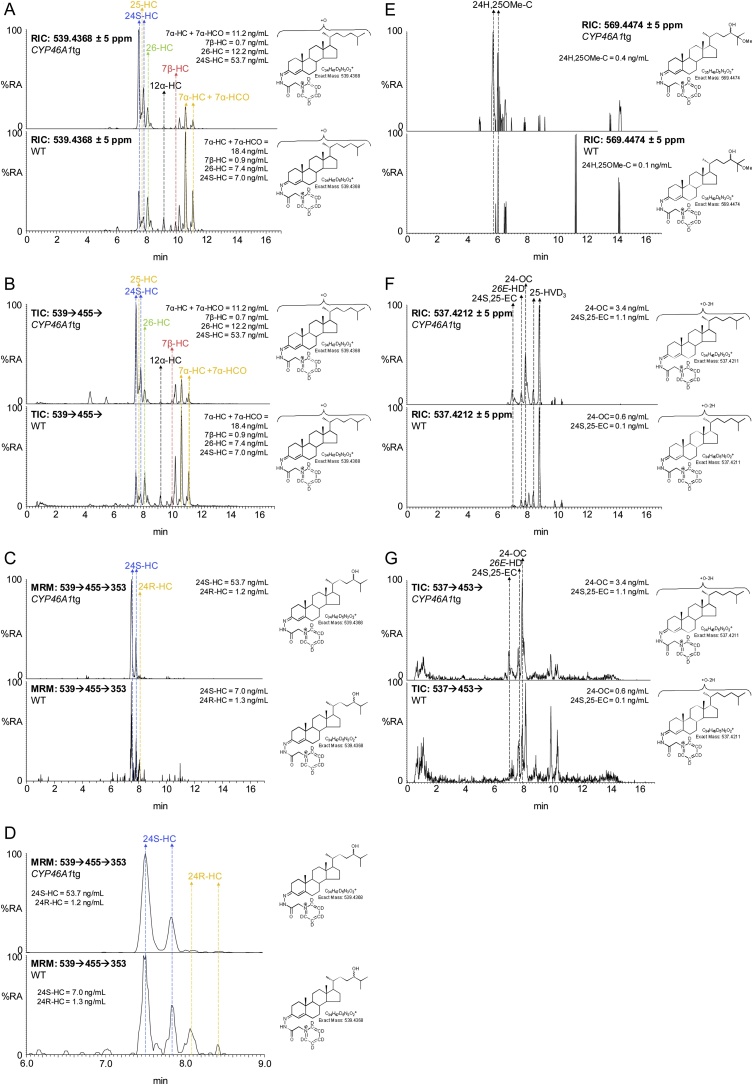
24S-HC and 24S,25-EC are elevated in plasma from a *CYP46A1*tg mouse. (A) RICs for the [M]^+^ ions of monohydroxycholesterols (*m/z* 539.4368) from a *CYP46A1*tg mouse (upper panel) and a WT mouse (lower panel). (B) TICs for the [M]^+^→[M-Py]^+^→ transitions for monohydroxycholesterols from the *CYP46A1*tg mouse (upper panel) and a WT mouse (lower panel). (C) MRM transitions [M]^+^→[M-Py]^+^→353.3 targeting 24-HC epimers (see Supplemental Scheme S3) from a *CYP46A1*tg mouse (upper panel) and a WT mouse (lower panel). (D) As in (C) displaying the retention-time window 6–9 min. (E) RICs for the [M]^+^ ions of 24H,25OMe-C (*m/z* 569.4474) from the *CYP46A1*tg mouse (upper panel) and a control mouse (lower panel). (F) RICs for the [M]^+^ ions of *m/z* 537.4212 corresponding to 24S,25-EC and isomers thereof from the *CYP46A1*tg mouse (upper panel) and a control mouse (lower panel). (G) TICs for the [M]^+^→[M-Py]^+^→ transitions for 24S,25-EC, 24-OC and 26-HD from the *CYP46A1*tg mouse (upper panel) and a WT mouse (lower panel). MS^3^ spectra are shown in Supplemental Fig. S9. Shown in Supplemental Fig. S10 is a scheme depicting isomerisation, hydrolysis and methanolysis of 24S, 25-EC.

A limitation of the current study is that plasma from only two transgenic animals was analysed, hence the data should be regarded as “proof of principle”. Despite this limitation, the concentration of 24S-HC was found to be elevated by factors of three and seven in the two *CYP46A1*tg mice over the wild-type mouse with the highest concentration of 24S-HC (Fig. 7, Supplemental Table S3). In the two *CYP46A1*tg mice the 24R-HC epimer contributed 2–3% of the total 24-HC concentration; in comparison in the three WT mice the 24R-HC epimer contributed 15–20% to the total 24-HC. This data agrees with that obtained by Saeed et al for 24-HC following saponification of oxysterol esters, confirms that the majority of 24S-HC is from cerebral tissue and suggests that most 24R-HC is generated by an enzyme other than CYP46A1 [28]. Importantly, Meljon et al have shown that 24R-HC is present in brain of the *Cyp46a1-/-* mouse adding weight to the suggestion that its biosynthesis is independent of CYP46A1 [75].

**Fig. 8:**
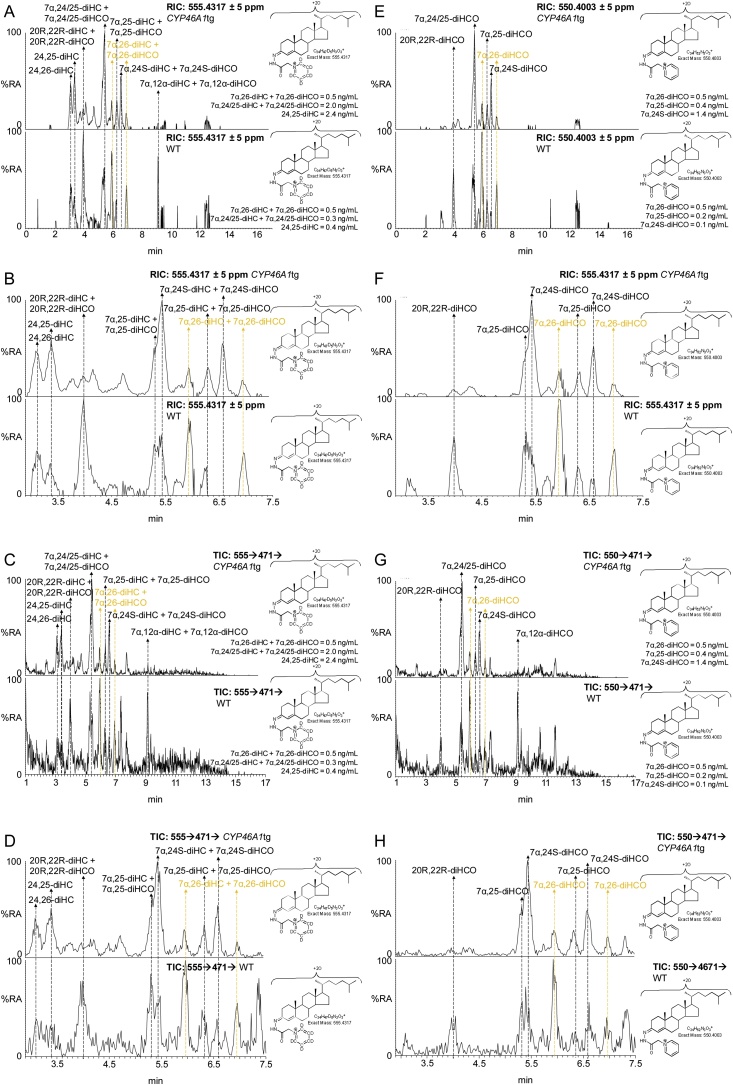
7α,24S-diHCO is elevated in *CYP46A1*tg mouse plasma. (A) RICs for the [M]^+^ ions of dihydroxycholesterols and dihydroxycholestenones (*m/z* 555.4317) from the *CYP46A1*tg mouse (upper panel) and a WT mouse (lower panel). (B) As in (A) displaying the retention-time window 2.9–7.5 min. (C) TICs for the [M]^+^→ [M-Py]^+^→ transitions for dihydroxycholesterols and dihydroxycholestenones from the *CYP46A1*tg mouse (upper panel) and a WT mouse (lower panel). (D) As in (C) displaying the retention-time window 2.9–7.5 min. Data in (A) – (D) from Fraction A prepared with cholesterol oxidase. (E) RICs for the [M]^+^ ions of dihydroxycholestenones (*m/z* 550.4003) from the *CYP46A1*tg mouse (upper panel) and a WT mouse (lower panel). (F) As in (E) displaying the retention-time window 2.9–7.5 min. (G) TICs for the [M]^+^→ [M-Py]^+^→ transitions for dihydroxycholestenones from the *CYP46A1*tg mouse (upper panel) and a WT mouse (lower panel). (H) As in (G) displaying the retention-time window 2.9–7.5 min. Data (E) – (H) from Fraction B prepared in the absence of cholesterol oxidase. MS^3^ spectra are shown in Supplemental Fig. S11.

Of the other oxysterols, the concentration of 24S,25-EC was found to be increased in the *CYP46A1*tg mice. Using EADSA technology 24S,25-EC is unstable being hydrolysed to 24,25-dihydroxycholesterol (24,25-diHC), undergoing methanolysis to 24-hydroxy,25-methoxycholesterol (24H,25OMe-C), both in acid catalysed reactions with solvent, and isomerisation to 24-oxocholesterol (24-OC). Taking total 24S,25-EC to be the sum of remaining unreacted 24S,25-EC and these three products, the plasma concentrations of total 24S,25-EC were factors of two and four greater in the *CYP46A1*tg mice than in the WT mouse with highest 24,25-EC concentration (Supplementary Table 3). We have previously reported a similar elevation in 24S,25-EC in brain of *CYP46A1*tg embryos and adults [79]. When broken down into the component elements unmodified 24S,25-EC increased by factors of about five and ten, 24-OC by factors of about two and three, and the hydrolysis products by factors of about two and four over the WT mice with highest concentration of these molecules (Fig. 7, Fig. 8). The concentration of the methanolysis product increased in only one of the mice, by a factor of 1.3 over the WT mouse with highest concentration of 24H,25OMe-C. It should be noted 24,25-diHC could be formed also by CYP46A1 oxidation of cholesterol [80]. The likely metabolic route of 24S,25-EC is 7α-hydroxylation by CYP7B1, as the plasma concentration of 24S,25-EC is elevated in the *CYP7b1-/-* mouse [76].

CYP39A1 is the dominant 24S-hydroxycholesterol 7α-hydroxylase [81]. 7α,24S-Dihydroxycholesterol (7α,24S-diHC) is metabolised further by HSD3B7 to give 7α,24S-dihydroxycholest-4-en-3-one (7α,24S-diHCO). In our chromatographic system the first peaks of 7α,24S-dihydroxysterols almost co-elute with their 7α,25-dihydroxy-isomers but the less intense second peaks of the *syn* and *anti* conformers are resolved, allowing deconvolution of relative abundance (Fig. 8). Peaks corresponding to 7α,24S-diHCO and 7α,25-diHCO are observed in both genotypes. The peaks corresponding to 7α,24S-diHCO were elevated in the two transgenic mice by factors of about ten above the levels in the WT animals, while there was little difference in 7α,25-diHCO or 7α,26-diHCO concentrations in the two genotypes. The levels of the dihydroxycholesterols were near or below the limit of detection of our analytical method.

Of the other oxysterols analysed in this study, the concentrations of 25-HC and 26-HC increased in only one of the *CYP46A1*tg animals, while the concentrations of 7α-HC, 7β-HC, 7-OC and 6-hydroxycholesterol (6-HC) did not differ between genotypes. 7β-HC, 7-OC and 6-HC, which is a dehydration product of cholestane-3β,5α,6β-triol formed from 5,6-EC by cholesterol epoxide hydrolase [82], are derived through both enzymatic and non-enzymatic reactions. Interestingly, the concentrations of 7α-HCO in the *CYP46A1*tg mice were about half those in the WT mouse with the lowest plasma concentration. 7α-HCO is a plasma marker for CYP7A1 activity, and its reduced level in the *CYP46A1*tg mouse is consistent with reduced expression of *Cyp7a1* in liver of this mouse observed by Shafaati et al [53].

With respect to the oxysterols only partially identified and annotated as 3β,x-diHC-yO, x-HC-3,y-diO and 3β,x,y-triHC-zO, there is no increase in plasma concentration in the *CYP46A1*tg mouse indicating that side-chain hydroxylation is not through CYP46A1.

#### 3.2.2 Cholestenoic acids

We discussed above how plasma from the *CYP46A1*tg mouse was used to identify 7α,24S-diH,3O-CA in the mouse fed deuterated cholesterol. This acid is metabolised further to the CoA-thioester of 7αH,3,24-diO-CA (Fig. 3) which under our analytical system is unstable and decomposes by decarboxylation to 7αH-26-nor-C-3,24-diO. In the WT mouse this is just a minor metabolite but is elevated by a factor of about two and six in the two transgenic animals.

Interestingly, the abundance of the acid identified as 7α,12α-diH,3O-CA was reduced in the transgenic animals. This agrees with the pathway which proceeds through 7α-HC and 7α-HCO and the data from Shafaati et al indicating the expression in liver of *Cyp7a1* is reduced in the *CYP46A1*tg mouse [53]. The plasma concentration of 7α,x-diH,3O-CA, where x is probably 22, 23 or perhaps 27, is reduced in the *CYP46A1*tg arguing against an involvement of CYP46A1 in side-chain hydroxylation.

Of the other cholestenoic acids 7α,25-diH,3O-CA, 3β,7α-diHCA, 7αH,3O-CA and 3β-HCA there were no obvious differences in plasma concentrations between the transgenic and WT animals.

## 4 Discussion

In the current study we have utilised two mouse models to investigate the origins of oxysterols and cholestenoic acids found in the circulation. Our intention in this study was to identify minor metabolic pathways, without evaluating their importance. Higher numbers of animals will be required to evaluate the significance of the metabolic pathways uncovered. With the first mouse model, *pdgfb*^ret/ret^, we investigated the degree of enrichment of deuterium of plasma sterols after 40 days of treatment of a single male animal with [26,26,26,27,27,27-^2^H_6_] cholesterol. After 40 days the amount of deuterated cholesterol was 70% of the total cholesterol in this mouse [28]. The *pdgfb*^ret/ret^ mouse is a pericyte-deficient mouse model presenting with enhanced BBB permeability. The advantage of this model is that it expedites the measurement of brain derived cholesterol metabolites in plasma. A disadvantage is the import of a small amount of cholesterol from the circulation into brain will result in some increased deuterium content of brain-derived cholesterol metabolites [28]. In fact, in our earlier study [28], the incorporation of deuterium in brain cholesterol after 40 days of feeding of the mouse was only about 7%, corresponding to replacement of about 10% of brain cholesterol with plasma cholesterol under the conditions used. A second disadvantage of this model is that there is increased vascular permeability not only in the brain but also in the liver. The lipoprotein pattern in the circulation is abnormal with low levels of HDL [28]. The use of the second mouse model, *CYP46A1*tg, where human *CYP46A1* is expressed under control of the β-actin promoter with high CYP46A1 protein expression in brain [53], allowed the definition of oxysterols derived from CYP46A1 activity. A limitation of this study is that only two *CYP46A1*tg animals were investigated, however, this did not obscure obvious differences in the oxysterol content of plasma between *CYP46A1*tg and WT mice.

As expected, the degree of enrichment in deuterium of 24S-HC found in plasma was much lower than of any other oxysterol. This agrees with earlier studies investigating 24S-HC plasma concentrations following saponification of oxysterol esters [27,28]. The degree of enrichment of deuterium in our study was about 10%. When the degree of enrichment of deuterium of plasma cholesterol of 70% is considered a corrected value of about 15% is obtained. This low degree of enrichment of deuterium compared to corrected values of about 100% for liver derived 7α-HC and 7α-HCO is explained by the biosynthesis of 24S-HC from a pool of cholesterol isolated from the circulation by the BBB. The degree of enrichment of deuterium of 15% is artificially high on account of some leakage over the pericyte deficient BBB of deuterated cholesterol, estimated to be about 10% in the earlier study of Saeed et al [28]. The remaining source of deuterated 24S-HC is likely to be extra-cerebral [27]. CYP46A1 is known to be the dominant cholesterol 24S-hydroxylase [34]. This is confirmed here by experiments with the *CYP46A1*tg mouse where the plasma content of 24S-HC increased by a factor of at least three over the WT. The concentration of 24R-HC did not differ between genotypes.

The *CYP46A1*tg mouse model provided more information on the origin of 24S,25-EC, where the plasma content was more than double that in the WT mouse. This can be interpreted by either its direct biosynthesis from desmosterol via CYP46A1 [36] or via the shunt pathway [37], up-regulated as a consequence of increased cholesterol metabolism by CYP46A1 in brain.

Cholestenoic acids are acidic oxysterols formed by the further CYP27A1 oxidation of a terminal primary alcohol on the sterol side-chain to a carboxylic acid. Their synthesis from [26,26,26,27,27,27-^2^H_6_]cholesterol is attenuated as a consequence of the H/D-isotope effect, a result of the enhanced strength of the C–D bond. The H/D-isotope effect is also evident in 26-HC where the degree of enrichment of deuterium was measured to be about 50% and corrected to about 70% if circulating cholesterol was 100% deuterated. These values are exaggerated for 3β-HCA where three deuterium atoms are removed from [^2^H_6_]cholesterol, giving values at about 30% and corrected to 40% if cholesterol were fully deuterated. Informatively, the degree of enrichment of deuterium of 7α,26-diHCO, 7αH,3O-CA and 7α,12α-diH,3O-CA is much higher with corrected values of about 95–100%, more in line with values for liver derived 7α-HC and 7α-HCO. This indicates that 7α,26-diHCO, 7αH,3O-CA and 7α,12α-diH,3O-CA must be formed from a different pool of cholesterol than 26-HC and 3β-HCA and/or their mechanism of formation does not involve the removal of deuterium in the rate determining step (Fig. 3) [83]. This data for mouse is in good general agreement to that found when a similar deuterium enrichment strategy was employed in man [26]. Meaney et al suggested that in man, liver derived 7α-HC provides the substrate to generate 7α-HCO and subsequently 7αH,3O-CA, while pulmonary derived 3β-HCA provides the substrate to generate 3β,7α-diHC. 26-HC was interpreted to be derived from a third pool of cholesterol [26]. A difference between mouse and man is that 3β,7α-diHCA is essentially absent from mouse plasma, its likely metabolic route being to 7αH,3O-CA accounting for the small decrease in deuterium content of 7αH,3O-CA compared to 7α-HC, 7α-HCO and 7α,26-diHCO.

24S-HC is metabolised by CYP39A1 to 7α,24S-diHC and then to 7α,24S-diHCO. *Cyp39a1* is broadly expressed, including expression in brain and liver, while *Hsd3b7*, the gene encoding 3β-hydroxy-Δ^5^-steroid oxidoreductase is ubiquitously expressed [84]. The concentration of 7α,24S-diHCO is elevated in plasma of the *CYP46A1*tg mouse as is 7αH-26-nor-C-3,24-diO derived from 7αH,3,24-diO-CA by decarboxylation [74], although the concentration of 7α,24S-diH,3O-CA was elevated in only one of the two *CYP46A1*tg animals. These three metabolites sit in a pathway from 24S-HC to bile acids, 7αH,3,24-diO-CA, activated as the CoA thioester, undergoing side-chain shortening in a reaction catalysed by sterol carrier protein x in the peroxisome (SCPx or sterol carrier protein 2, Fig. 3). 7α,24S-diHCO is of low concentration in plasma of WT mice disallowing measurements of enrichment of deuterium, however, the degree of enrichment of deuterium of 7α,24S-diH,3O-CA was only 20%, corrected to 30% for 100% deuterated cholesterol, suggesting most of its synthesis is from a pool of cholesterol different to 7αH,3O-CA and 7α,12α-diH,3O-CA i.e. a pool behind the BBB in brain. Another source of 7α,24S-diH,3O-CA, independent of 24S-HC, is through the action of ACOX2 and LBP rather than DBP on 7αH,3O-CA and this may account for a proportion of the formation of 7α,24S-diH,3O-CA. The degree of enrichment of deuterium of 7αH-26-nor-C-3,24-diO, derived from 7αH,3,24-diO-CA by decarboxylation, was 50% corrected to 70%, considerably lower than that of 7αH,3O-CA but somewhat higher than 7α,24S-diH,3O-CA, this is perfectly compatible with the multiple metabolic pathways converging at 7αH,3,24-diO-CA during bile acid biosynthesis (Fig. 3).

Besides the oxysterols and cholestenoic acids discussed above several other sterol metabolites were partially identified or identified and not quantified. 20R,22R-Dihydroxycholesterol (20R,22R-diHC) and surprisingly, 20R,22R-dihydroxycholest-4-en-3-one (20R,22R-diHCO), as 20R,22R-diHC is not a substrate for HSD3B7, fall into the latter category, as does 26*E*-hydroxydesmosterol. Relative, rather than absolute quantification was performed on 25-hydroxyvitamin D_3_ in the absence of an isotope-labelled authentic standard, while 3β,x-dihydroxycholest-y-one and x-hydroxycholest-3,y-dione, where x and y are probably 20 and 22, or 22 and 24; 3β,x,y-trihydroxycholest-5-en-z-one where x, y and z are probably 22, 25 and 24 and 7α,x-dihydroxy-3-oxo-cholest-4-en-26-oic acid, were partially identified in the absence of authentic standards.

## 5 Conclusions

There are limitations to the current study. Specifically, only one animal was treated with [^2^H_6_]cholesterol for 40 days and plasma from only two *CYP46A1*tg animals was analysed. It may also be argued that there are shortcomings in using the the *pdgfb*^ret/ret^ mouse model with a leaky BBB resulting in artificially high degrees of enrichment of deuterium of brain derived metabolites found in plasma. The counter argument is that to detect deuterated molecules in plasma, a sufficient amount (pmol/mL) must be available. Never-the-less the results from the experiments with deuterated cholesterol are readily explained based on our current knowledge of 24S-HC metabolism in mouse. The use of only two *CYP46A1*tg animals is not ideal. However, our results are only interpreted where both mice give the same direction of change in terms of metabolite concentration. Admittedly, subtler differences between *CYP46A1*tg and WT animals will be lost.

Despite these reservations, the current study confirmed a pathway from 24S-HC towards bile acids, demonstrated the non-cerebral CYP46A1-indipendent biosynthesis of 24R-HC and supported the existence of different pathways for the formation of 3β-HCA and 7αH,3O-CA.

## Declaration of Competing Interest

PJC, WJG and YW are listed as inventors on the patent “Kit and method for quantitative detection of steroids” US9851368B2, which is licenced to Avanti Polar Lipids Inc and Cayman Chemical Company by Swansea Innovations, a wholly owned subsidiary of Swansea University. The funders had no role in the design of the study; in the collection, analyses, or interpretation of data; in the writing of the manuscript; or in the decision to publish the results.
