# Supplementary material for: Formation and metabolism of oxysterols and cholestenoic acids found in the mouse circulation: Lessons learnt from deuterium-enrichment experiments and the CYP46A1 transgenic mouse
Source: J Steroid Biochem Mol Biol. 2019 Dec;195:105475. doi: 10.1016/j.jsbmb.2019.105475 (PMC6880786; doi:10.1016/j.jsbmb.2019.105475)
Supplement: Supplementary file 1 [file mmc1.pdf]

Figure S1

Figure S1

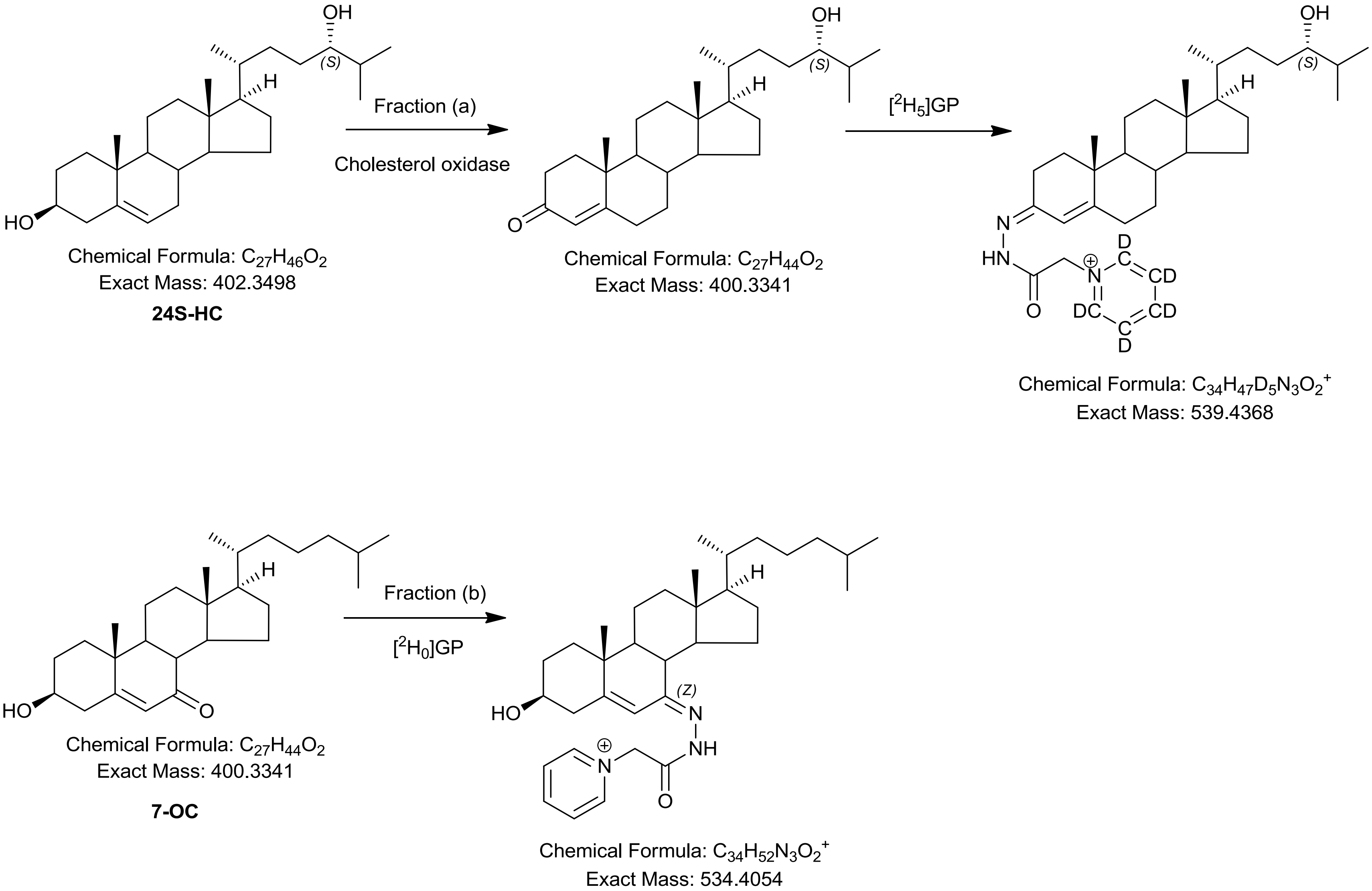

Figure S2A

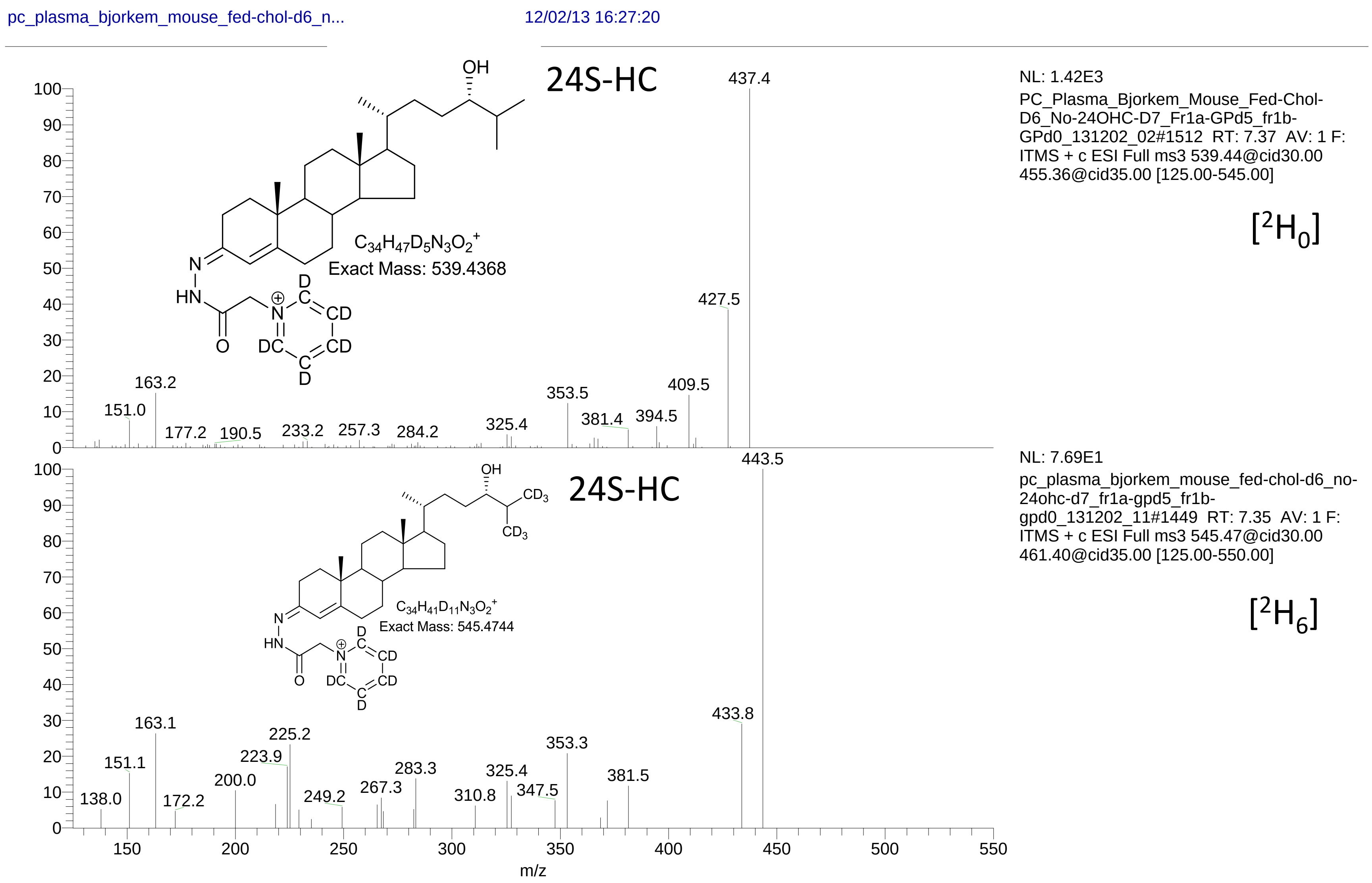

pc_plasma_bjorkem_mouse_fed-chol-d6_n...
12/02/13 16:27:20
24S-HC
437.4
NL: 1.42E3
PC_Plasma_Bjorkem_Mouse_Fed-Chol-D6_No-24OHC-D7_Fr1a-GPd5_fr1b-GPd0_131202_02#1512 RT: 7.37 AV: 1 F: ITMS + c ESI Full ms3 539.44@cid30.00 455.36@cid35.00 [125.00-545.00]
[2H0]
C34H47D5N3O2+
Exact Mass: 539.4368
427.5
163.2
151.0
353.5
409.5
381.4
394.5
325.4
177.2
190.5
233.2
257.3
284.2
443.5
NL: 7.69E1
pc_plasma_bjorkem_mouse_fed-chol-d6_no-24ohc-d7_fr1a-gpd5_fr1b-gpd0_131202_11#1449 RT: 7.35 AV: 1 F: ITMS + c ESI Full ms3 545.47@cid30.00 461.40@cid35.00 [125.00-550.00]
24S-HC
[2H6]
C34H41D11N3O2+
Exact Mass: 545.4744
433.8
163.1
225.2
223.9
353.3
151.1
200.0
283.3
325.4
381.5
347.5
267.3
138.0
172.2
249.2
310.8
m/z

Figure S2B

pc_plasma_bjorkem_mouse_fed-chol-d6_n... 12/02/13 16:27:20

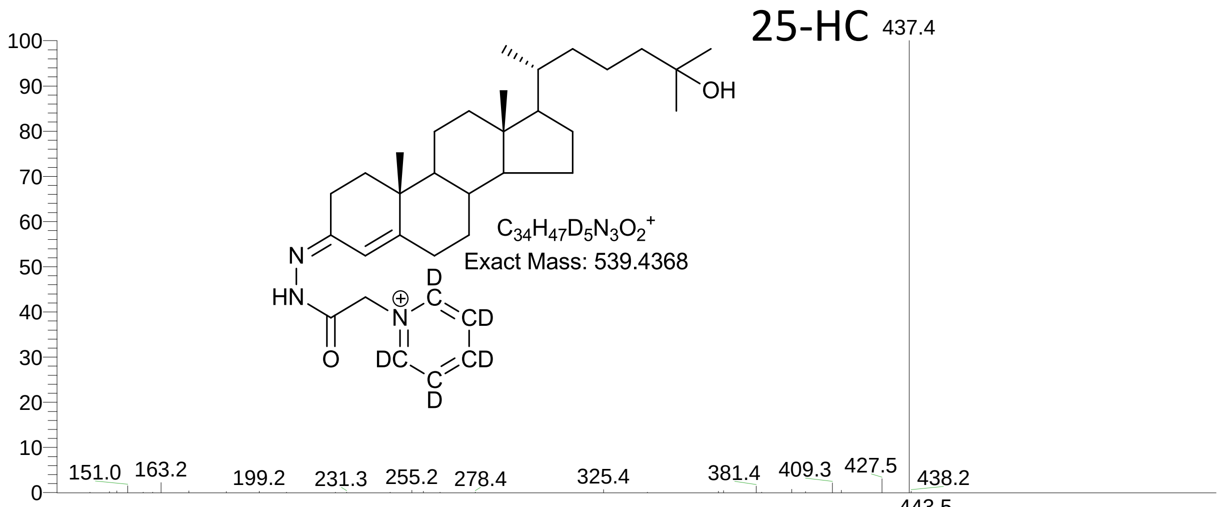

NL: 2.02E3
PC_Plasma_Bjorkem_Mouse_Fed-Chol-D6_No-24OHC-D7_Fr1a-GPd5_fr1b-GPd0_131202_02#1545 RT: 7.53 AV: 1 F: ITMS + c ESI Full ms3 539.44@cid30.00 455.36@cid35.00 [125.00-545.00]

$[^2H_0]$

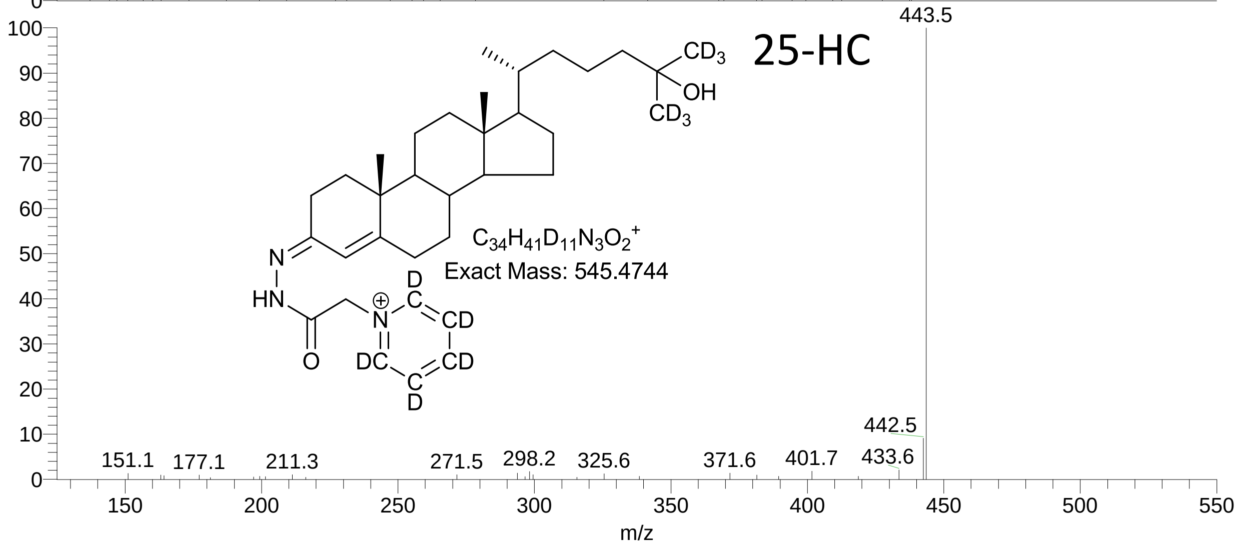

NL: 6.49E2
pc_plasma_bjorkem_mouse_fed-chol-d6_no-24ohc-d7_fr1a-gpd5_fr1b-gpd0_131202_11#1482 RT: 7.52 AV: 1 F: ITMS + c ESI Full ms3 545.47@cid30.00 461.40@cid35.00 [125.00-550.00]

$[^2H_6]$

Figure S2C

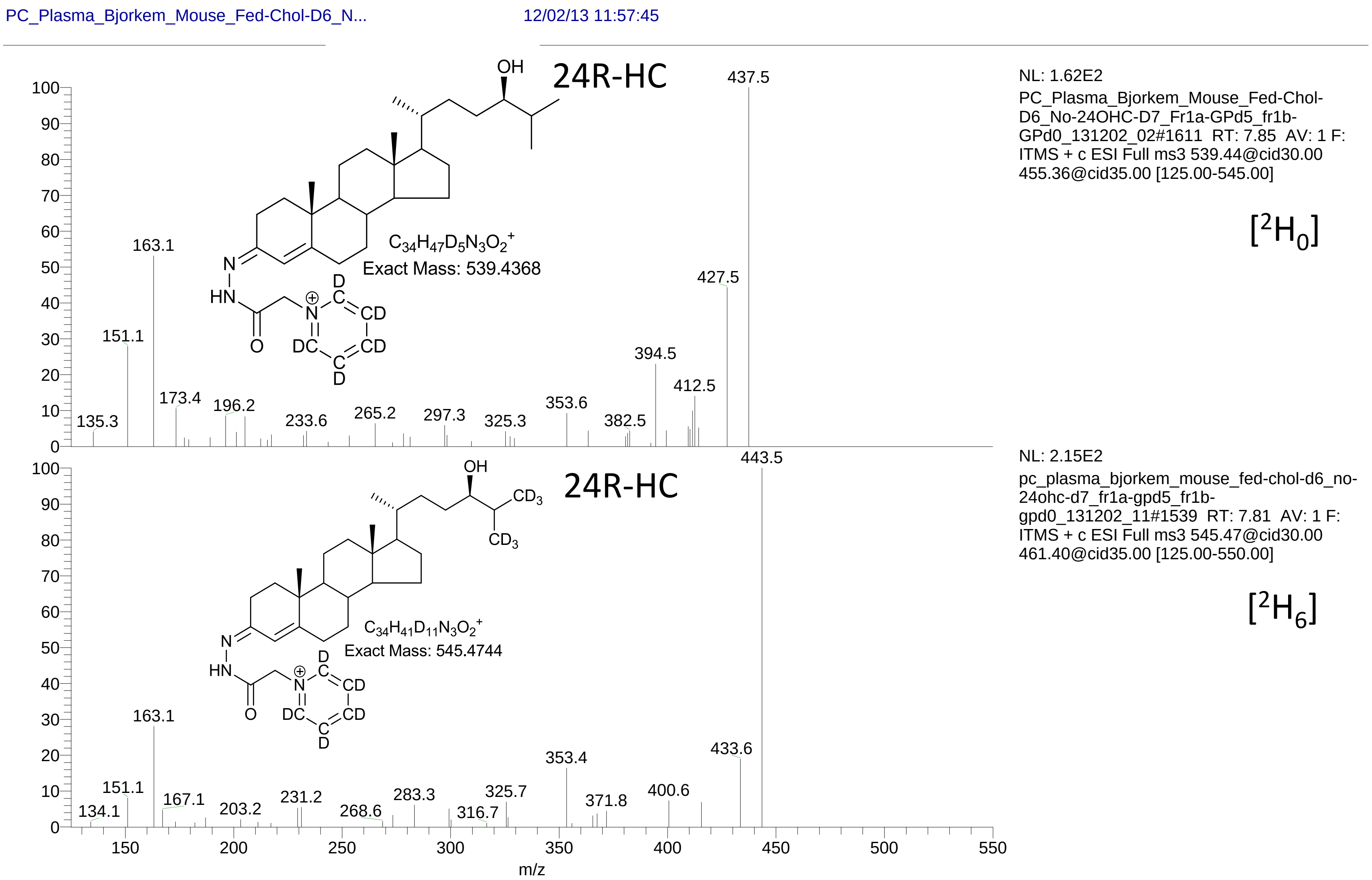

PC_Plasma_Bjorkem_Mouse_Fed-Chol-D6_N...
12/02/13 11:57:45
24R-HC
OH
$C_{34}H_{47}D_5N_3O_2^+$
Exact Mass: 539.4368
NL: 1.62E2
PC_Plasma_Bjorkem_Mouse_Fed-Chol-D6_No-24OHC-D7_Fr1a-GPd5_fr1b-GPd0_131202_02#1611 RT: 7.85 AV: 1 F: ITMS + c ESI Full ms3 539.44@cid30.00 455.36@cid35.00 [125.00-545.00]
[$^2H_0$]
437.5
427.5
394.5
412.5
163.1
151.1
173.4
196.2
135.3
233.6
265.2
297.3
325.3
353.6
382.5
24R-HC
OH
CD_3
$C_{34}H_{41}D_{11}N_3O_2^+$
Exact Mass: 545.4744
NL: 2.15E2
pc_plasma_bjorkem_mouse_fed-chol-d6_no-24ohc-d7_fr1a-gpd5_fr1b-gpd0_131202_11#1539 RT: 7.81 AV: 1 F: ITMS + c ESI Full ms3 545.47@cid30.00 461.40@cid35.00 [125.00-550.00]
[$^2H_6$]
443.5
433.6
353.4
163.1
151.1
134.1
167.1
203.2
231.2
268.6
283.3
316.7
325.7
371.8
400.6
m/z

Figure S2D

pc_plasma_bjorkem_mouse_fed-chol-d6_n... 12/02/13 16:48:04

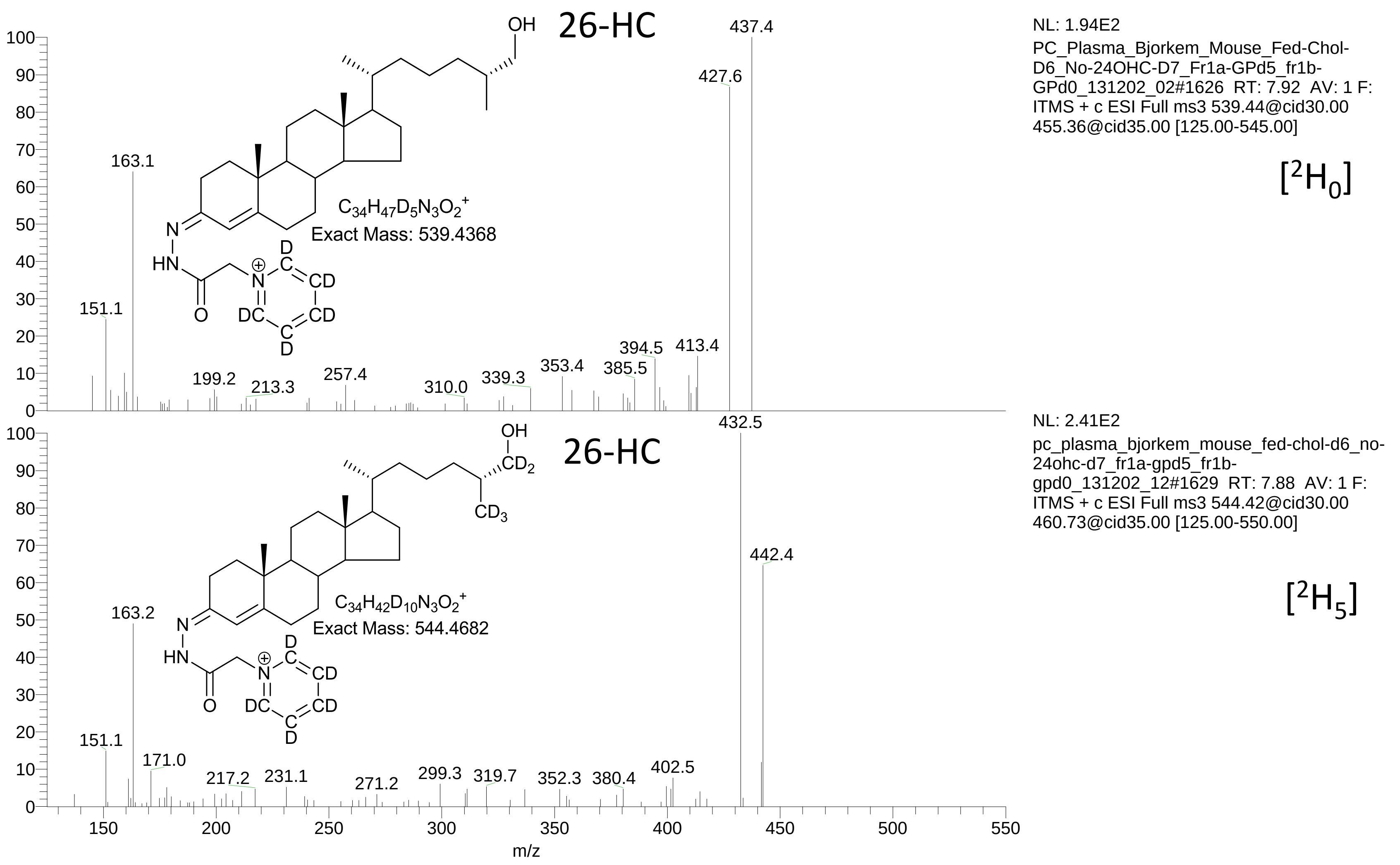

Figure S2E

PC_Plasma_Bjorkem_Mouse_Fed-Chol-D6_N... 12/02/13 11:57:45

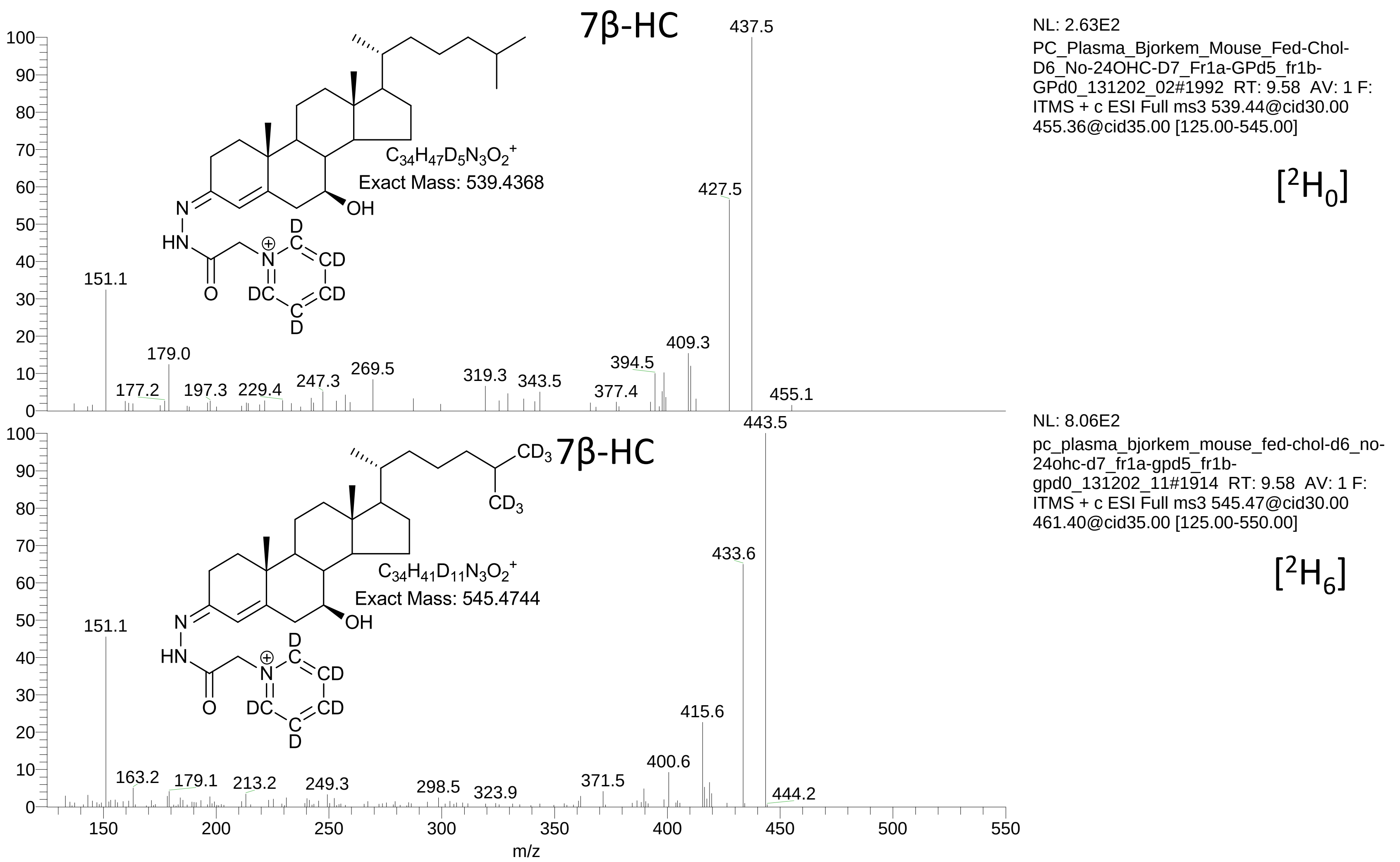

Figure S2F

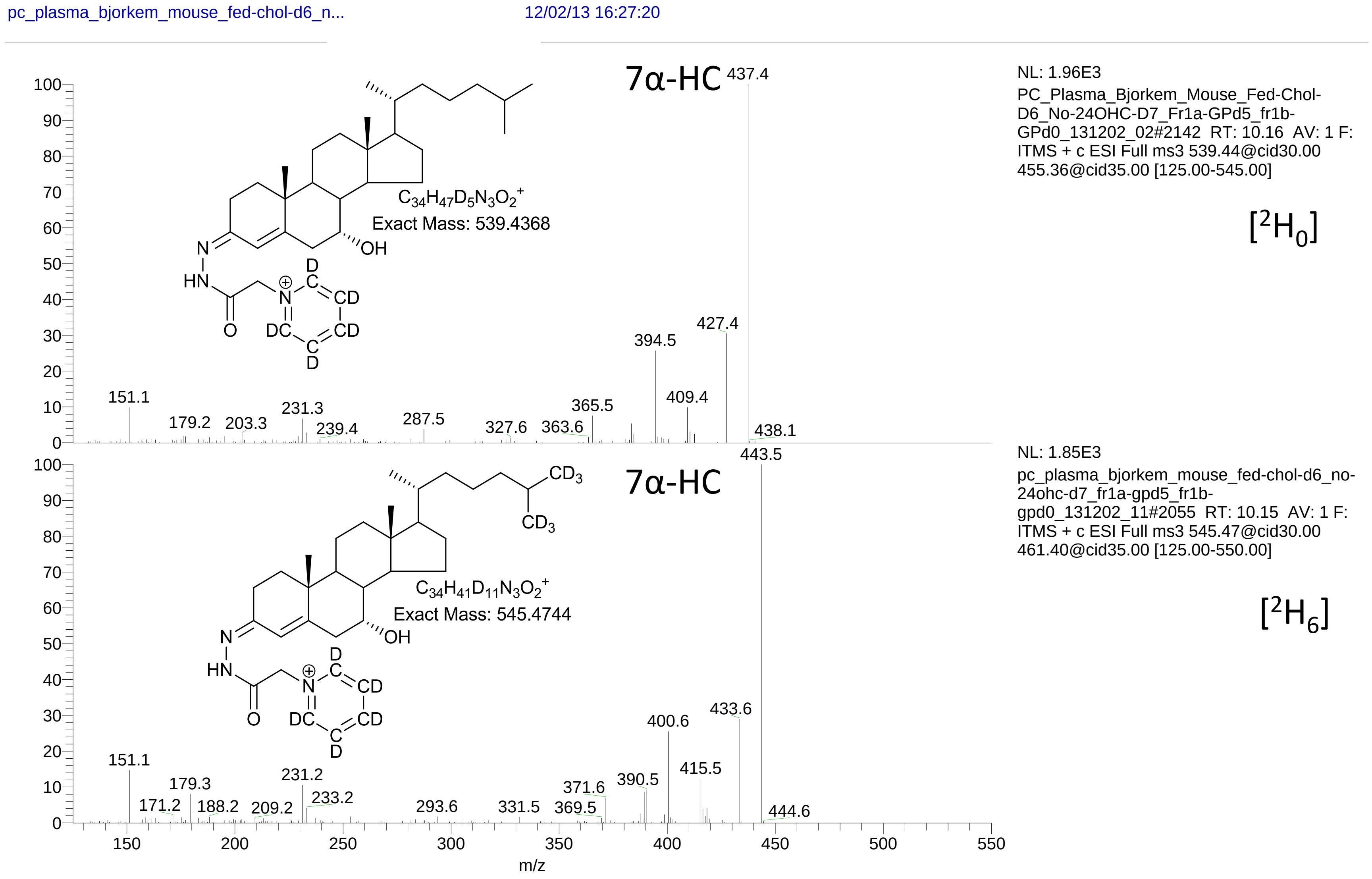

pc_plasma_bjorkem_mouse_fed-chol-d6_n...
12/02/13 16:27:20
7α-HC
$C_{34}H_{47}D_5N_3O_2^+$
Exact Mass: 539.4368
NL: 1.96E3
PC_Plasma_Bjorkem_Mouse_Fed-Chol-D6_No-24OHC-D7_Fr1a-GPd5_fr1b-GPd0_131202_02#2142 RT: 10.16 AV: 1 F: ITMS + c ESI Full ms3 539.44@cid30.00 455.36@cid35.00 [125.00-545.00]
[$^2H_0$]
437.4
427.4
394.5
409.4
365.5
151.1
179.2
203.3
231.3
239.4
287.5
327.6
363.6
438.1
7α-HC
$C_{34}H_{41}D_{11}N_3O_2^+$
Exact Mass: 545.4744
NL: 1.85E3
pc_plasma_bjorkem_mouse_fed-chol-d6_no-24ohc-d7_fr1a-gpd5_fr1b-gpd0_131202_11#2055 RT: 10.15 AV: 1 F: ITMS + c ESI Full ms3 545.47@cid30.00 461.40@cid35.00 [125.00-550.00]
[$^2H_6$]
443.5
433.6
400.6
415.5
390.5
371.6
369.5
331.5
293.6
233.2
231.2
209.2
188.2
179.3
171.2
151.1
444.6
m/z

Figure S3

Figure S3

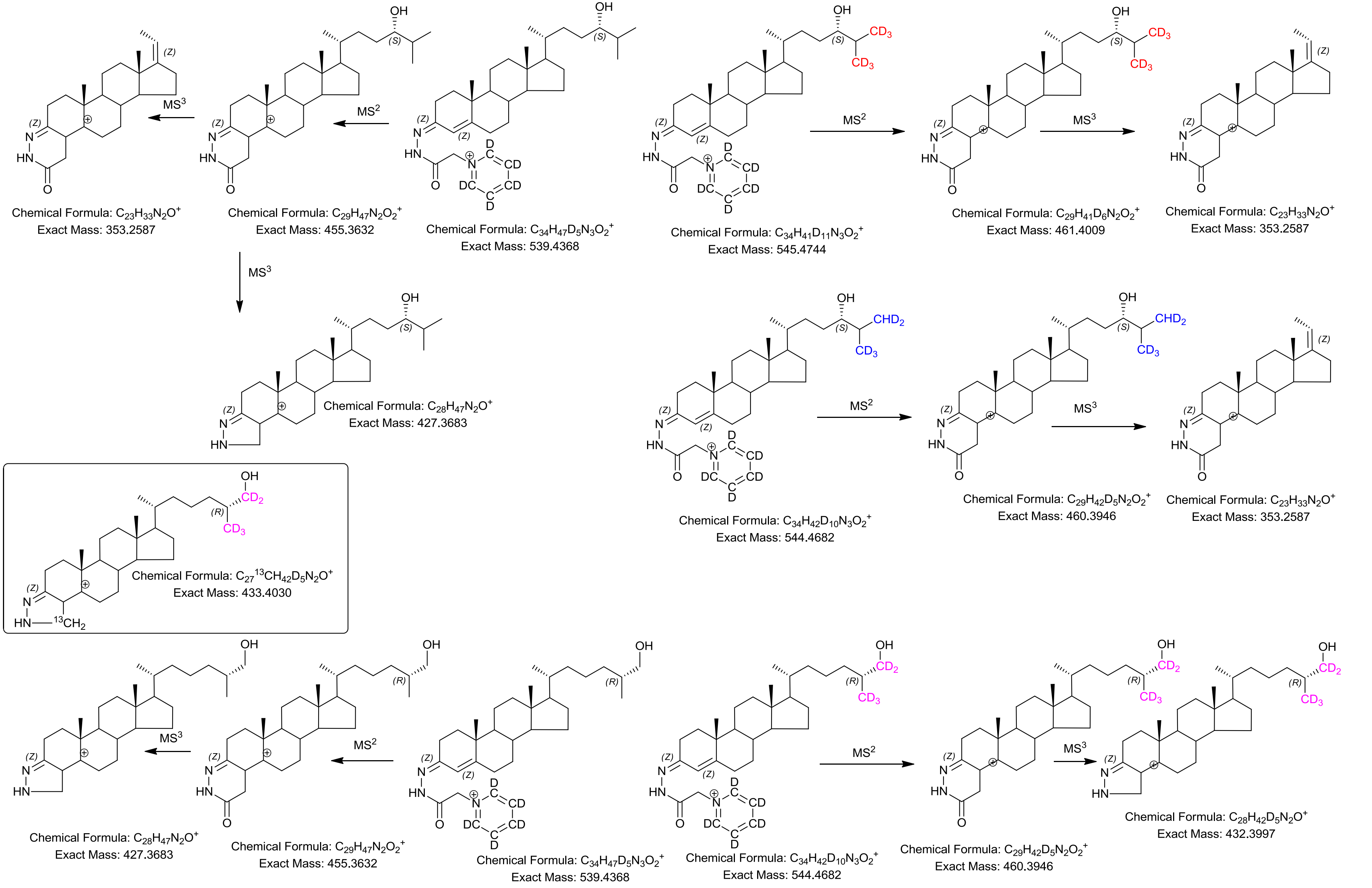

Figure S4A

PC_Plasma_Bjorkem_Mouse_Fed-Chol-D6_N... 12/02/13 12:18:30

PC_Plasma_Bjorkem_Mouse_Fed-Chol-D6_No-24OHC-D7_Fr1a-GPd5_ 45-1499 RT: 7.03-7.29 AV: 18 NL: 1.31E1
F: ITMS + c ESI Full ms3 553.42@cid30.00 469.34@cid35.00 [125.00-5!

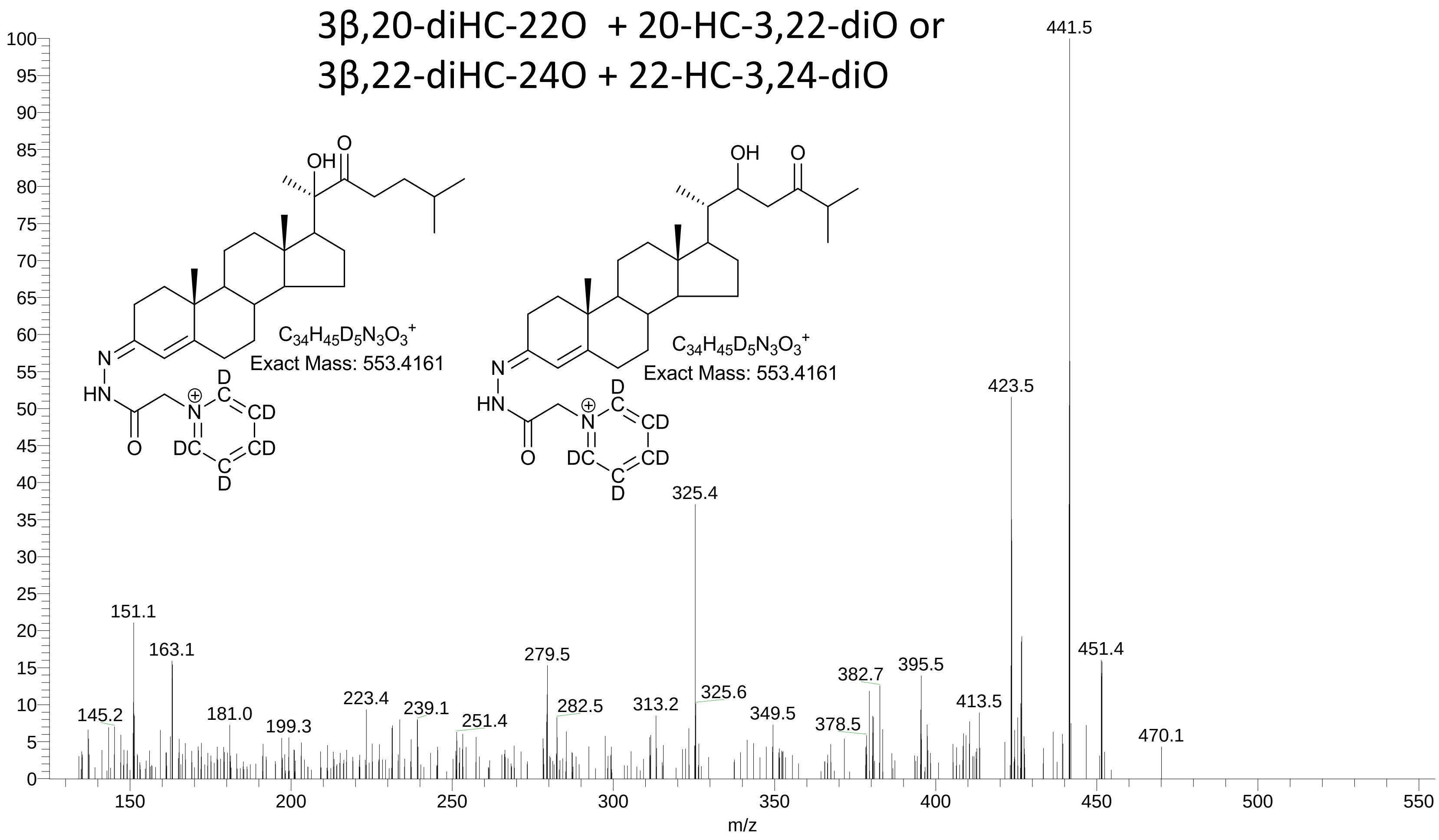

Figure S4B

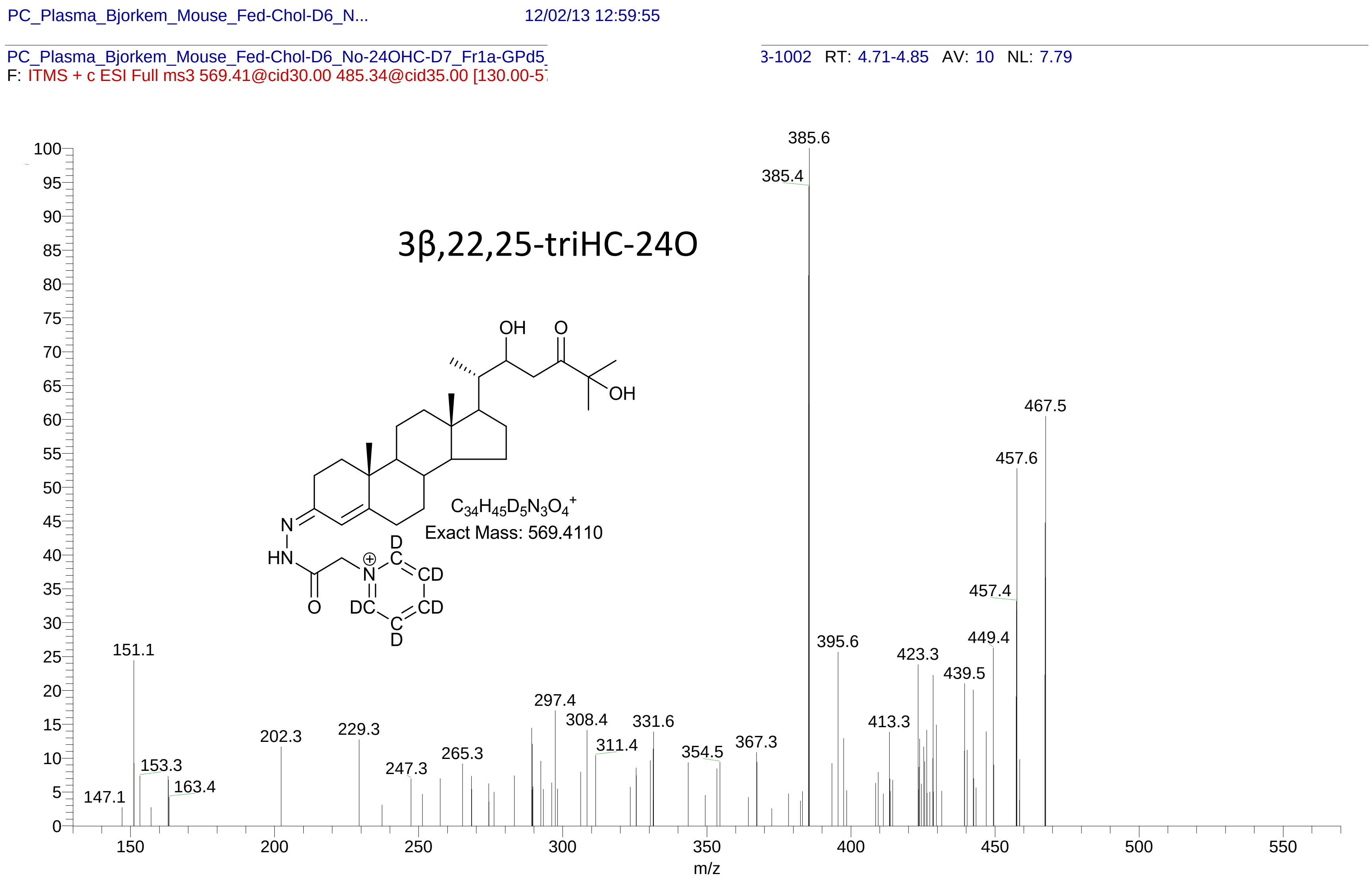
PC_Plasma_Bjorkem_Mouse_Fed-Chol-D6_N... 12/02/13 12:59:55
PC_Plasma_Bjorkem_Mouse_Fed-Chol-D6_No-24OHC-D7_Fr1a-GPd5_
3-1002 RT: 4.71-4.85 AV: 10 NL: 7.79
F: ITMS + c ESI Full ms3 569.41@cid30.00 485.34@cid35.00 [130.00-57
3β,22,25-triHC-24O
C34H45D5N3O4+
Exact Mass: 569.4110
385.6
385.4
467.5
457.6
457.4
449.4
439.5
423.3
413.3
395.6
367.3
354.5
331.6
311.4
308.4
297.4
265.3
247.3
229.3
202.3
163.4
153.3
151.1
147.1
m/z

Figure S5

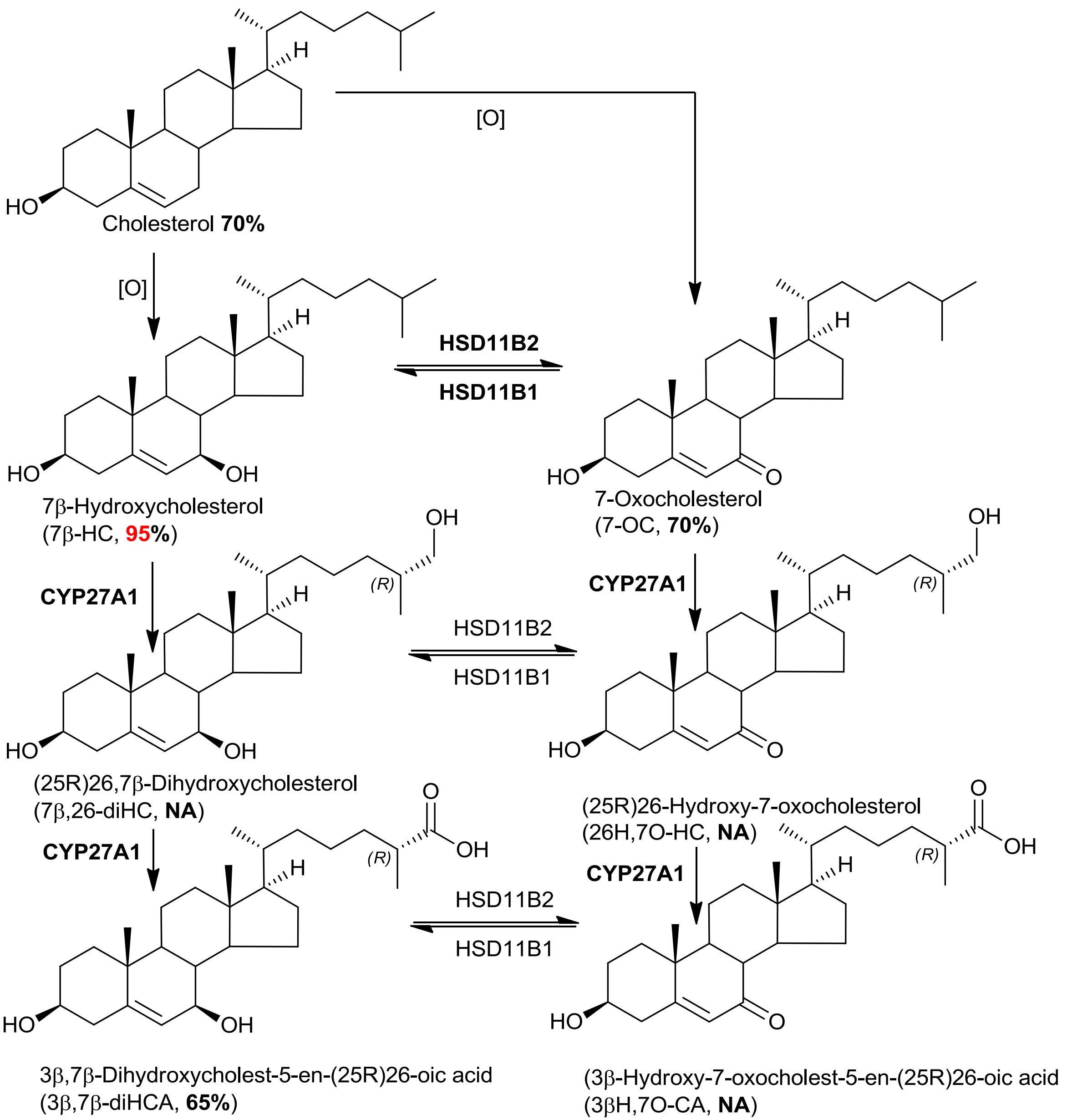

[O]
HO
Cholesterol 70%
[O]
HSD11B2
HSD11B1
HO
OH
7β-Hydroxycholesterol
(7β-HC, 95%)
HO
O
7-Oxocholesterol
(7-OC, 70%)
CYP27A1
OH
(R)
CYP27A1
OH
(R)
HSD11B2
HSD11B1
HO
OH
(25R)26,7β-Dihydroxycholesterol
(7β,26-diHC, NA)
HO
O
(25R)26-Hydroxy-7-oxocholesterol
(26H,7O-HC, NA)
CYP27A1
O
OH
(R)
CYP27A1
O
OH
(R)
HSD11B2
HSD11B1
HO
OH
HO
O
3β,7β-Dihydroxycholest-5-en-(25R)26-oic acid
(3β,7β-diHCA, 65%)
(3β-Hydroxy-7-oxocholest-5-en-(25R)26-oic acid
(3βH,7O-CA, NA)

Figure S6A

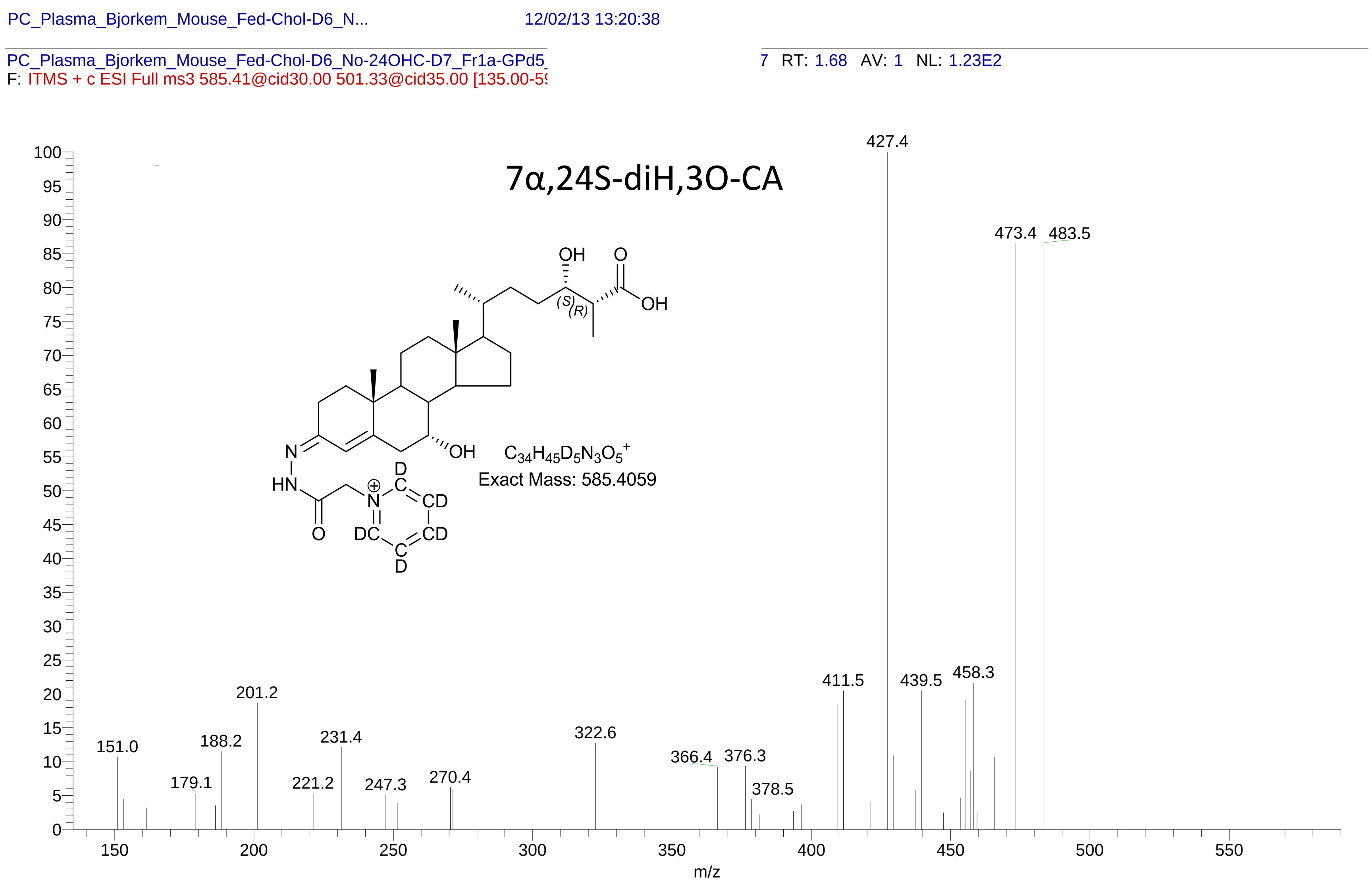
PC_Plasma_Bjorkem_Mouse_Fed-Chol-D6_N...
12/02/13 13:20:38
PC_Plasma_Bjorkem_Mouse_Fed-Chol-D6_No-24OHC-D7_Fr1a-GPd5_
7 RT: 1.68 AV: 1 NL: 1.23E2
F: ITMS + c ESI Full ms3 585.41@cid30.00 501.33@cid35.00 [135.00-5
7α,24S-diH,3O-CA
OH
O
OH
(S)
(R)
OH
N
HN
O
D
C
CD
CD
DC
C
D
N
C34H45D5N3O5+
Exact Mass: 585.4059
427.4
473.4
483.5
411.5
439.5
458.3
201.2
151.0
188.2
179.1
231.4
221.2
247.3
270.4
322.6
366.4
376.3
378.5
m/z

Figure S6B

PC_Plasma_Bjorkem_Mouse_Fed-Chol-D6_N... 12/02/13 13:20:38

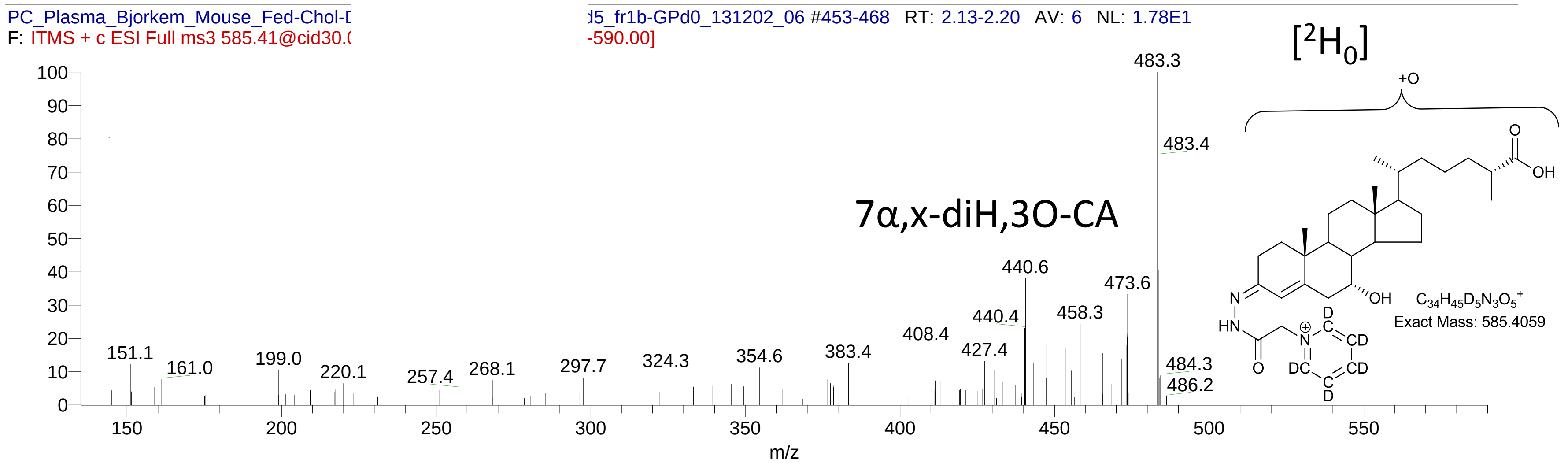

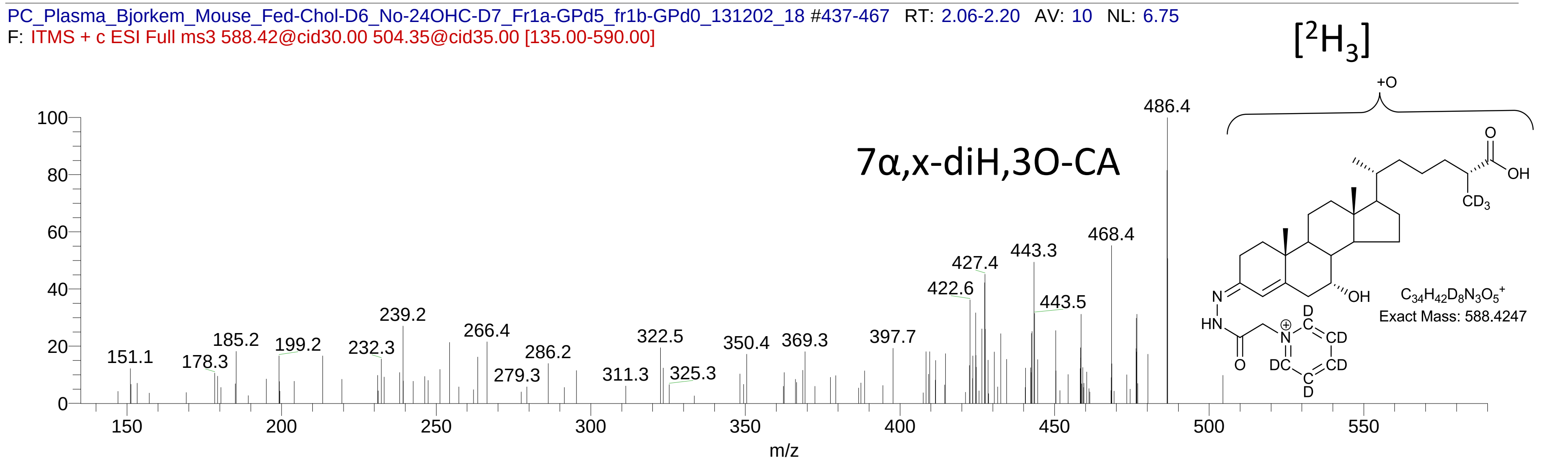

Figure S6C

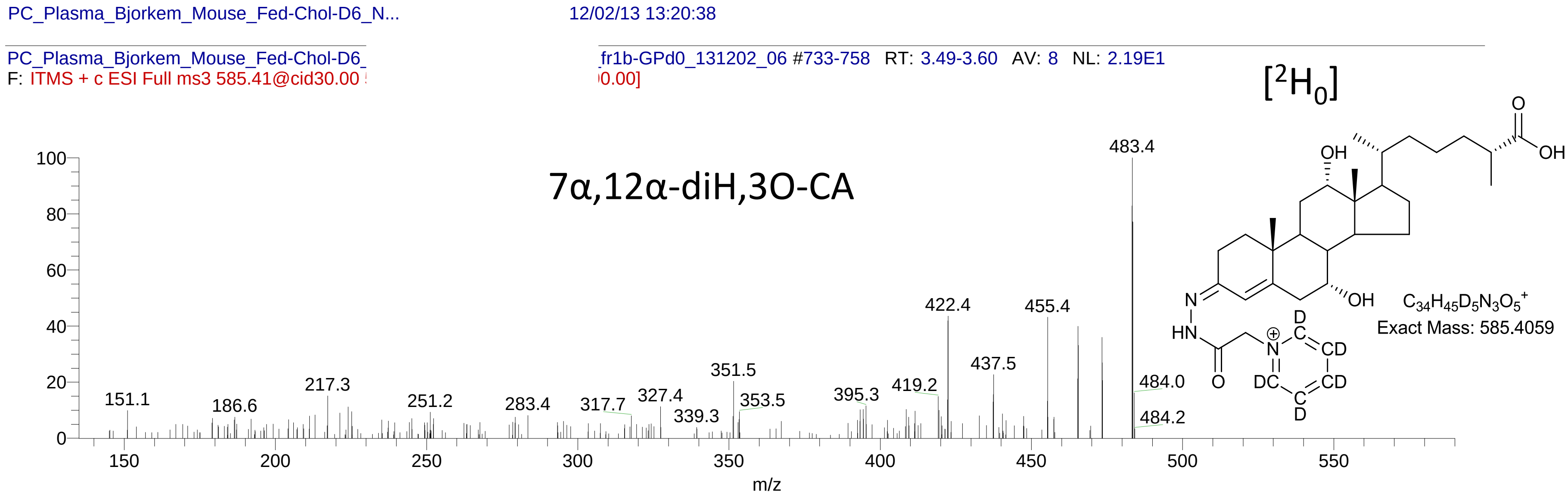

PC_Plasma_Bjorkem_Mouse_Fed-Chol-D6_N...
12/02/13 13:20:38
PC_Plasma_Bjorkem_Mouse_Fed-Chol-D6_
_fr1b-GPd0_131202_06 #733-758 RT: 3.49-3.60 AV: 8 NL: 2.19E1
F: ITMS + c ESI Full ms3 585.41@cid30.00
0.00]
[2H0]
7α,12α-diH,3O-CA
483.4
422.4
455.4
437.5
351.5
419.2
395.3
353.5
327.4
339.3
317.7
283.4
251.2
217.3
186.6
151.1
484.0
484.2
C34H45D5N3O5+
Exact Mass: 585.4059
m/z

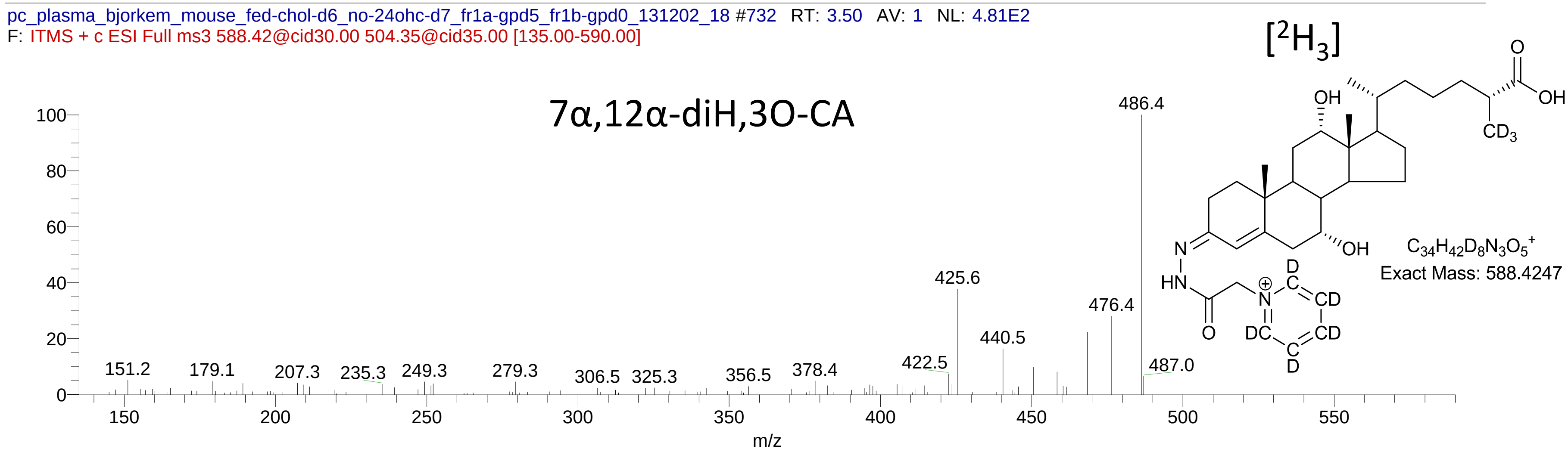

pc_plasma_bjorkem_mouse_fed-chol-d6_no-24ohc-d7_fr1a-gpd5_fr1b-gpd0_131202_18 #732 RT: 3.50 AV: 1 NL: 4.81E2
F: ITMS + c ESI Full ms3 588.42@cid30.00 504.35@cid35.00 [135.00-590.00]
[2H3]
7α,12α-diH,3O-CA
486.4
476.4
425.6
440.5
422.5
487.0
378.4
356.5
325.3
306.5
279.3
249.3
235.3
207.3
179.1
151.2
C34H42D8N3O5+
Exact Mass: 588.4247
m/z

Figure S6D

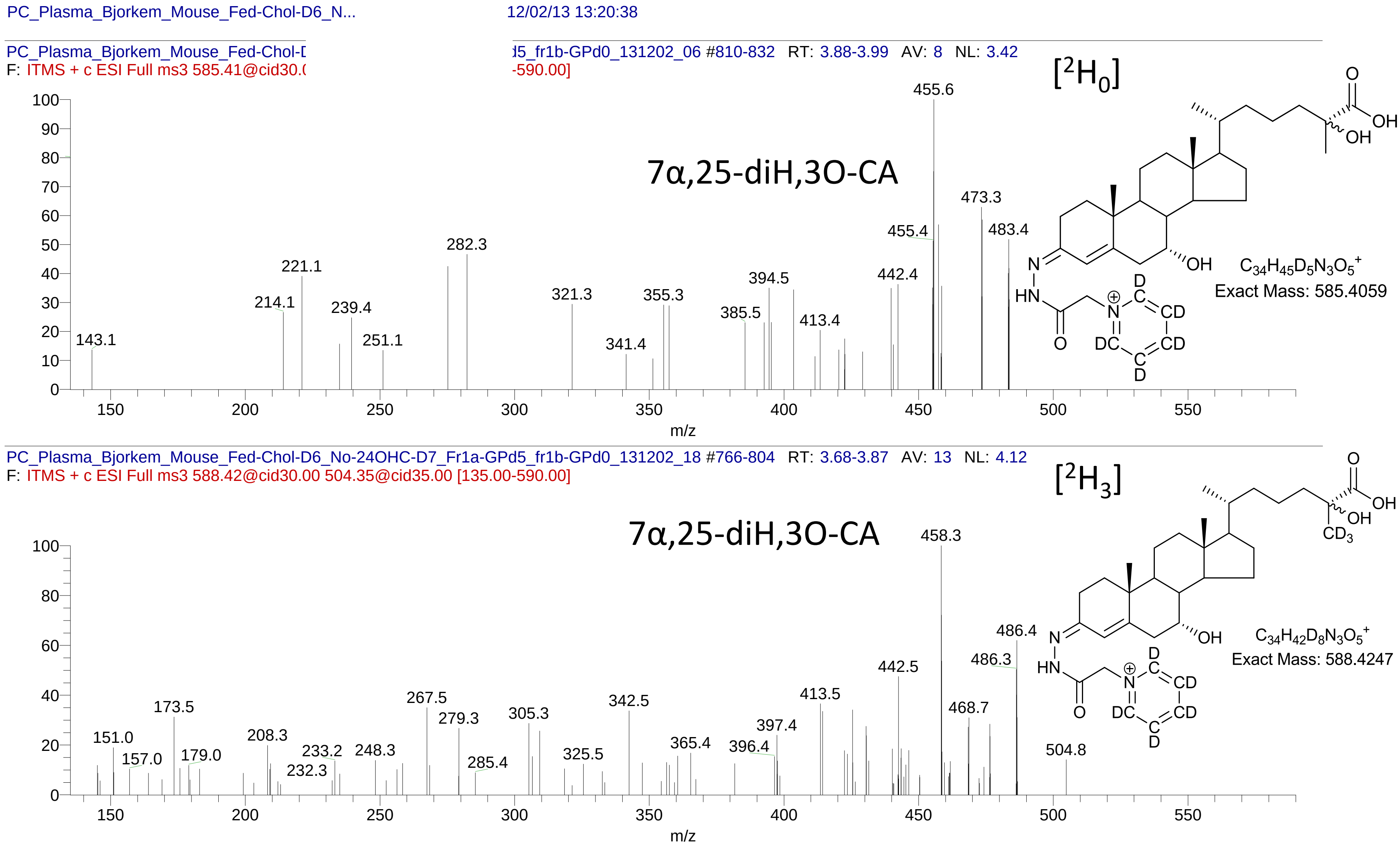
PC_Plasma_Bjorkem_Mouse_Fed-Chol-D6_N...
12/02/13 13:20:38
PC_Plasma_Bjorkem_Mouse_Fed-Chol-I
d5_fr1b-GPd0_131202_06 #810-832 RT: 3.88-3.99 AV: 8 NL: 3.42
F: ITMS + c ESI Full ms3 585.41@cid30.(
-590.00]
[2H0]
7α,25-diH,3O-CA
C34H45D5N3O5+
Exact Mass: 585.4059
m/z
PC_Plasma_Bjorkem_Mouse_Fed-Chol-D6_No-24OHC-D7_Fr1a-GPd5_fr1b-GPd0_131202_18 #766-804 RT: 3.68-3.87 AV: 13 NL: 4.12
F: ITMS + c ESI Full ms3 588.42@cid30.00 504.35@cid35.00 [135.00-590.00]
[2H3]
7α,25-diH,3O-CA
C34H42D8N3O5+
Exact Mass: 588.4247
m/z

Figure S7

Figure S7

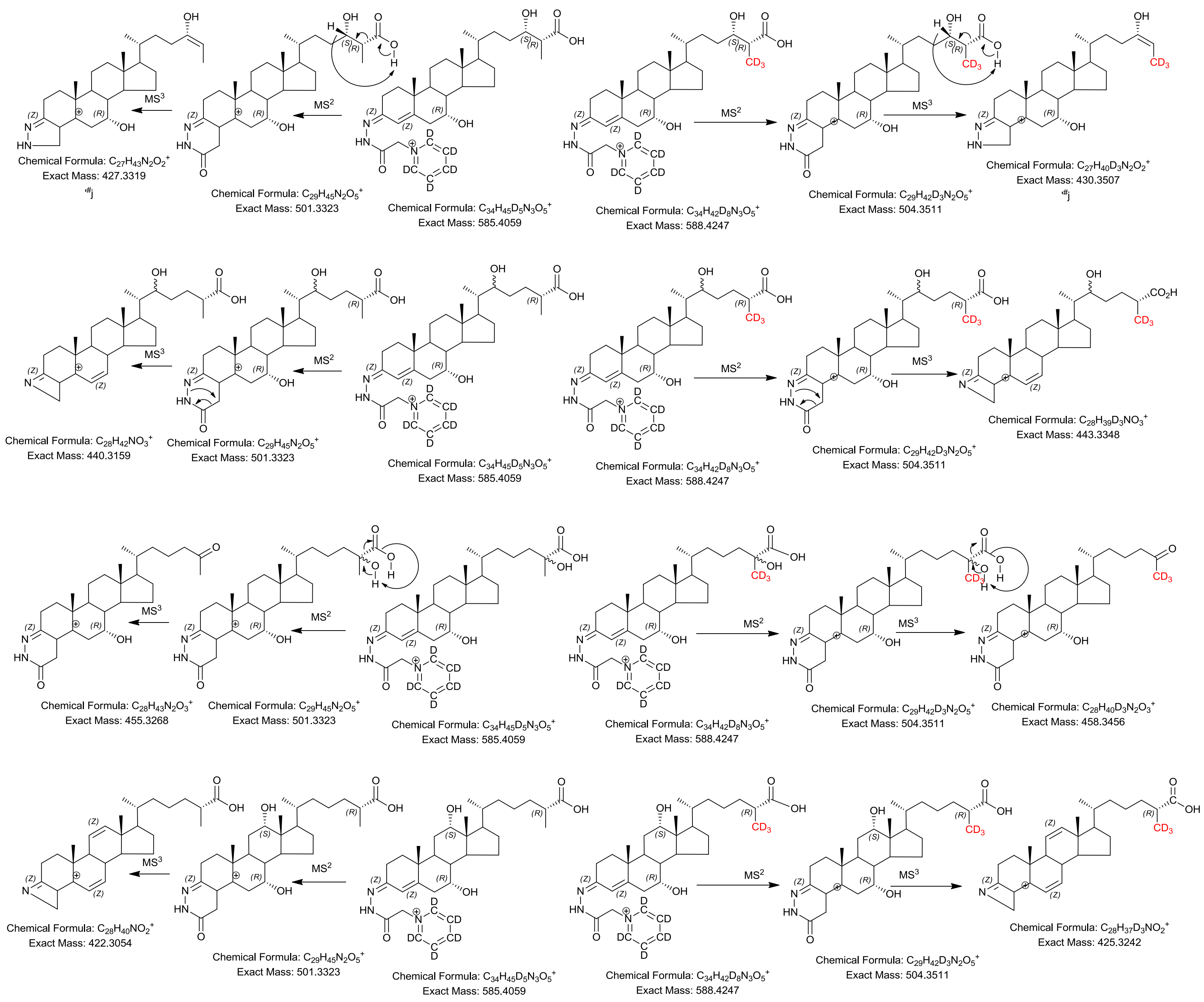

Figure S8A

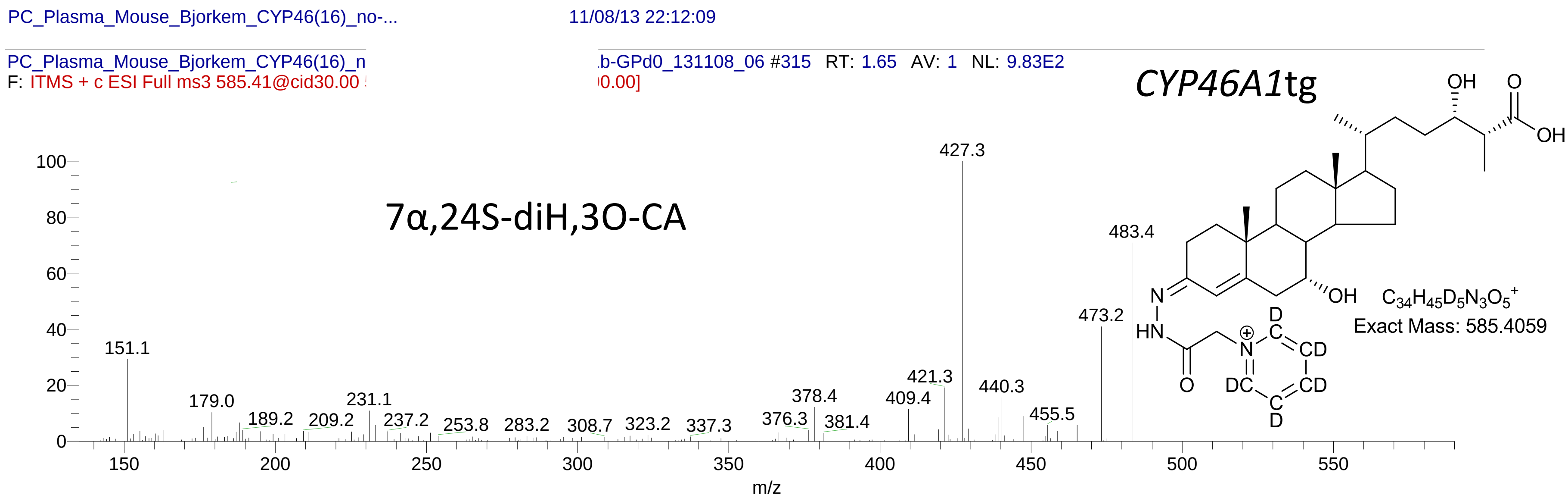

PC_Plasma_Mouse_Bjorkem_CYP46(16)_no-...
11/08/13 22:12:09
PC_Plasma_Mouse_Bjorkem_CYP46(16)_n
.b-GPd0_131108_06 #315 RT: 1.65 AV: 1 NL: 9.83E2
F: ITMS + c ESI Full ms3 585.41@cid30.00
)0.00]
CYP46A1tg
7α,24S-diH,3O-CA
C34H45D5N3O5+
Exact Mass: 585.4059
427.3
483.4
473.2
151.1
421.3
409.4
440.3
378.4
455.5
179.0
231.1
189.2
209.2
237.2
253.8
283.2
308.7
323.2
337.3
376.3
381.4
m/z

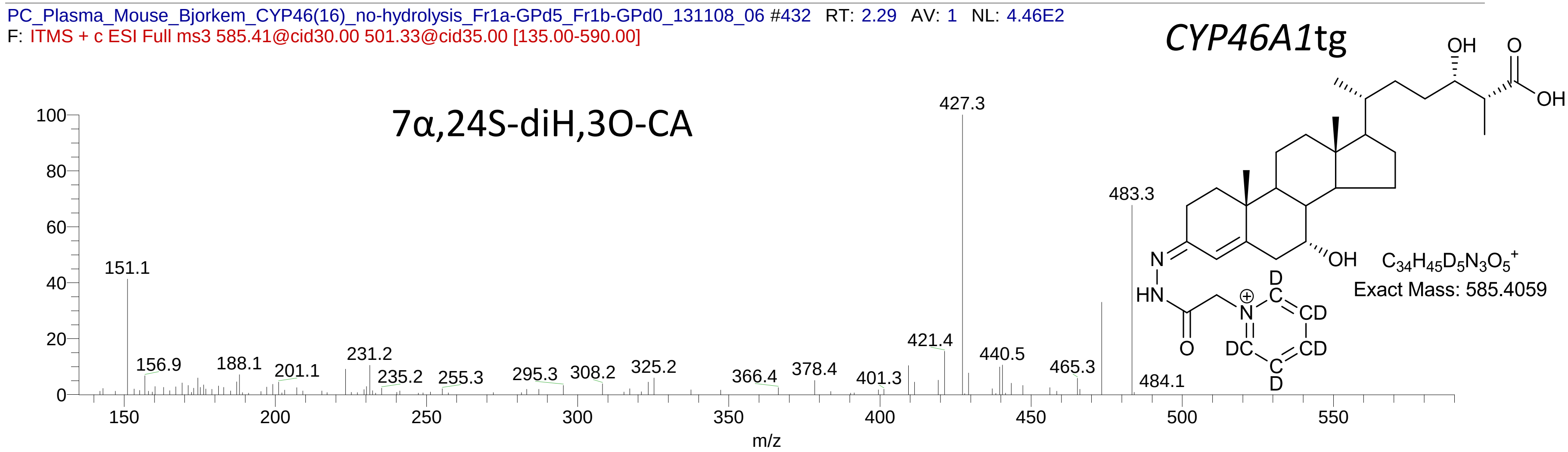

PC_Plasma_Mouse_Bjorkem_CYP46(16)_no-hydrolysis_Fr1a-GPd5_Fr1b-GPd0_131108_06 #432 RT: 2.29 AV: 1 NL: 4.46E2
F: ITMS + c ESI Full ms3 585.41@cid30.00 501.33@cid35.00 [135.00-590.00]
CYP46A1tg
7α,24S-diH,3O-CA
C34H45D5N3O5+
Exact Mass: 585.4059
427.3
483.3
151.1
421.4
440.5
156.9
188.1
201.1
231.2
235.2
255.3
295.3
308.2
325.2
366.4
378.4
401.3
465.3
484.1
m/z

Figure S8B

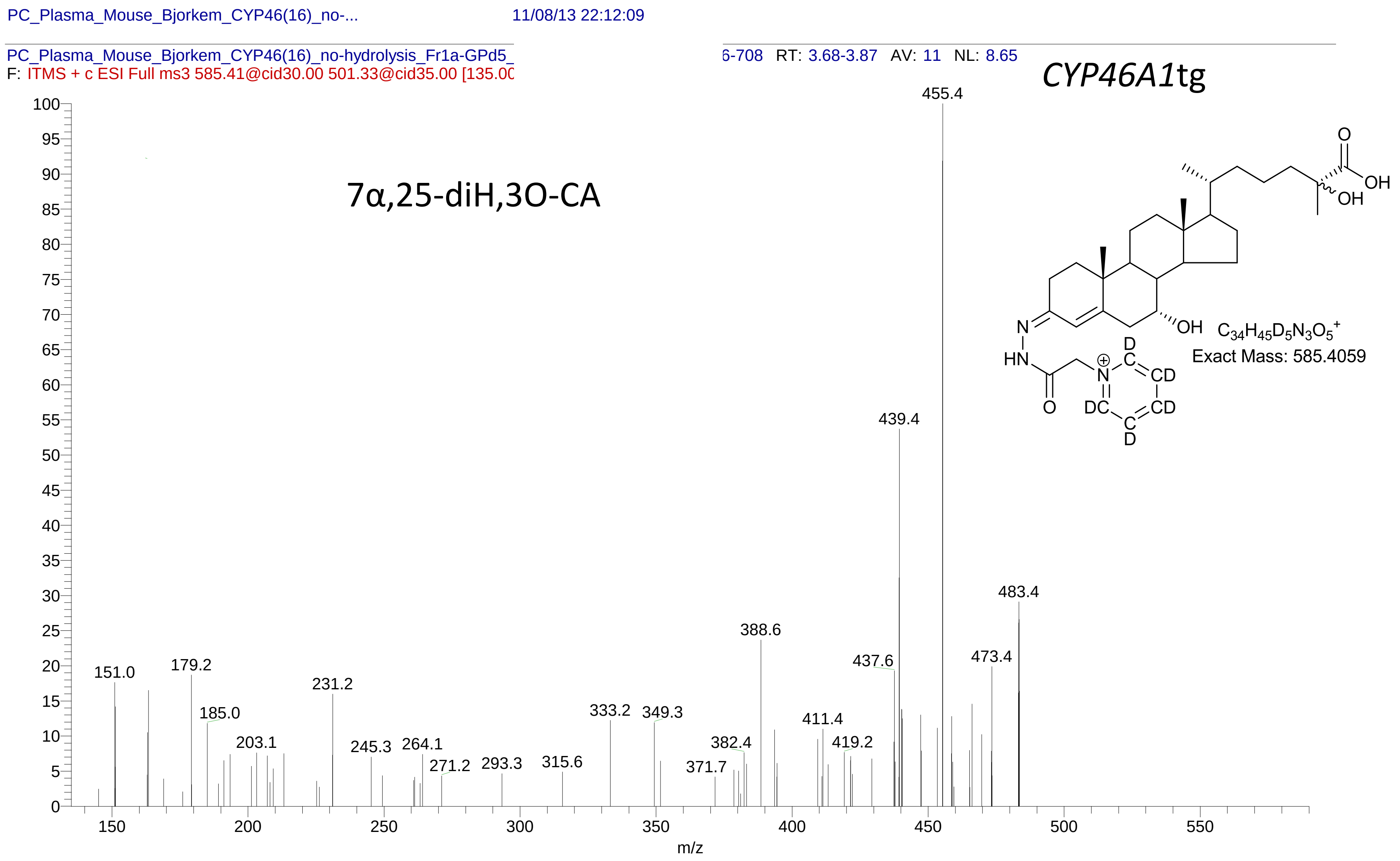

PC_Plasma_Mouse_Bjorkem_CYP46(16)_no-...
11/08/13 22:12:09
PC_Plasma_Mouse_Bjorkem_CYP46(16)_no-hydrolysis_Fr1a-GPd5_
6-708 RT: 3.68-3.87 AV: 11 NL: 8.65
F: ITMS + c ESI Full ms3 585.41@cid30.00 501.33@cid35.00 [135.00
CYP46A1tg
7α,25-diH,3O-CA
455.4
439.4
483.4
388.6
437.6
473.4
151.0
179.2
231.2
185.0
333.2
349.3
411.4
203.1
245.3
264.1
382.4
419.2
271.2
293.3
315.6
371.7
$C_{34}H_{45}D_5N_3O_5^+$
Exact Mass: 585.4059
m/z

Figure S8C

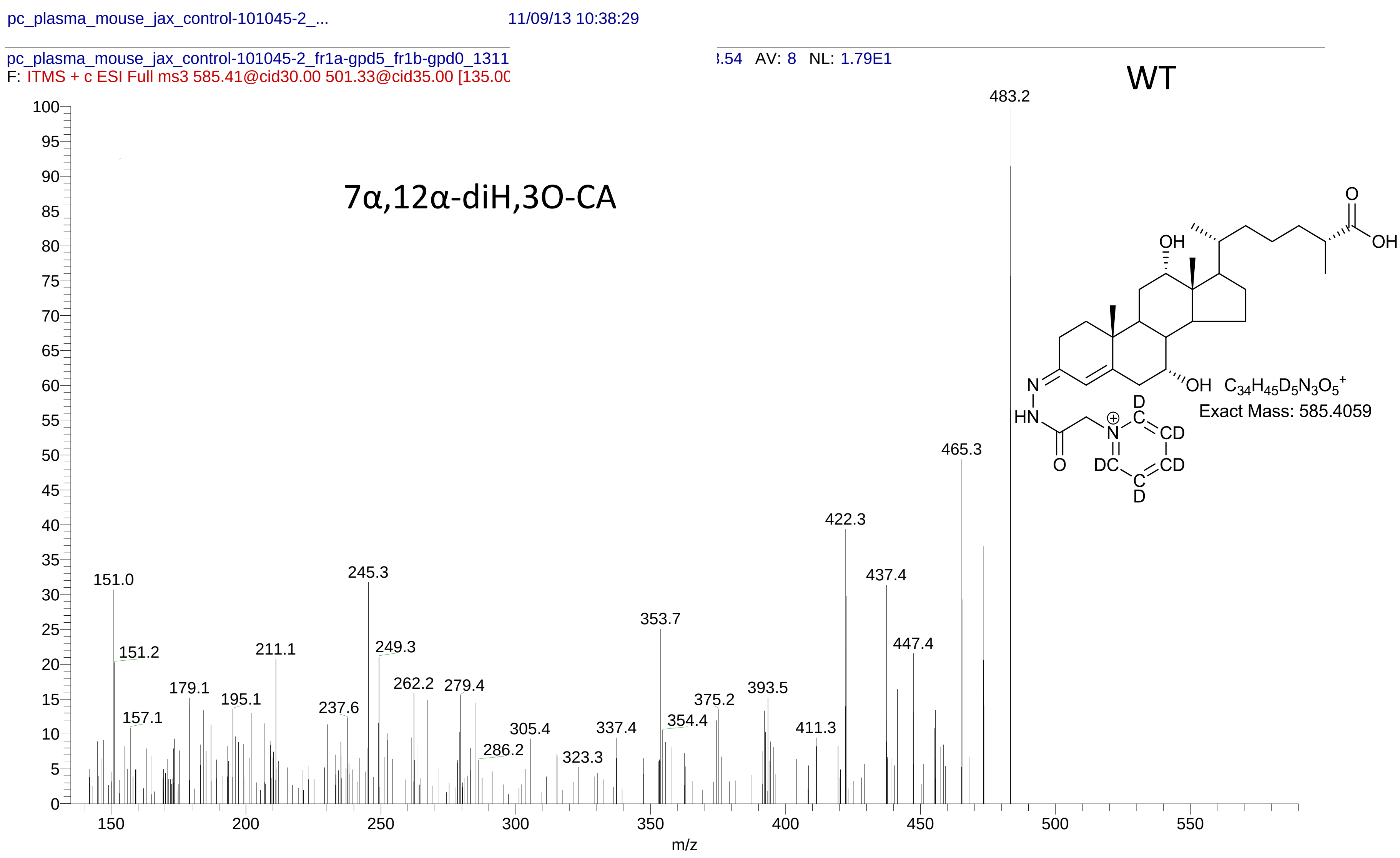
pc_plasma_mouse_jax_control-101045-2_...
11/09/13 10:38:29
pc_plasma_mouse_jax_control-101045-2_fr1a-gpd5_fr1b-gpd0_1311
.54 AV: 8 NL: 1.79E1
F: ITMS + c ESI Full ms3 585.41@cid30.00 501.33@cid35.00 [135.00
WT
7α,12α-diH,3O-CA
483.2
465.3
422.3
437.4
447.4
353.7
245.3
151.0
151.2
211.1
249.3
157.1
179.1
195.1
237.6
262.2
279.4
286.2
305.4
323.3
337.4
354.4
375.2
393.5
411.3
m/z
OH
O
OH
N
HN
O
D
C
CD
CD
DC
C
D
N
OH
C34H45D5N3O5+
Exact Mass: 585.4059

Figure S9A

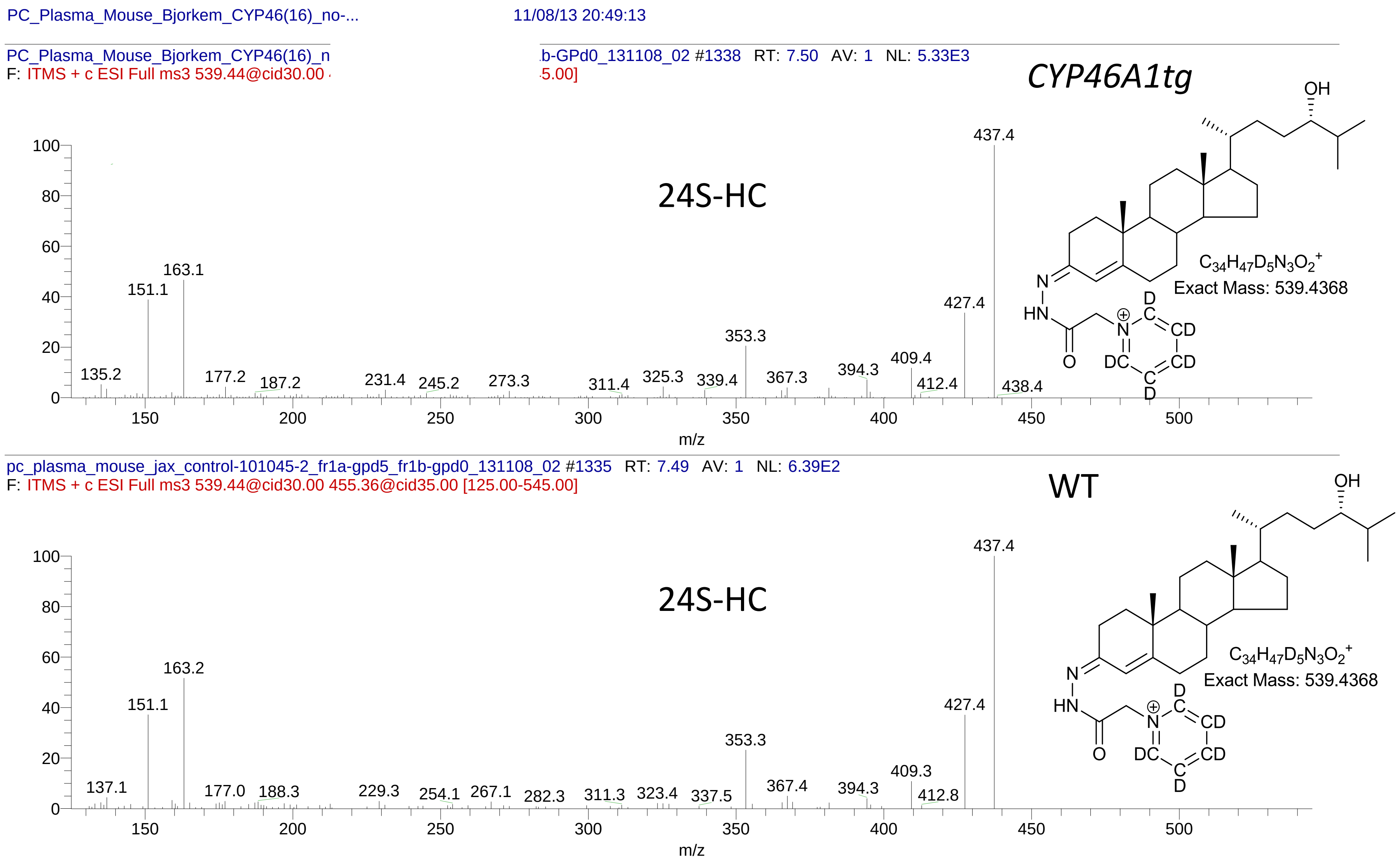

PC_Plasma_Mouse_Bjorkem_CYP46(16)_no-...
11/08/13 20:49:13
PC_Plasma_Mouse_Bjorkem_CYP46(16)_n
.b-GPd0_131108_02 #1338 RT: 7.50 AV: 1 NL: 5.33E3
F: ITMS + c ESI Full ms3 539.44@cid30.00
.5.00]
CYP46A1tg
24S-HC
437.4
163.1
151.1
427.4
353.3
409.4
394.3
135.2
177.2
187.2
231.4
245.2
273.3
311.4
325.3
339.4
367.3
412.4
438.4
OH
N
HN
O
DC
CD
D
C
$C_{34}H_{47}D_5N_3O_2^+$
Exact Mass: 539.4368
m/z
pc_plasma_mouse_jax_control-101045-2_fr1a-gpd5_fr1b-gpd0_131108_02 #1335 RT: 7.49 AV: 1 NL: 6.39E2
F: ITMS + c ESI Full ms3 539.44@cid30.00 455.36@cid35.00 [125.00-545.00]
WT
24S-HC
437.4
163.2
151.1
427.4
353.3
409.3
137.1
177.0
188.3
229.3
254.1
267.1
282.3
311.3
323.4
337.5
367.4
394.3
412.8
$C_{34}H_{47}D_5N_3O_2^+$
Exact Mass: 539.4368
m/z

Figure S9B

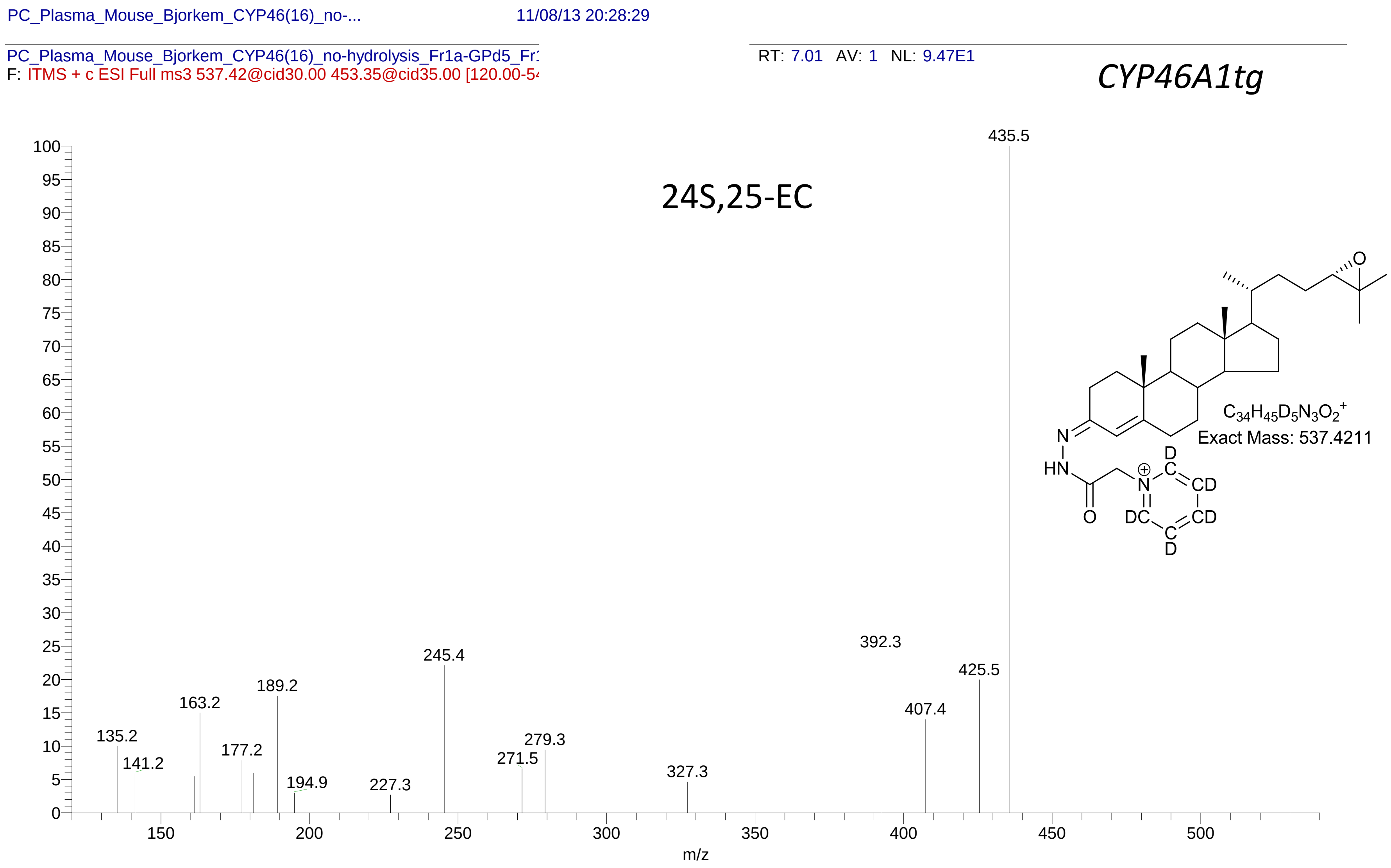
PC_Plasma_Mouse_Bjorkem_CYP46(16)_no-...
11/08/13 20:28:29
PC_Plasma_Mouse_Bjorkem_CYP46(16)_no-hydrolysis_Fr1a-GPd5_Fr
F: ITMS + c ESI Full ms3 537.42@cid30.00 453.35@cid35.00 [120.00-5
RT: 7.01 AV: 1 NL: 9.47E1
CYP46A1tg
24S,25-EC
435.5
392.3
425.5
407.4
245.4
189.2
163.2
135.2
141.2
177.2
194.9
227.3
279.3
271.5
327.3
m/z
C34H45D5N3O2+
Exact Mass: 537.4211

Figure 9C

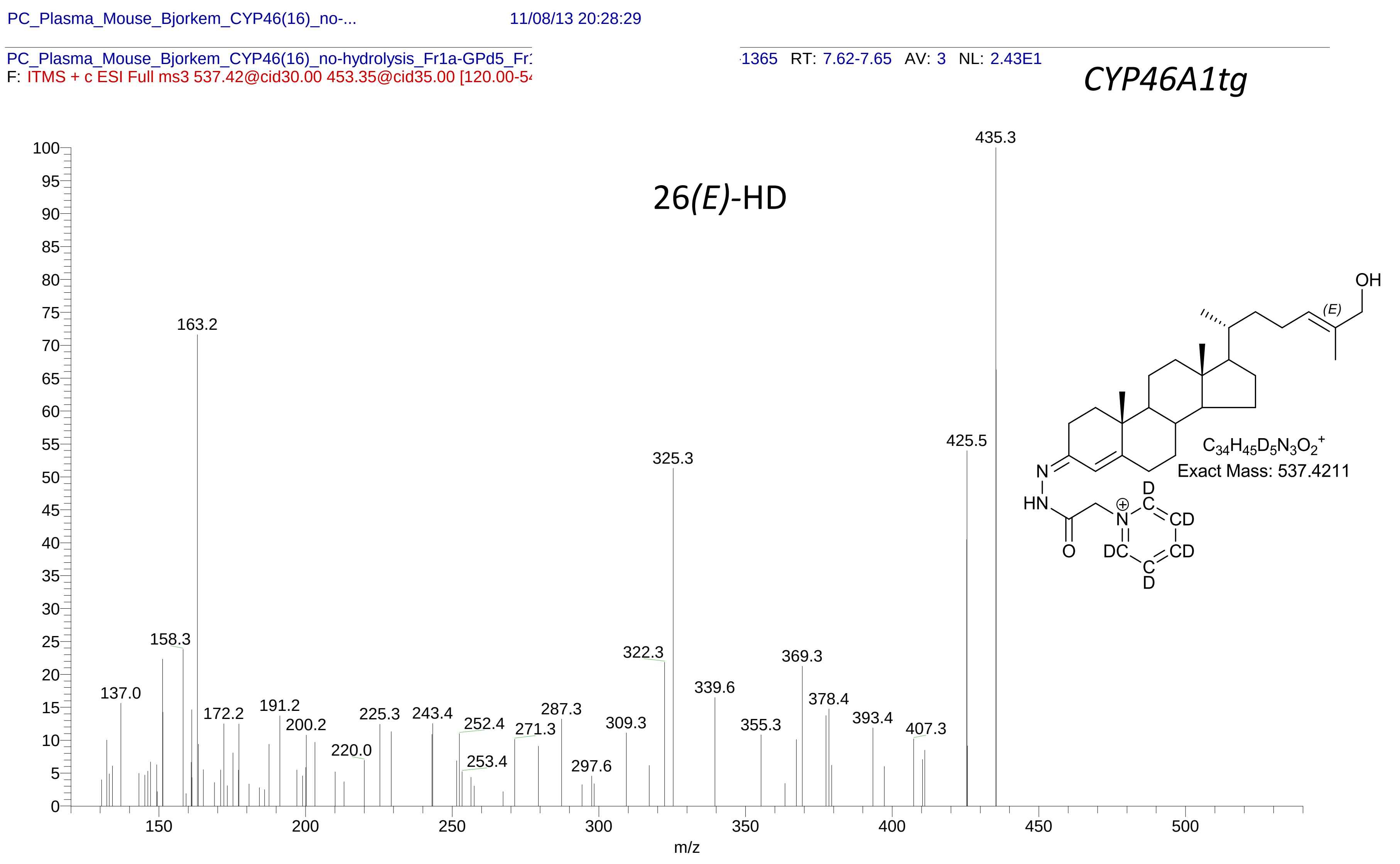
PC_Plasma_Mouse_Bjorkem_CYP46(16)_no-...
11/08/13 20:28:29
PC_Plasma_Mouse_Bjorkem_CYP46(16)_no-hydrolysis_Fr1a-GPd5_Fr
1365 RT: 7.62-7.65 AV: 3 NL: 2.43E1
F: ITMS + c ESI Full ms3 537.42@cid30.00 453.35@cid35.00 [120.00-5
CYP46A1tg
26(E)-HD
435.3
163.2
425.5
325.3
158.3
322.3
369.3
137.0
339.6
378.4
172.2
191.2
200.2
225.3
243.4
252.4
271.3
287.3
309.3
355.3
393.4
407.3
220.0
253.4
297.6
m/z
C34H45D5N3O2+
Exact Mass: 537.4211

Figure S9D

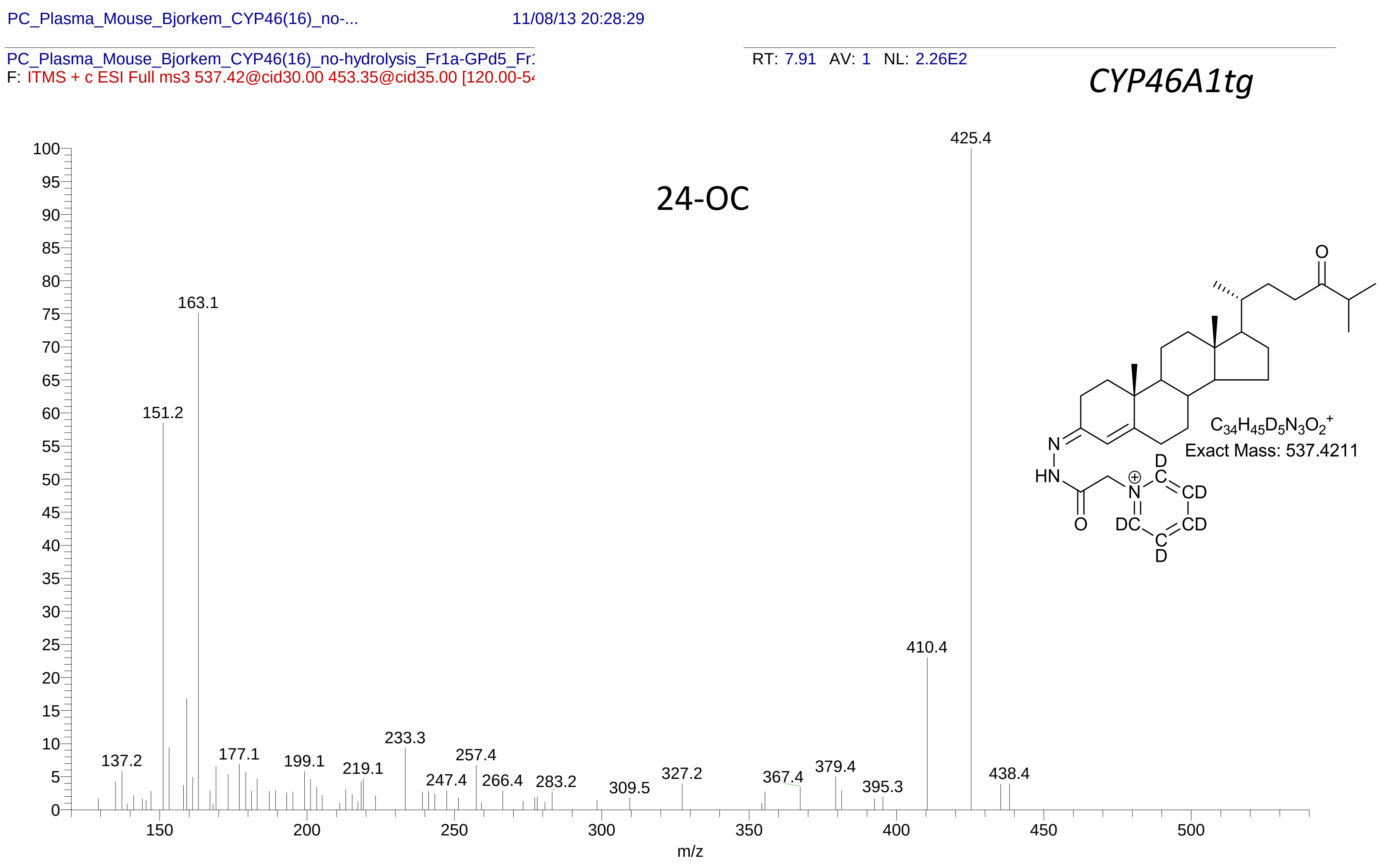
PC_Plasma_Mouse_Bjorkem_CYP46(16)_no-...
11/08/13 20:28:29
PC_Plasma_Mouse_Bjorkem_CYP46(16)_no-hydrolysis_Fr1a-GPd5_Fr1
F: ITMS + c ESI Full ms3 537.42@cid30.00 453.35@cid35.00 [120.00-5
RT: 7.91 AV: 1 NL: 2.26E2
CYP46A1tg
24-OC
425.4
163.1
151.2
410.4
137.2
177.1
199.1
219.1
233.3
247.4
257.4
266.4
283.2
309.5
327.2
367.4
379.4
395.3
438.4
m/z
C34H45D5N3O2+
Exact Mass: 537.4211

Figure S10

Figure S10

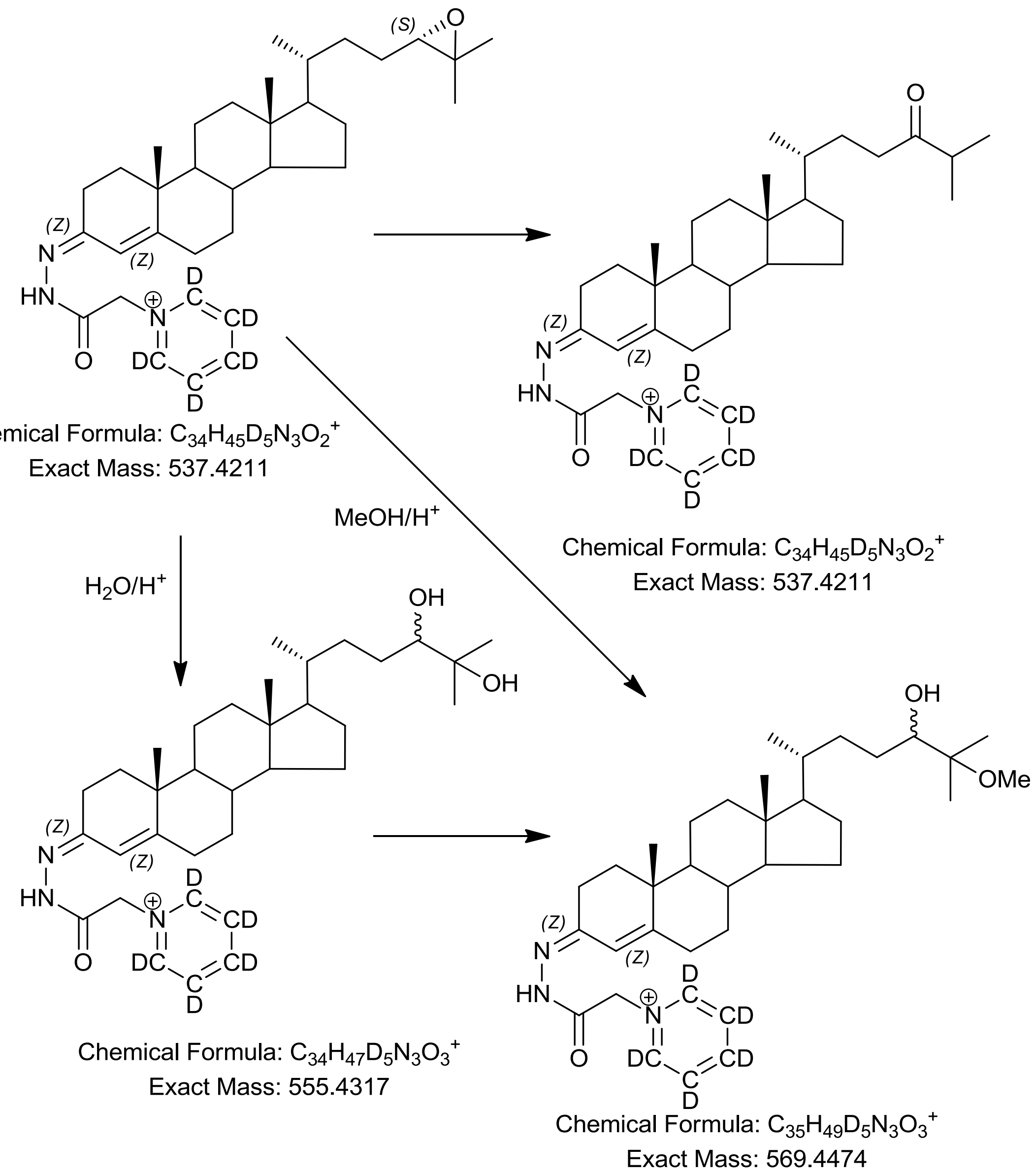

Figure S11A

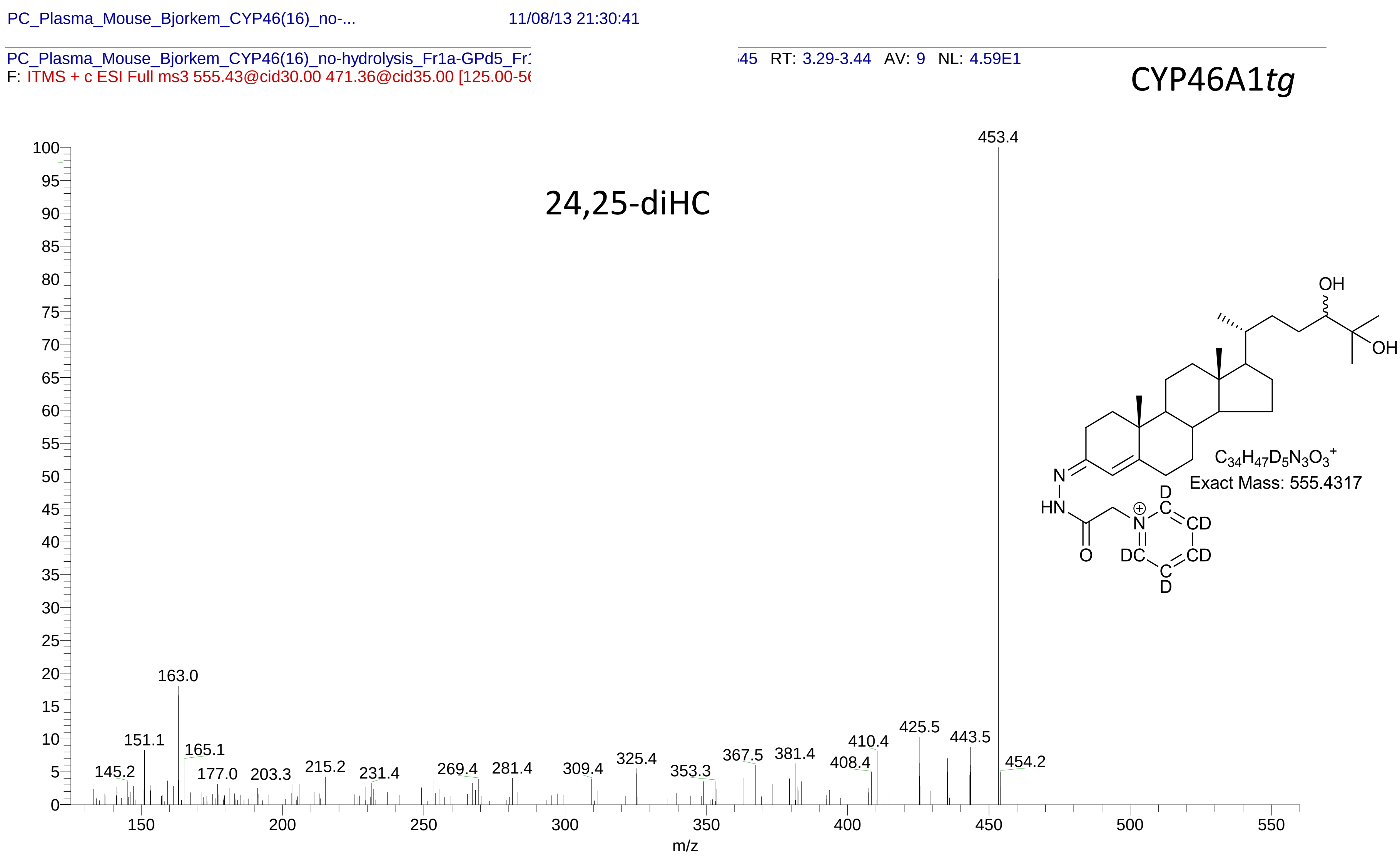
PC_Plasma_Mouse_Bjorkem_CYP46(16)_no-...
11/08/13 21:30:41
PC_Plasma_Mouse_Bjorkem_CYP46(16)_no-hydrolysis_Fr1a-GPd5_Fr
45 RT: 3.29-3.44 AV: 9 NL: 4.59E1
F: ITMS + c ESI Full ms3 555.43@cid30.00 471.36@cid35.00 [125.00-5
CYP46A1*tg*
24,25-diHC
453.4
163.0
151.1
145.2
165.1
177.0
203.3
215.2
231.4
269.4
281.4
309.4
325.4
353.3
367.5
381.4
408.4
410.4
425.5
443.5
454.2
m/z
OH
OH
N
HN
O
D
C
CD
CD
DC
C
D
N
$C_{34}H_{47}D_5N_3O_3^+$
Exact Mass: 555.4317

Figure S11B

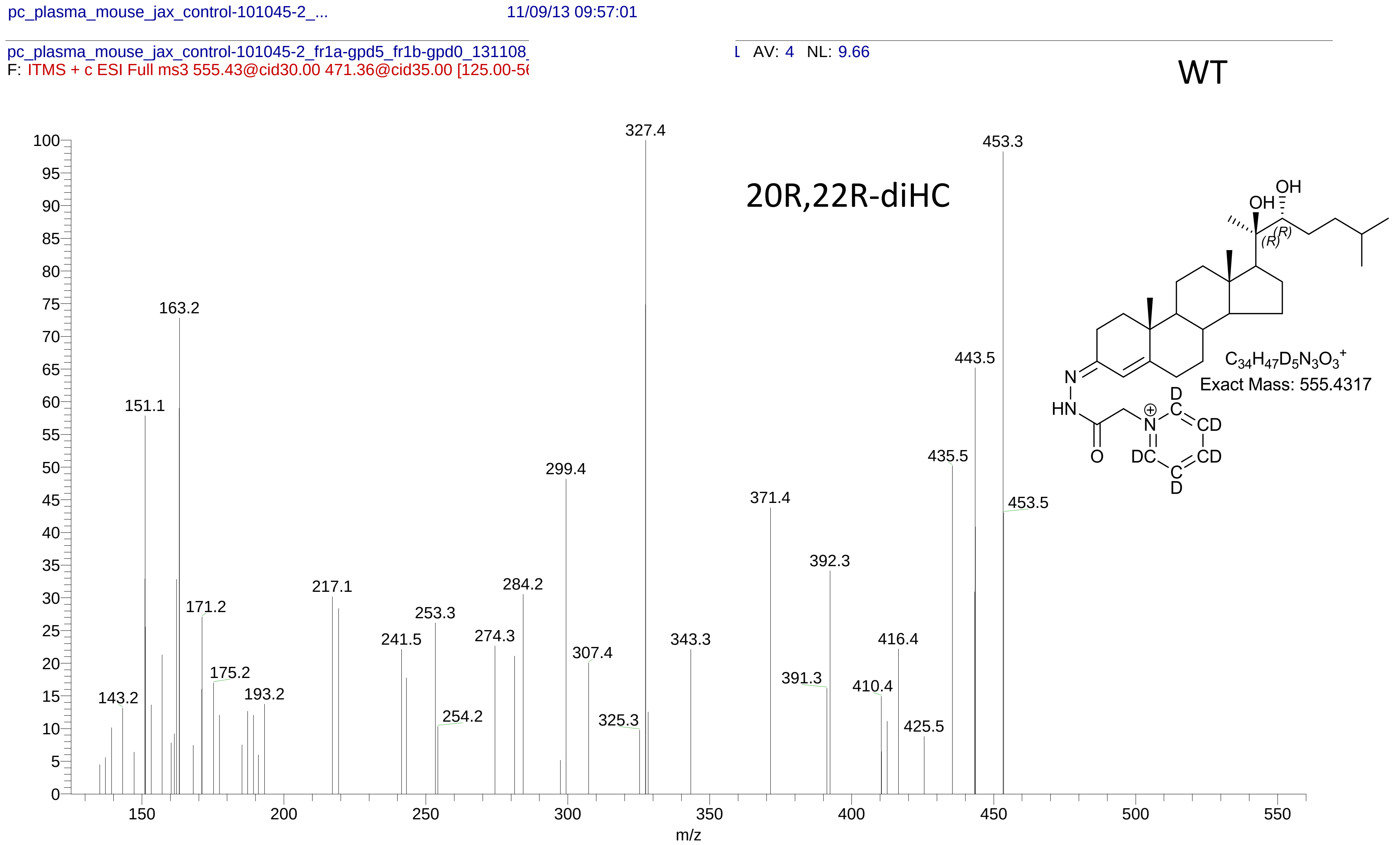

pc_plasma_mouse_jax_control-101045-2_...
11/09/13 09:57:01
pc_plasma_mouse_jax_control-101045-2_fr1a-gpd5_fr1b-gpd0_131108_
AV: 4 NL: 9.66
F: ITMS + c ESI Full ms3 555.43@cid30.00 471.36@cid35.00 [125.00-56
WT
20R,22R-diHC
C34H47D5N3O3+
Exact Mass: 555.4317
327.4
453.3
163.2
151.1
443.5
435.5
299.4
453.5
371.4
392.3
217.1
284.2
171.2
253.3
274.3
241.5
343.3
416.4
307.4
175.2
391.3
410.4
143.2
193.2
254.2
325.3
425.5
m/z

Figure S11C

PC_Plasma_Mouse_Bjorkem_CYP46(16)_no-... 11/08/13 21:30:41

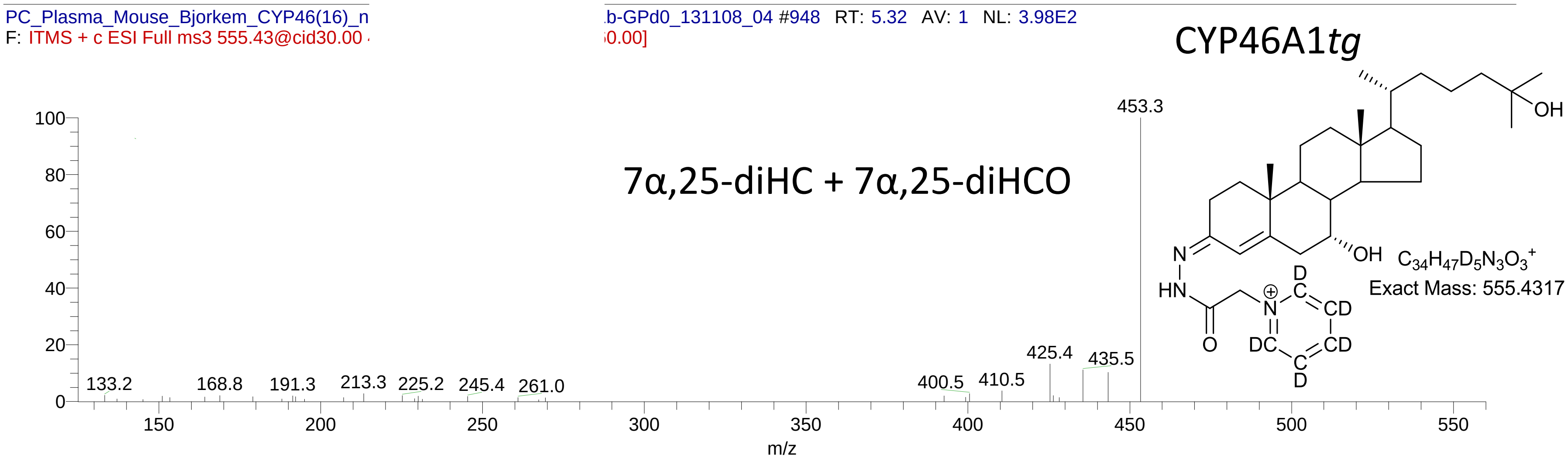

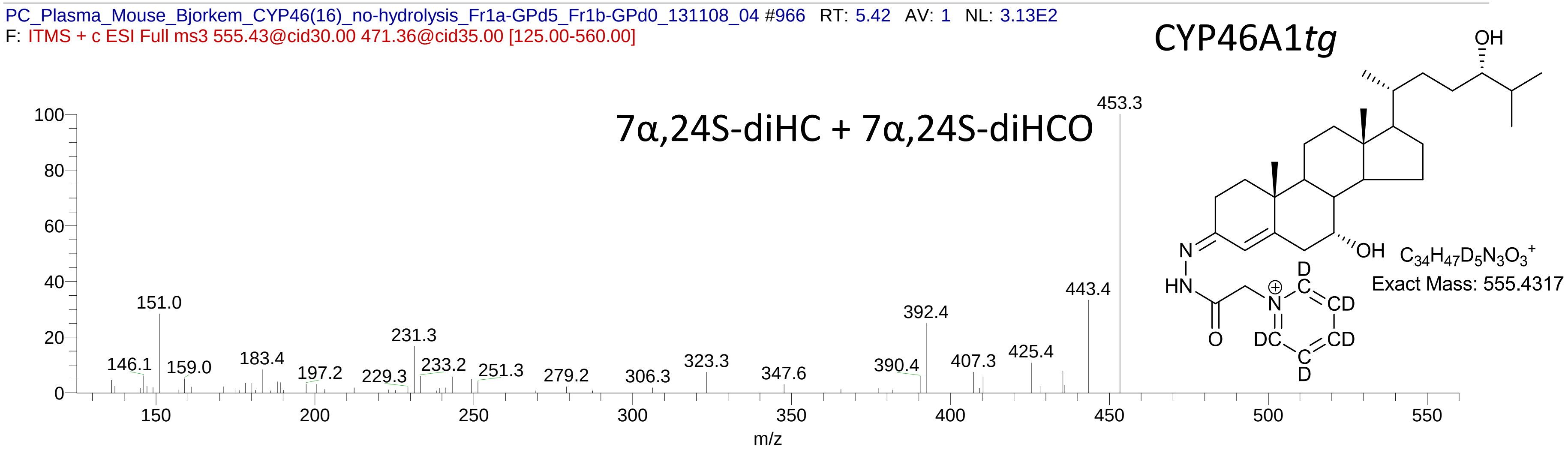

Figure S11D

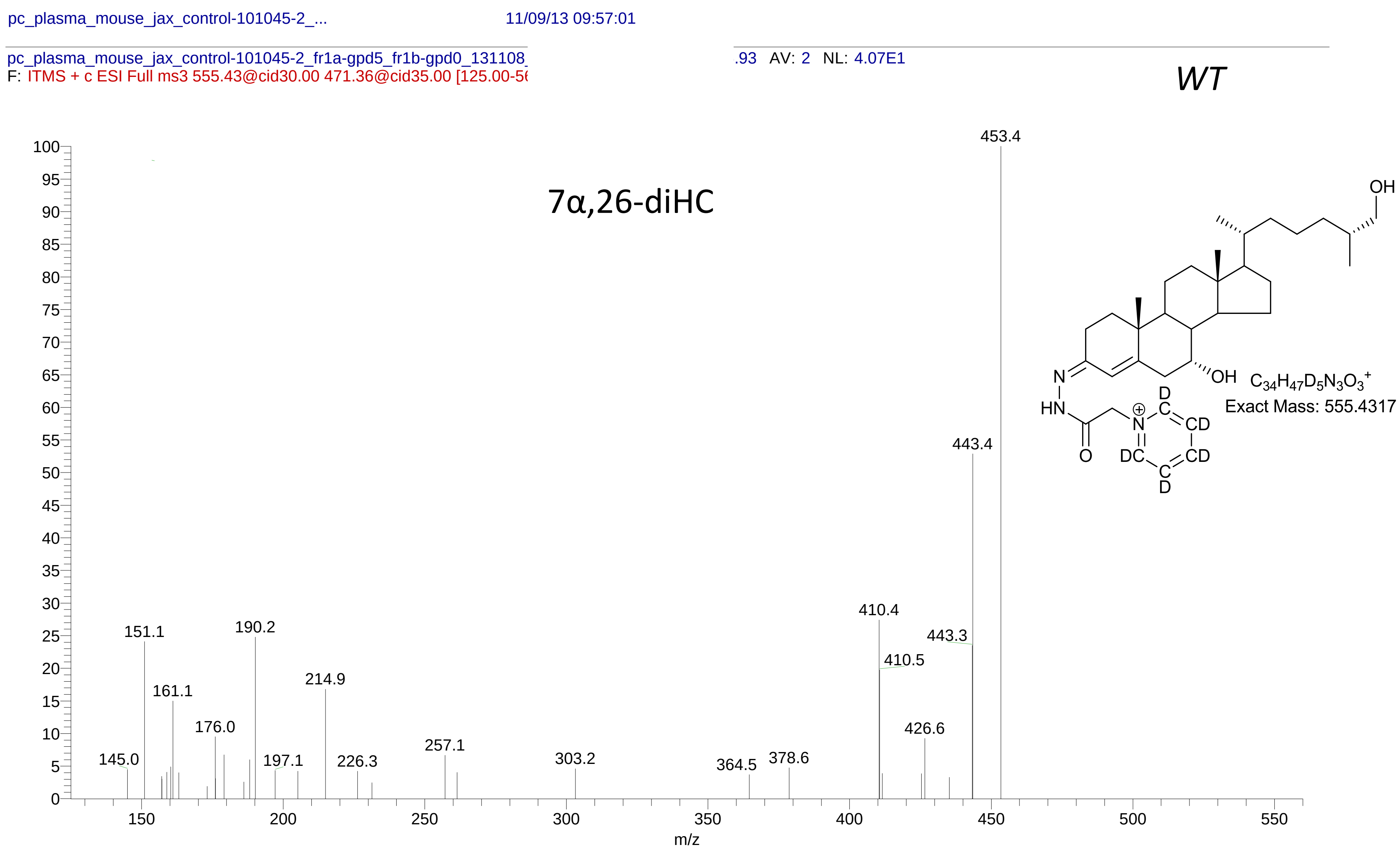

pc_plasma_mouse_jax_control-101045-2_...
11/09/13 09:57:01
pc_plasma_mouse_jax_control-101045-2_fr1a-gpd5_fr1b-gpd0_131108_
.93 AV: 2 NL: 4.07E1
F: ITMS + c ESI Full ms3 555.43@cid30.00 471.36@cid35.00 [125.00-5(
WT
7α,26-diHC
453.4
443.4
410.4
443.3
410.5
426.6
151.1
190.2
161.1
214.9
176.0
257.1
145.0
197.1
226.3
303.2
364.5
378.6
m/z
OH
N
HN
O
D
C
CD
CD
DC
C
D
OH
C34H47D5N3O3+
Exact Mass: 555.4317

Figure S11E

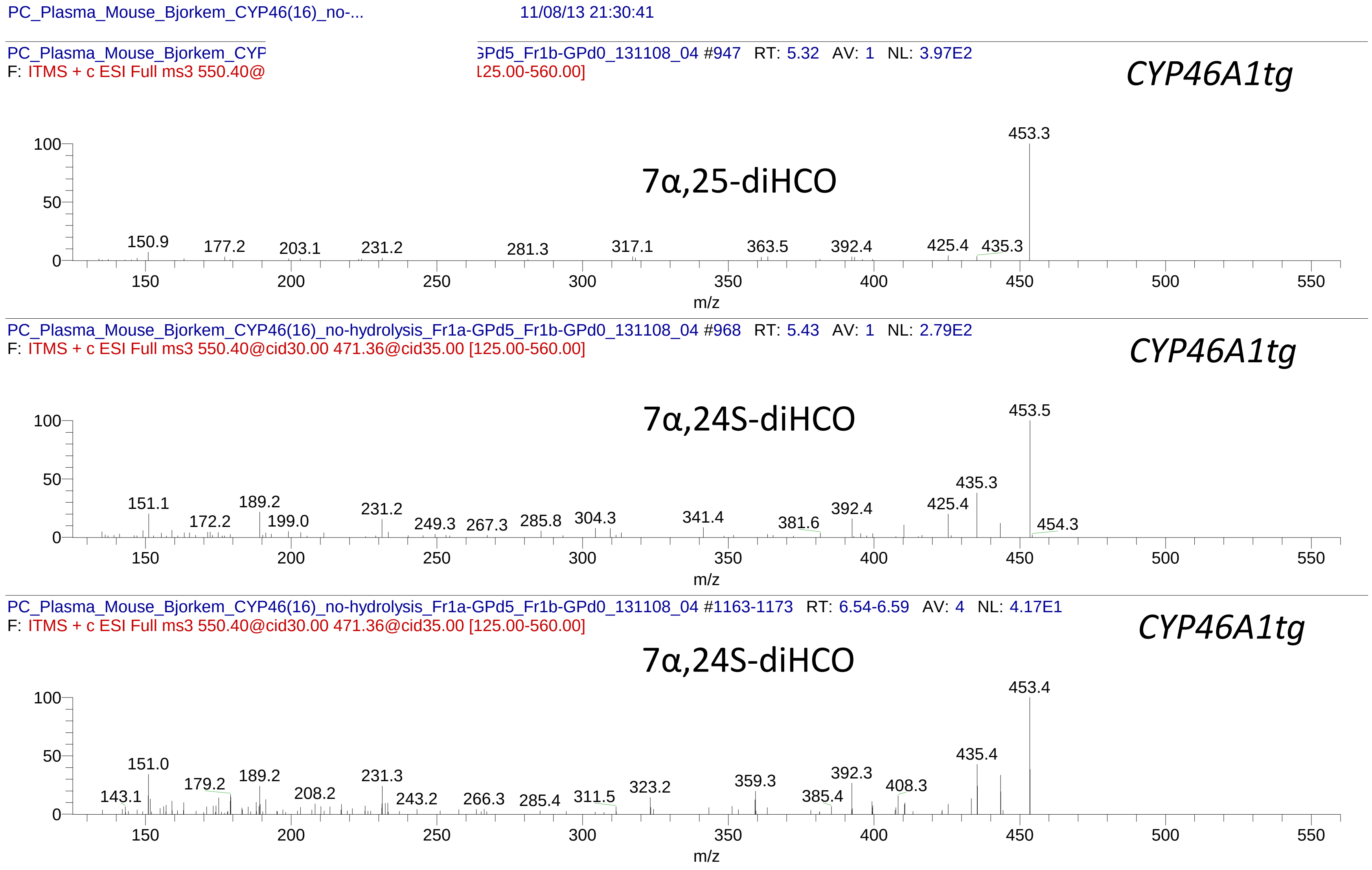
PC_Plasma_Mouse_Bjorkem_CYP46(16)_no-...
11/08/13 21:30:41
PC_Plasma_Mouse_Bjorkem_CYP … GPd5_Fr1b-GPd0_131108_04 #947 RT: 5.32 AV: 1 NL: 3.97E2
F: ITMS + c ESI Full ms3 550.40@ … 125.00-560.00]
CYP46A1tg
7α,25-diHCO
453.3
150.9
177.2
203.1
231.2
281.3
317.1
363.5
392.4
425.4
435.3
100
50
0
150
200
250
300
350
400
450
500
550
m/z
PC_Plasma_Mouse_Bjorkem_CYP46(16)_no-hydrolysis_Fr1a-GPd5_Fr1b-GPd0_131108_04 #968 RT: 5.43 AV: 1 NL: 2.79E2
F: ITMS + c ESI Full ms3 550.40@cid30.00 471.36@cid35.00 [125.00-560.00]
CYP46A1tg
7α,24S-diHCO
453.5
435.3
425.4
392.4
381.6
341.4
304.3
285.8
267.3
249.3
231.2
199.0
189.2
172.2
151.1
454.3
100
50
0
150
200
250
300
350
400
450
500
550
m/z
PC_Plasma_Mouse_Bjorkem_CYP46(16)_no-hydrolysis_Fr1a-GPd5_Fr1b-GPd0_131108_04 #1163-1173 RT: 6.54-6.59 AV: 4 NL: 4.17E1
F: ITMS + c ESI Full ms3 550.40@cid30.00 471.36@cid35.00 [125.00-560.00]
CYP46A1tg
7α,24S-diHCO
453.4
435.4
408.3
392.3
385.4
359.3
323.2
311.5
285.4
266.3
243.2
231.3
208.2
189.2
179.2
151.0
143.1
100
50
0
150
200
250
300
350
400
450
500
550
m/z

Figure S11F

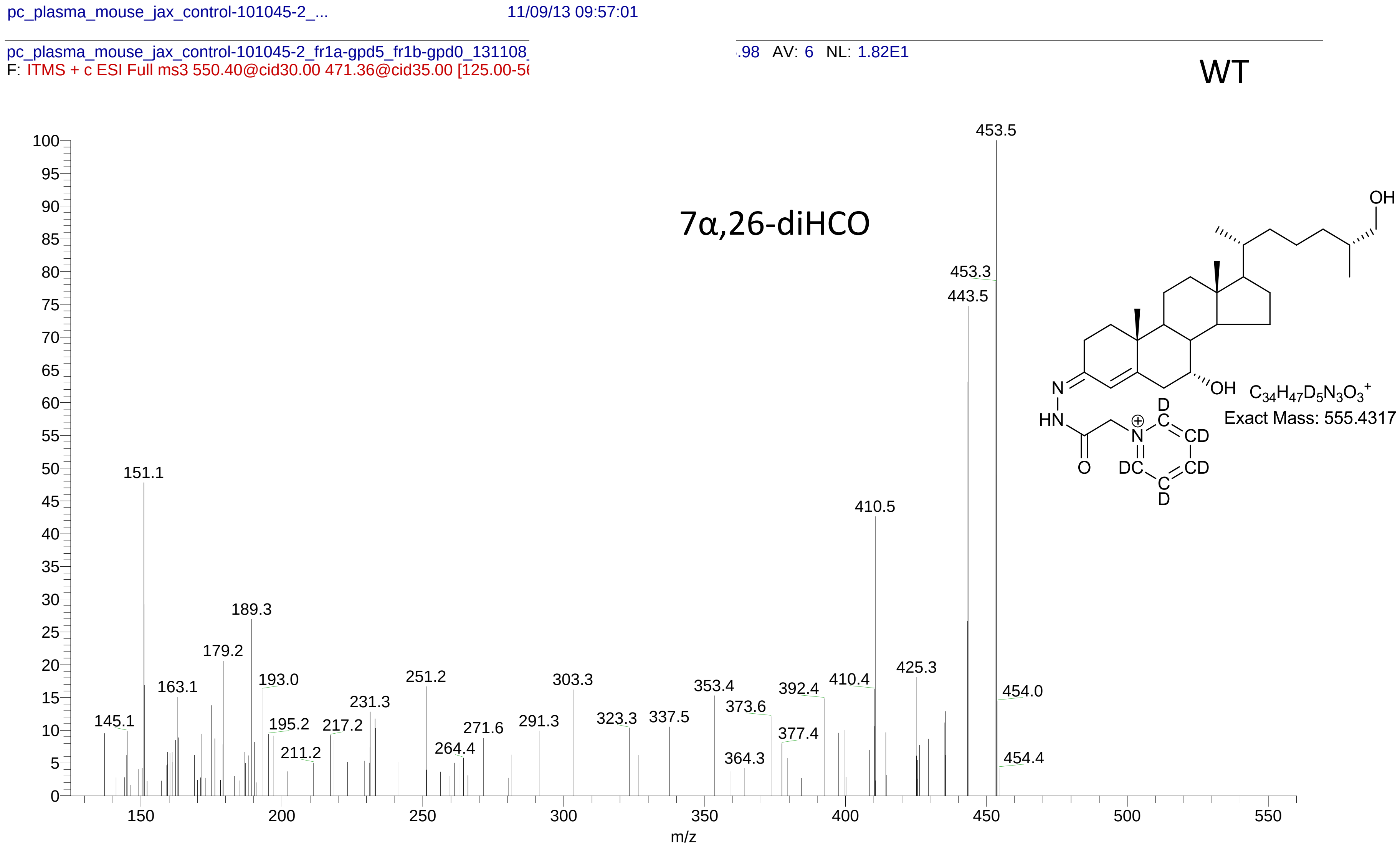

pc_plasma_mouse_jax_control-101045-2_...
11/09/13 09:57:01
pc_plasma_mouse_jax_control-101045-2_fr1a-gpd5_fr1b-gpd0_131108_
.98 AV: 6 NL: 1.82E1
F: ITMS + c ESI Full ms3 550.40@cid30.00 471.36@cid35.00 [125.00-5
WT
7α,26-diHCO
C34H47D5N3O3+
Exact Mass: 555.4317
453.5
453.3
443.5
410.5
151.1
189.3
179.2
163.1
193.0
145.1
195.2
217.2
211.2
231.3
251.2
264.4
271.6
291.3
303.3
323.3
337.5
353.4
373.6
364.3
392.4
377.4
410.4
425.3
454.0
454.4
m/z

Figure S12

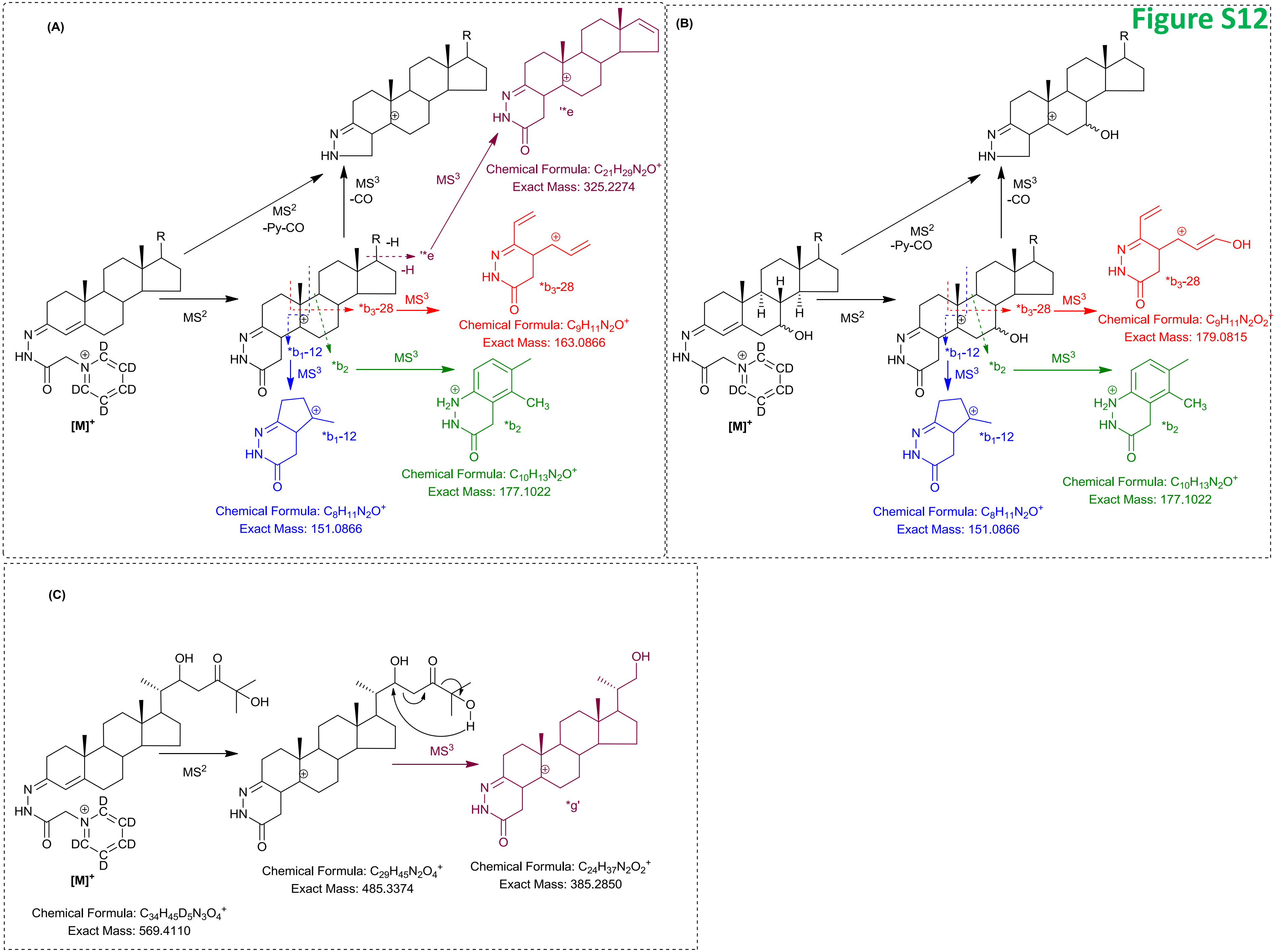

(A)
MS^2
-Py-CO
MS^3
-CO
'*e
-H
*b$_3$-28
*b$_1$-12
*b$_2$
[M]$^+$
Chemical Formula: $C_{21}H_{29}N_2O^+$
Exact Mass: 325.2274
Chemical Formula: $C_9H_{11}N_2O^+$
Exact Mass: 163.0866
Chemical Formula: $C_{10}H_{13}N_2O^+$
Exact Mass: 177.1022
Chemical Formula: $C_8H_{11}N_2O^+$
Exact Mass: 151.0866
(B)
Chemical Formula: $C_9H_{11}N_2O_2^+$
Exact Mass: 179.0815
Chemical Formula: $C_{10}H_{13}N_2O^+$
Exact Mass: 177.1022
Chemical Formula: $C_8H_{11}N_2O^+$
Exact Mass: 151.0866
(C)
*g'
Chemical Formula: $C_{34}H_{45}D_5N_3O_4^+$
Exact Mass: 569.4110
Chemical Formula: $C_{29}H_{45}N_2O_4^+$
Exact Mass: 485.3374
Chemical Formula: $C_{24}H_{37}N_2O_2^+$
Exact Mass: 385.2850
