# Supplementary material for: Formation and metabolism of oxysterols and cholestenoic acids found in the mouse circulation: Lessons learnt from deuterium-enrichment experiments and the CYP46A1 transgenic mouse
Source: J Steroid Biochem Mol Biol. 2019 Dec;195:105475. doi: 10.1016/j.jsbmb.2019.105475 (PMC6880786; doi:10.1016/j.jsbmb.2019.105475)
Supplement: Supplementary file 5 [file mmc5.docx]

**Supplemental Tables, Figures and Text**

Supplemental Figure S1. Schematic of the EADSA process illustrated by 24S-HC and 7-OC.

Supplemental Figure S2. MS^3^ ([M]^+^🡪[M-Py]^+^🡪) spectra of monohydroxycholesterols. (A) MS^3^ spectra of [^2^H_0_] (upper panel) and [^2^H_6_] (lower panel) 24S-HC. (B) MS^3^ spectra of [^2^H_0_] (upper panel) and [^2^H_6_] (lower panel) 25-HC. (C) MS^3^ spectra of [^2^H_0_] (upper panel) and [^2^H_6_] (lower panel) 24R-HC. (D) MS^3^ spectra of [^2^H_0_] (upper panel) and [^2^H_5_] (lower panel) 26-HC. (E) MS^3^ spectra of [^2^H_0_] (upper panel) and [^2^H_6_] (lower panel) 7β-HC. (F) MS^3^ spectra of [^2^H_0_] (upper panel) and [^2^H_6_] (lower panel) 7α-HC

Supplemental Figure S3. Diagnostic fragment-ions generated from 24-HC and 26-HC.

Supplemental Figure S4 MS^3^ ([M]^+^🡪[M-Py]^+^🡪) spectra of some minor oxysterols. (A) MS^3^ spectrum of a combination of [^2^H_0_]3β,x-diHC-yO + [^2^H_0_]x-HC-3,y-diO. When analysed after cholesterol oxidase treatment both compounds appear in their [^2^H_0_]x-HC-3,y-diO form. (B) MS^3^ spectrum of [^2^H_0_]3β,x,y-triHC-zO.

Supplemental Figure S5. Pathways from cholesterol to 3β,7β-diHCA. See Supplemental Text.

Supplemental Figure S6. MS^3^ ([M]^+^🡪[M-Py]^+^🡪) spectra of dihydroxy-3-oxocholest-4-enoic acids. (A) MS^3^ spectrum of [^2^H_0_] 7α,24S-diH,3O-CA. (B) MS^3^ spectra of [^2^H_0_] (upper panel) and [^2^H_3_] (lower panel) 7α,x-diH,3O-CA. (C) MS^3^ spectra of [^2^H_0_] (upper panel) and [^2^H_3_] (lower panel) 7α,12α-diH,3O-CA. (D) MS^3^ spectra of [^2^H_0_] (upper panel) and [^2^H_3_] (lower panel) 7α,25-diH,3O-CA. See Supplemental Figure S7 for fragmentation patterns of dihydroxy-3-oxocholest-4-en-26-oic acids.

Supplemental Figure S7. Fragmentation of dihydroxy-3-oxocholest-4-en-26-oic acids.

Supplemental Figure S8. MS^3^ ([M]^+^🡪[M-Py]^+^🡪) spectra of dihydroxy-3-oxocholest-4-enoic acids. (A) MS^3^ spectra of 7α,24S-diH,3O-CA from a *CYP46A1*tg mouse, the upper panel is from the earlier eluting peak, the lower panel from the latter eluting peak. (B) MS^3^ spectrum of 7α,25-diH,3O-CA from the *CYP46A1*tg mouse. (C) MS^3^ spectrum of 7α,12α-diH,3O-CA from the WT mouse. See Supplemental Figure S7 for fragmentation patterns of dihydroxy-3-oxocholest-4-en-26-oic acids.

Supplemental Figure S9. MS^3^ ([M]^+^🡪[M-Py]^+^🡪) spectra of 24S-HC and of 24S,25-EC and isomers thereof. (A) MS^3^ spectra of 24S-HC eluting at 7.50 min from the *CYP46A1*tg mouse (upper panel) and at 7.49 min from a control mouse (lower panel). (B) MS^3^ spectrum of 24S,25-EC from the *CYP46A1*tg mouse. (C) MS^3^ spectrum of 26(*E*)-HD from the *CYP46A1*tg mouse. (D) MS^3^ spectrum of 24-OC from the *CYP46A1*tg mouse.

Supplemental Figure S10. Scheme depicting isomerisation, hydrolysis and methanolysis of 24S,25-EC.

Supplemental Figure S11. MS^3^ ([M]^+^🡪[M-Py]^+^🡪) spectra of dihydroxycholesterols and dihydroxycholestenones. (A) MS^3^ spectrum of 24,25-diHC eluting at 3.37 min from the *CYP46A1*tg mouse. (B) MS^3^ spectrum of 20R,22R-diHC eluting at 3.99 min from the *CYP46A1*tg mouse. (C) MS^3^ spectra of 7α,25-diHC + 7α,25-diHCO eluting at 5.32 min (upper panel) and of 7α,24S-diHC +7α,24S-diHCO eluting at 5.42 min (lower panel) from the *CYP46A1*tg mouse. (D) MS^3^ spectrum of 7α,26-diHC + 7α,26-diHCO eluting at 5.93 from the WT mouse. (E) MS^3^ spectra of 7α,25-diHCO eluting at 5.32 min (upper panel), 7α,24S-diHCO eluting at 5.43 min (central panel) and 6.57 (lower panel) from the *CYP46A1*tg mouse. (F) MS^3^ spectrum of 7α,26-diHCO eluting at 5.95 min from the WT mouse.

Supplemental Figure S12. Fragment-ions important in the presumptive identification of oxysterols and cholestenoic acids.

**Supplemental Text**

In the absence of authentic standards, presumptive identifications were made based on exact mass, MS^3^ spectra and retention time.

3β,x-diHC-yO and x-HC-3,y-diO were presumptively identified as 3β,20-diHC-22O and 20-HC-3,22-diO or the 22-hydroxy-24-oxo isomers i.e. 3β,22-diHC-24O and 22-HC-3,24-diO (Figure 4A and Supplemental Figure 4A). The low mass fragment ions at *m/z* 151 (*b_1_-12) and 163 (*b_3_-28) indicate that derivatisation is at C-3 with a C-4 – C-5 double bond (cholesterol oxidase treatment converts 3β-hydroxy-5-ene to 3-oxo-4-ene functions, Supplemental Figure S1), the ion at *m/z* 325 (‘*e, Supplemental Figure S12A) indicates that additional substituents are on the side-chain. As the hexaduterated metabolite of [26,26,26,27,27,27-^2^H_6_]cholesterol is identified, substitution cannot be at C-26 or C-27. The high abundance of the peak at *m/z* 441 ([M-Py-CO]^+^) indicates the presence of a carbonyl group (on the side-chain) and the peak at *m/z* 451 ([M-Py-H_2_O]^+^) indicates a hydroxy group (on the side-chain, probably not at C-25). The exact positioning of substituents on the side-chain are unclear, but 20- and 22-hydroxylations occur as part of CYP11A1 catalysed side-chain cleavage to generated C_21_ steroids, while C-24 carbonylation occurs in peroxisomal side-chain shortening to C_24_ bile acids, so these substitutions are biologically feasible.

3β,x,y-triHC-zO was presumptively identified as 3β,22,25-triHC-24O, (Figure 4B, Supplemental Figure S4B). The identification is based on the fragment-ions at *m/z* 151 and 163 defining derivatisation at C-3 with a C-4 – C-5 double bond, the presence of hexadeuteration in the metabolite derived from [26,26,26,27,27,27-^2^H_6_]cholesterol indicating further substitution is not at C-26 or C-27 and the intense neutral loss corresponding to [M-Py-100]^+^ suggesting a 22,25-dihydroxy-24-oxo side-chain. Such a neutral-loss is driven by thermodynamically favourable loss of acetone and the ketene ethenone from the 22,25-dihydroxy-24-oxo side-chain (Supplemental Figure S12C).

The identification of 7α,12α-diHC,3O-CA (Figure 5B) is described in [72]. The spectra presented in Supplemental Figure S6C show that three deuterium atoms have been lost from the terminal side-chain carbon atoms, as would be expected for a C-26 acid derived from [26,26,26,27,27,27-^2^H_6_]cholesterol. The fragment-ion combination of *m/z* 151 and 179 is characteristic of a 7-hydroxy-3-oxo-4-ene structure (Supplemental Figure S12B) and the abundant fragment ions at *m/z* 422 from the [^2^H_0_] precursor and at *m/z* 425 from the [^2^H_3_] precursor (i.e. [M-Py-(H_2_O)-CONH]^+^) are characteristic of a 7,12-dihydroxy-3-oxo-4-ene steroid nucleus (Supplemental Figure S7).

It is likely that the [26,26,26,27,27,27-^2^H_6_]cholesterol fed to the mouse over the 40 day period may have contained trace amounts of [^2^H_6_]7-OC and [^2^H_6_]7β-HC, in which case they can act as substrates for enzymatic metabolism (see Supplementary Figure S5). However, the observation of a greater enrichment in deuterium in 7β-HC than in cholesterol does argue that there is some formation of this oxysterol by *ex vivo* autoxidation. This may have occurred during sample transport and storage as we have previously shown that the analytical method generates only trace quantities of 7β-HC [85].
